# Picoplankton diversity in an oligotrophic and high salinity environment in the central Adriatic Sea

**DOI:** 10.1038/s41598-023-34704-9

**Published:** 2023-05-10

**Authors:** Danijela Šantić, Iva Stojan, Frano Matić, Željka Trumbić, Ana Vrdoljak Tomaš, Željana Fredotović, Kasia Piwosz, Ivana Lepen Pleić, Stefanija Šestanović, Mladen Šolić

**Affiliations:** 1 Institute of Oceanography and Fisheries, Šetalište Ivana Meštrovića 63, Split, Croatia; 2 University Department of Marine Studies, University of Split, Ruđera Boškovića 37, Split, Croatia; 3 Department of Biology, Faculty of Science, University of Split, Ruđera Boškovića 33, Split, Croatia; 4 National Marine Fisheries Research Institute, Kołłątaja 1, Gdynia, Poland; 5 Doctoral Study of Biophysics, Faculty of Science, University of Split, Ruđera Boškovića 37, Split, Croatia

**Keywords:** Microbial ecology, Community ecology, Biooceanography

## Abstract

By combining qualitative 16S metabarcoding and quantitative CARD-FISH methods with neural gas analysis, different patterns of the picoplankton community were revealed at finer taxonomic levels in response to changing environmental conditions in the Adriatic Sea. We present the results of a one-year study carried out in an oligotrophic environment where increased salinity was recently observed. We have shown that the initial state of community structure changes according to environmental conditions and is expressed as qualitative and quantitative changes. A general pattern of increasing diversity under harsh environmental conditions, particularly under the influence of increasing salinity at the expense of community abundance was observed. Considering the trend of changing seawater characteristics due to climate change, this study helps in understanding a possible structural change in the microbial community of the Adriatic Sea that could affect higher levels of the marine food web.

## Introduction

Picoplankton play a key role in the marine environment. Through their different processes (autotrophic, heterotrophic, photoheterotrophic), they are involved in the functioning of the marine environment, especially in oligotrophic areas^1–4^. Determining the patterns of bacterial communities provides a suitable approach to assessing their response to changes in the environment^4^.

Previous studies have shown that the picoplankton community responds rapidly and differently to various changes in environmental factors, and provide effective information about the trophic status of the water column^5,6^, particularly to an increase in temperature^7^. With global warming these impacts will further strengthen the role of the microbial food web in the ocean carbon cycle^7,8^. Recently, the negative effects of increasing salinity on the picoplankton community have been noted^9^. Given this, it is important to know how individual members of the community respond to environmental changes, especially in the oligotrophic areas of the Adriatic that is phosphorus and nitrogen limited^10^.

Studies have been carried out on the proportion of individual picoplankton groups under specific environmental conditions^11–13^, but data on the year-round qualitative and quantitative response of picoplankton in general is limited. Therefore, this study provides the first comprehensive insight into the seasonal and spatial dynamics of the picoplankton community in the Adriatic Sea in terms of abundance, the dynamics of major bacterial groups, and archaeal and bacterial diversity. We assumed that changes in salinity, temperature, and nutrients have different effects on the patterns and diversity of the microbial community. To determine the relationship between the picoplankton community and biological and environmental parameters, we used neural gas^14^, an algorithm for finding optimal data representations based on feature vectors capable of extracting common information and outliers; a method that has been shown to be very successful in our previous research^12^.

## Results

### Physicochemical parameters

Sampling was conducted at three stations representing the physical and chemical characteristics of the areas studied (See SI Table 1). Temperatures ranged from 10.80 to 27.01 °C. The seasonal thermocline ranged from 5 to 30 m, formed in April and persisted until December after the vertical column had homogenized due to the strong winter pulse and energy exchange with the atmosphere. Salinity ranged from 32.43 in the nearshore surface waters to 39.02 in the intermediate layer of open waters. The lowest salinity values were found in the surface layer of the shallowest station (ST101), the one closest to the coast. The highest salinity values were measured in the deepest layer (100 m), in the open sea. In general, nutrient concentrations displayed an opposite trend compared to salinity and total phosphorus, decreasing significantly from the coast to the open sea. During the study period, the area was P-limited, but not N-limited.

**Table 1:** Diversity indices of archaeal and bacterial kingdoms per each PH-BMU.

	PHBMU1	PHBMU2	PHBMU3	PHBMU4
Number of samples	30	10	13	36
Average archaeal species number (± S.D; min, max)	41.13 (± 6.307; 25, 49)	45.9 (± 2.846; 42, 50)	27.46 (± 4.754; 19, 33)	32.03 (± 7.618; 12, 43)
Average archaeal diversity (Shannon index; ± S.D; min,max)	2.274 (± 0.247; 1.704, 2.588)	2.106 (± 0.306; 1.635, 2.637)	1.935 (± 0.379; 1.279, 2.453)	2.225 (± 0.316; 1.424, 2.637)
Average archaeal evenness (Pielou’s index; ± S.D; min,max)	0.615 (± 0.068; 0.475, 0.738)	0.55 (± 0.078; 0.437, 0.685)	0.591(± 0.132; 0.373, 0.790)	0.652 (± 0.102; 0.419,0.800)
Average bacterial species number (± S.D; min, max)	571.37 (± 33.443; 503, 631)	575 (± 27.252; 522, 608)	534.92 (± 48.769; 445, 591)	550.31 (± 49.877; 336, 616)
Average bacterial diversity (Shannon; ± S.D; min,max)	4.394 (± 0.206; 3.962, 4.973)	4.644 (± 0.236; 4.368; 5.097)	3.973 (± 0.329; 3.120, 4.275)	4.197 (± 0.232; 3.579, 4.523)
Average bacterial evenness (Pielou’s index; ± S.D; min, max)	0.692 (± 0.027; 0.636, 0.753)	0.731 (± 0.034; 0.690, 0.799)	0.632 (± 0.046; 0.508; 0.673)	0.665 (± 0.029; 0.611, 0.710)

### Biological parameters

**Figure 1:**
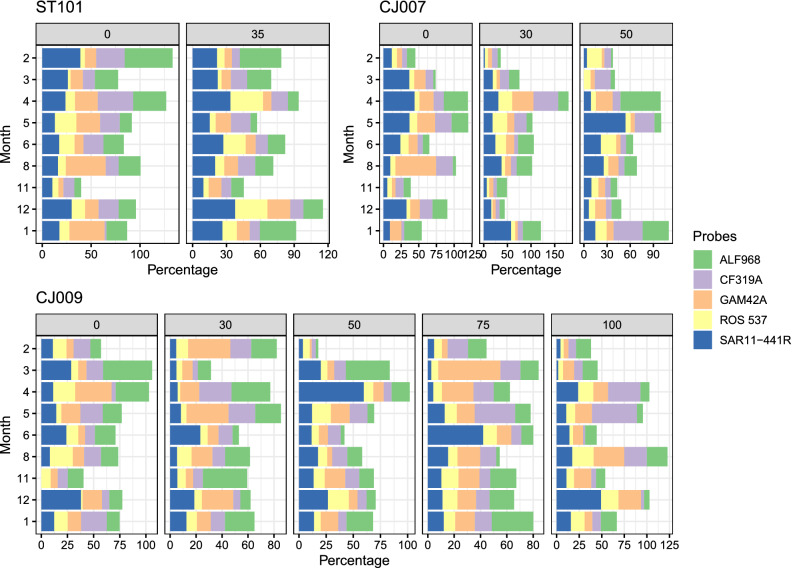
Seasonal and spatial distribution of five bacterial groups detected by CARD-FISH.

**Figure 2:**
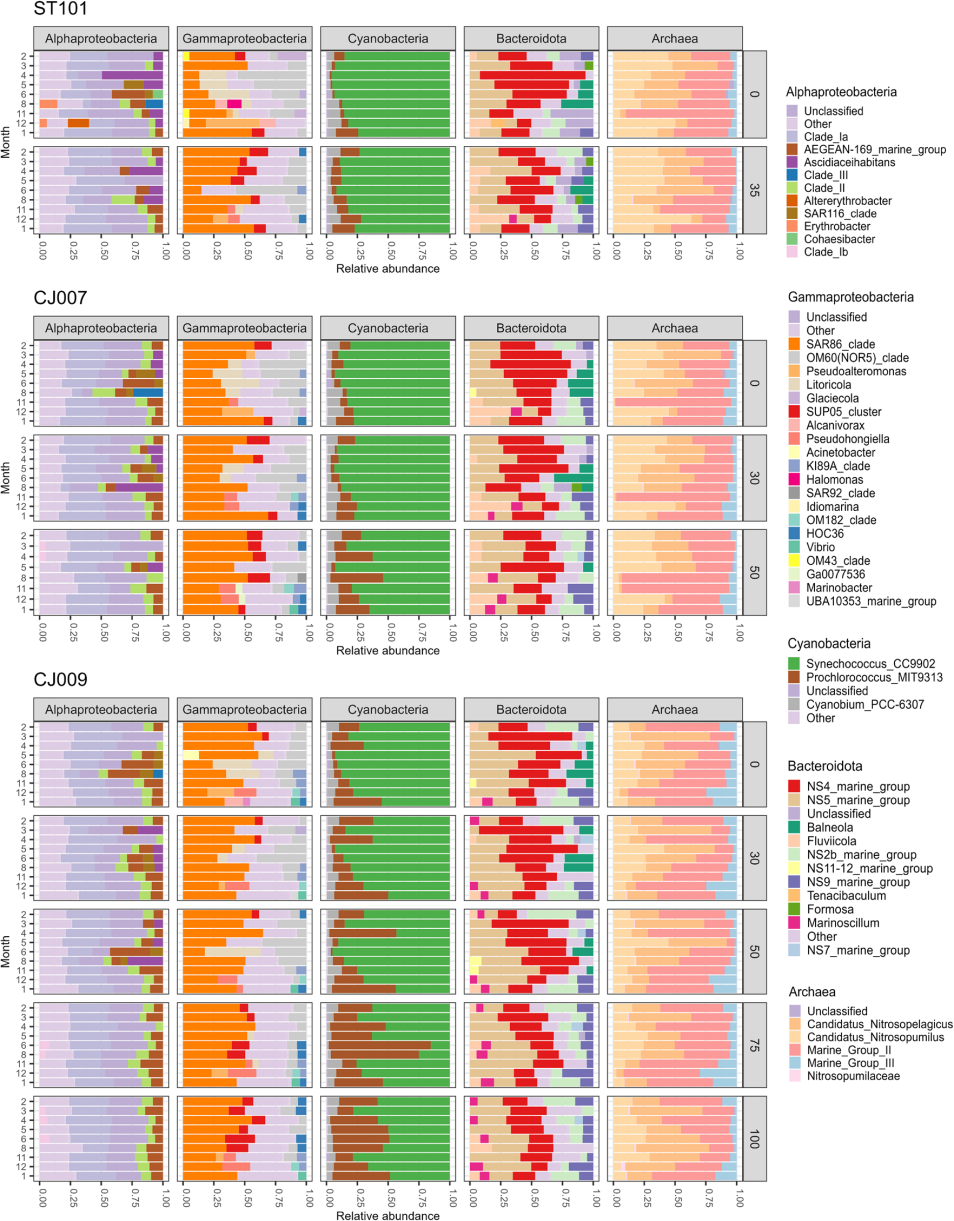
Genus level taxonomic classification of the most abundant bacterial and archaeal groups displayed at spatio-temporal scale.

For a detailed description of picoplankton community composition, abundance, and activity at temporal and spatial scales, see the Appendix (SI Figs. 1–3). The general pattern describing the proportions of the five bacterial groups detected by CARD-FISH shows the dominance of SAR11, followed by general Alphaproteobacteria, Gammaproteobacteria, *Bacteroidetes*, and finally *Roseobacter* (Fig. 1, see Supplement for a detailed description). Metabarcoding results of 16S rRNA gene with a detailed taxonomic table are also described at spatio-temporal scale (Fig. 2, SI Table 2–3, see Supplement for a detailed description). As regards the number of reads per sample and the qualitative description of the microbial community, Proteobacteria (with main classes Alpha- and Gammaproteobacteria) was by far the most predominant bacterial phylum across all samples, followed by Cyanobacteria and Bacteroidota, whilst the most abundant archaeal phyla were Thermoplasmatota and Crenarchaeota.

## Neural gas analysis of CARD FISH results

Bacterial groups (SI Fig. 5), shown as a percentage of total bacterial abundance, are classified into nine distinct classes using Neural Gas (NG), called Card Fish Best Match Units (CFBMU), which are associated with other biological and environmental factors.

SAR11 dominated in CFBMU1, where 17.8% of the data were combined. As regards biological and ecological factors, this pattern is characterised by increased proportions of *Roseobacter*, abundances of *Prochlorococcus*, picoeukaryotes, AAPs, values of bacterial production, and the highest measured Chl *a* concentration. Data were collected at depths ranging from 30 to 50 m, with the most common temperature being below 16 °C, and were completely independent of season and depth.

CFBMU2 described the most numerous pattern (22% of the data), where the same proportions of the bacterial groups analysed occurring in the environment with an average temperature slightly below 16 °C, salinity of 38.2, the highest nitrate concentration, and thus total inorganic nitrogen. This unit is described by data from different stations, seasons and depths, suggesting that it is completely independent of season and location.

In CFBMU3, Gammaproteobacteria dominated, followed by Alphaproteobacteria, *Bacteroidetes*, SAR11, and finally *Roseobacter*. These combined samples (8.9%) showed no relationship with season or spatial distribution, but this pattern is described at seawater temperature above 16 °C.

CFBMU4 represents a small group of data (5.5%) in which *Bacteroidetes* dominated. This dominance is accompanied by a high proportion of *Roseobacter,* an increased abundance of AAPs, a peak in bacterial production, and increased concentration of ammonium ions.

CFBMU5 describes a pattern of 12.2% of the samples in which the dominance of Alphaproteobacteria is evident. This pattern is found in deeper layers of the open sea with exceptionally high salinity.

CFBMU6 describes the dominance of *Bacteroidetes* and the highest proportion of *Roseobacter* in 10% of the samples. The highest proportions of AAPs and the highest SRP concentration are also associated with high seawater temperature.

Alphaproteobacteria dominated in CFBMU7 with about 40% of the DAPI counts. The highest number of bacteria was also found in this environment. This pattern of 6.6% of the data mainly described samples from the open sea or deeper coastal areas with high salinity.

CFBMU8 accounted for 8.8% of the data and SAR11 dominated with 44%. This pattern was found in the open sea and the transition station, at a temperature of around 16 °C and salinity of 38.45.

CFBMU9, the smallest unit comprising 3.3% of the samples, is characterised by the highest dominance of SAR11 and increased Alphaproteobacteria, maximum nitrate concentration, low temperature and low salinity.

### Neural gas analysis of picoplankton community composition from Illumina sequences

**Figure 3:**
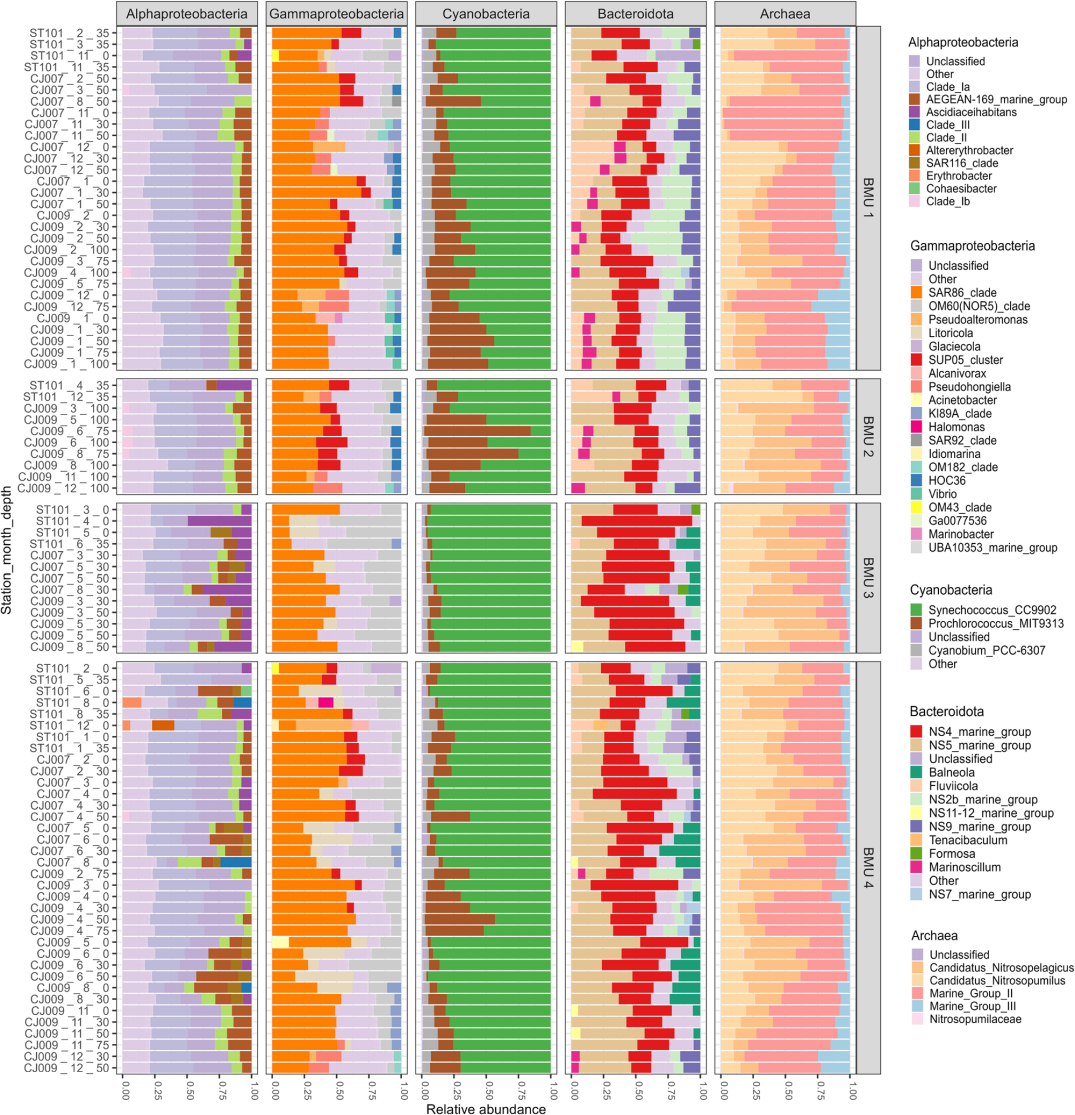
Relative proportion of genera of the most abundant bacterial and archaeal groups shown for each PHBMU (samples are labelled by station, month and depth).

The 24 observed phyla were presented as the sum of the relative abundances of OTUs, which were classified into four distinct classes called phylum best match units (PHBMU1-PHBMU4) using NG and associated with other biological and environmental factors (SI Figs. 6–7). In all four best match units, the phylum Proteobacteria dominated, followed by others. More detailed taxonomic levels for each PHBMU are presented in Fig. 3.

PHBMU1 described 33% of the sequencing data. This unit is mainly characterized by high average OTU relative abundances of phyla Thermoplasmatota, Cyanobacteria, Bacteroidota, Crenarchaeota, Actinobacteriota and Marinimicrobia. Dadabacteria, Desulfobacterota and Firmicutes exhibited the highest relative abundances in this unit. This environment could be described as the coldest one with an average temperature < 16 °C and the highest levels of ammonium ions, total nitrogen, and the highest percentage of high nucleic acid bacteria. The greatest archaeal Shannon diversity was also recorded in this unit with two main phyla Thermoplasmatota and Crenarchaeota. At genus level, the main archaeal contributor with the highest OTU relative abundances was Marine group II of Thermoplasmatota followed by *Candidatus Nitrosopumilus* and *Candidatus Nitrosopelagicus* of phylum Crenarchaeota. Marine group III exhibited the highest average relative abundances in this unit compared to others.

PHBMU2 corresponds to the smallest unit and represents approximately 11% of the observed data. This environment is characterised by exceptionally high salinity, high values of nitrates, nitrites, total nitrogen and the highest mean depth. The greatest number of bacterial species, bacterial Shannon diversity and Pielou’s evenness were recorded in this unit. Twelve phyla in this pattern exhibited the highest relative abundances compared to other units. Archaea Crenarchaeota and Thermoplasmatota reached their highest relative abundances here, followed by Chloroflexi, Nitrospinota, Planctomycetota, Gemmatimonadota, Acidobacterota and Marinimicrobia. Since bacterial diversity was the highest recorded in this unit, the most diverse phylum Proteobacteria (classes Alpha- and Gammaproteobacteria), followed by Bacteroidota and Cyanobacteria, are shown at genus level. Class Alphaproteobacteria was dominated by SAR11 Clade Ia, followed by Clade II, Clade Ib and AEGEAN-169 (order Rhodospirillales). Up to 25% of alphaproteobacterial reads in all the samples were contributed by genera with a relative abundance of < 5%. The SAR86 lineage was the major contributor to gammaproteobacterial reads, followed by OM60(NOR5), SUP05, HOC36 and genus *Pseudohongiella*. Phylum Bacteroidota was almost equally represented with NS4 and NS5 lineages, followed by higher relative abundances of NS2b and NS, similar to PHBMU1. Genera *Fluviicola* and *Marinoscillum* were observed in higher relative abundances in this BMU. Cyanobacterial reads were dominated by *Prochlorococcus* in the majority of samples within this BMU with relative abundances up to 70%.

PHBMU3 described 14% of the sequencing data and the part of the water column with the highest abundances of heterotrophic bacteria, AAPs, *Synechococcus*, picoeukaryotes, and highest bacterial production as well as chlorophyll concentration. However, the lowest archaeal and bacterial diversity was recorded in this unit. Phyla Cyanobacteria, Bacteroidota and Verrumicrobiota had the maximum relative abundances in this unit compared to other BMUs while, on the other hand, fourteen phyla exhibited the lowest ones. Alphaproteobacterial genus *Ascidiaceihabitans* (family Rhodobacteraceae) exhibited noticeably higher relative abundances with contributions of up to 50%. Gammaproteobacteria OM60 showed higher abundances compared to other units while the SUP05 clade, and HOC36, were not recorded. Bacteroidota were dominated by the NS4 group with higher relative abundances of *Balneola* and, contrary to PHBMU1 and PHBMU2, genera *Fluviicola* and *Marinoscillum* were not observed.

PHBMU4 accounted for the largest amount of sequencing data (40%). Proteobacteria led with the highest relative abundances across all units, followed by higher abundances of Bacteroidota, Cyanobacteria, Actinobacteriota, Crenarchaeota, Thermoplasmatota, Verrucomicrobiota, Marinimicrobiota and Dadabacteria. This pattern describes the highest average temperature and *Prochlorococcus* absolute abundances, bacterial production, highest archaeal evenness, and elevated concentration of nitrates and ammonium ions. At genus level, Alphaproteobacteria *Erythrobacter* was recorded in two samples in higher relative abundance up to 10% with generally greater contribution of AEGEAN-169 compared to other units. Bacteroidota were represented with higher relative abundances of *Balneola* and NS11-12 as opposed to other units.

Summarizing the results of NG analysis, we found that the phyla Proteobacteria, Cyanobacteria, Bacteroidota, Actinobacteriota Crenarchaeota, Thermoplasmatota, Verrucomicrobiota, Marinimicrobia, and Planctomycetota dominate in all four BMUs regarding relative abundances. The phyla that showed independent behaviour upon environmental changes were Proteobacteria, Bacteroidota, Actinobacteriota, Marinimicrobia, Planctomycetota, Bdellovibrionota, Desulfobacterota, Myxococcota, and NB1-j. Two Archaea, Thermoplasmatota and Crenarchaeota, showed the same pattern with higher relative abundances in a more saline environment enriched by total nitrates, while Verrucomicrobiota preferred less saline and more productive environments. The most productive environment apparently favoured Bacteroidota. In addition to two Archaea, the phyla Nitrospinota, SAR324, Chloroflexi, and Acidobacteriota showed the highest incidence in an environment with exceptionally high salinity enriched by nitrates and nitrites. Dadabacteria, Desulfobacterota and Firmicutes exhibited the highest incidence in the coldest and ammonium-rich environment.

### Diversity metrics

The rarefaction curve showed that the sequencing depth was sufficient to describe the diversity of archaea and bacteria in the studied areas of the central Adriatic Sea (SI Fig. 4). Considering the classification of phyla patterns via NG analysis into four distinct BMUs described as specific environments, we analysed the diversity of the picoplankton community according to each BMU (Table1). The average values regarding the number of archaeal and bacterial OTU counts, Shannon diversity index, and bacterial Pielou’s evenness were the lowest in PHBMU3, which is described as the environment with the highest abundance of heterotrophic bacteria, AAPs, *Synechococcus*, picoeukaryotes, bacterial production and chlorophyll concentration. On the contrary, the environment with increased salinity, nitrates, nitrites and total nitrogen (PHBMU2) showed the highest bacterial diversity and evenness. The cold and ammonium-rich environment (PHBMU1) was represented by the highest percentage of high nucleic acid bacteria and the highest archaeal Shannon index. The highest archaeal Pielou’s evenness was observed in PHBMU4.

## Discussion

Knowledge of the variability of bacterial diversity is important for understanding the relevant processes mediated by picoplankton in the marine environment. To achieve this, it is crucial to apply qualitative and quantitative molecular methods in order to obtain a more realistic picture. Therefore, our results are based on the combination of two molecular techniques, CARD-FISH and 16S rRNA gene sequencing, and the application of NG to find picoplankton community patterns and link them to ecological conditions (both biological and physicochemical). In general, the ecological conditions of the studied area can be described as oligotrophic, with reduced nitrogen and phosphorus, low productivity, and recently with a tendency of increased salinity compared to the long-term average values^9,10,15^. It is known from our previous studies that a temperature of 16 °C is considered an ecological limit^7^. Therefore, we use the same warm/cold environmental boundary for further discussion of our results.

### Quantitative analysis

According to the annual distribution of CARD-FISH proportions and the results of the NG algorithm, SAR11 dominates in this study area, followed by the rest of the Alphaproteobacteria, Gammaproteobacteria, *Bacteroidetes,* and *Roseobacter*. SAR11 has also been a strong contributor to the bacterial communities in the Adriatic province in previous studies^11,16^. This ultra-small bacterium, Earth’s ubiquote^17^ dominates in oligotrophic environments and can account for up to 33% of many euphotic zone communities^18,19^. The life strategy of SAR11 is that of a K-strategist (slow growing and relatively dominant). To cope with oligotrophic conditions, free-living organisms have streamlined their metabolism and genome, thereby reducing their nutrient requirements in poor environments^20,21^. The area studied is characterized by low bacterial production and the predominance of LNA bacteria^22^. Mary et al.^23^ discovered that LNA bacteria dominate in the SAR11 bacterial group, which is consistent with the hypothesis of Santos et al.^24^, i.e. that slow-growing bacteria are more resistant to environmental factors. SAR11 was found to prevail in the CFBMU1 environment, which can be described as more productive based on its biological and physical characteristics (high proportion of *Roseobacter*, *Prochlorococcus*, picoeukaryotes, AAPs, higher bacterial production and higher Chl *a* concentration at a temperature of around 16 °C). In addition, SAR11 also predominated in the open sea with exceptionally high salinity (CFBMU8) and in the colder environment with lower salinity and increased nitrate content (CFBMU9). The summary of our data suggests that this group is composed of eurivalent microorganisms that successfully inhabit different variants of the generally oligotrophic area.

Alphaproteobacteria is probably the best-known and most abundant of all marine Proteobacteria represented by numerous orders^25^. NG analysis has shown that Alphaproteobacteria dominate in deeper and saltier waters. Our results also show that this group exhibits seasonal dynamics, as reported by Teira et al.^26^, with a dominance in winter as found in the English Channel^23^. In general, their higher contribution can be explained by the fact that they are good competitors in nutrient-poor conditions^27^. Previous studies have shown the importance and dominance of this group in the Adriatic Sea^12^ and Mediterranean coastal waters^19^.

The dominance of *Bacteroidetes* in the bacterial community is described by two BMUs (CFBMU4, CFBMU6) evidencing their dominance together with the high abundance of *Roseobacter*, AAPs, maximum bacterial production, highest SRP concentration, high ammonium content and elevated temperature. All these describe a warm and nutrient-enriched environment. In addition, the seasonal distribution together with a significant relationship with temperature show a prevalence in spring and a dominance of this group in the deep maximum chlorophyll layer regardless of the season. *Bacteroidetes* are known to occur in a variety of marine environments^28^. However, they are most abundant in eutrophic environments and are associated with high bacterial biomass, production, and algal blooms^26,29–31^.

The results presented here show that the class Gammaproteobacteria may be significant at higher temperatures. Previous studies have found an increase in Gammaproteobacteria and *Bacteroidetes* in warmer seasons, which has been linked to phytoplankton blooms, dissolved organic carbon, and total bacterial counts^11,19,23,26,31–33^. In addition, Teira et al.^26^ assumed that the abundance of this group in coastal ecosystems may be related to water column stability, i.e. abundance is lower in winter when the water column is more exposed to the influence of vertical mixing. *Roseobacter* occur in smaller numbers compared to other bacterial groups, but their contribution cannot be considered negligible. The BMU that describes *Bacteroidetes* also describes this group, CFBMU4 and CFBMU6, including high numbers of *Roseobacter* in the environment with the highest AAP abundance, bacterial production, higher concentration of ammonium ions, maximum concentration of SRP, and warm seawater. Considering spatial and seasonal distribution, higher abundance was observed in coastal areas than in the open sea, while higher levels were found in the open sea during summer. The higher contribution of *Roseobacter* in summer has been reported previously^23,26,31^. Many authors point out the importance of this group in coastal areas^34–36^ or suggest that *Roseobacter* prefer nutrient-rich conditions^19,37^. As in our study, many previous studies have shown that this metabolically susceptible group is dependent on an environment with sufficient phosphorus^13,30,38,39^ and can reach up to 20% in coastal areas and 15% in the open sea, 30% and 20%, respectively, in our case. Seasonally, SAR11 dominated in spring and fall, when the Redfield ratio (61.89) shows that this is the most oligotrophic period in an otherwise nutrient-poor environment, which is consistent with previous findings in the Adriatic and Mediterranean Seas^11,19^. However, succession is observed between SAR11 and the rest of the Alphaproteobacteria (that peaked in winter and at the highest salinity), and between SAR11 and Gammaproteobacteria (that peaked in summer). *Bacteroidetes* and *Roseobacter* show the same response in the warmer and nutrient-enriched environment with increased proportions in the community. Our results show that SAR11 generally leads under different ecological conditions.

The NG results show that, in general, dominance patterns are not related with time and space, but are due to a shift in relative abundance or a favourable combination of ecological factors. Therefore, our largest pattern, CFBMU2, in which equal proportions of bacterial groups are present, can be described as an initial ‘stand-by’ state awaiting stimulation by various environmental factors^40^. The results of this study are in line with our recent findings^6^, i.e. that the clusters obtained are not spatially or temporally fixed. Rather, the area studied represents a mosaic of different environmental conditions alternating from one state to another on a time scale. In each of the environmental states, a distinct microbial food web structure evolves that exhibits consistent and repeatable changes closely associated with the changes in environmental conditions from one state to another.

### Qualitative analysis

Based on the notion that microbial food web structure evolves in response to changing environmental conditions, we used a second NG for metabarcoding data that allowed us to successfully determine the environment in which particular microbial members predominate or in which their representation decreases. Like previous NG, this analysis shows that, in general, dominance patterns are not related to time and space, but are due to a shift in relative OTU abundances or a favourable combination of environmental factors. As regards higher taxonomic levels, the phyla that displayed independent behaviour in relation to environmental change or dominance in all four BMUs include Proteobacteria, Cyanobacteria, Bacteroidota, Actinobacteriota, Verrucomicrobiota, Marinimicrobia, and Planctomycetota. Thus, we can conclude that these are truly eurivalent organisms adapted to different changes in the properties of seawater. A similar situation was observed in our previous research, where the results showed the dominance of Proteobacteria and Cyanobacteria, followed by Bacteroidota and Actinobacteriota, while changes at finer taxonomic levels were related to different environmental conditions at different depths^12^. The responses of other observed phyla may be related to different thermohaline properties of seawater and nutrient availability. Two archaeal phyla, namely Thermoplasmatota and Crenarchaeota, are found in significant numbers across the different environments as well as in the Southern Ocean^41^. Here, however, maximum abundances were observed in the environment with the highest average salinity values. As far as we know, this is the first spatio-temporal pattern of the Archaea in the Adriatic, represented by the phyla Crenarchaeota and Thermoplasmatota. Besides the Archaea, the phyla Nitrospinota, SAR324 and Chloroflexi exhibit the highest incidence in the environment with the highest average salinity, enriched with nitrates and nitrites. Although nowadays they are reported as ubiquitous in a variety of different environments, and not solely in the extremes as previously thought, our knowledge of the Archaea is very limited due to the lack of cultured representatives and the low number of sequenced genomes^42,43^. Archaea are among the most abundant microbes in the ocean, accounting for more than 30% of all marine picoplankton, regardless of light^44^. Despite their widespread occurrence in marine habitats and importance in global biogeochemical cycles of carbon fixation, nitrogen remineralization, and nitrous oxide production, little is known about phylum Crenarchaeota, also known as Thaumarchaeota, or Marine Group I^45^. *Candidatus Nitrosopumilus*, found in deep-sea and salty environments, is reported to be a chemolithoautotrophic genus of Crenarchaeota (Thaumarchaeota) that utilizes mainly ammonia and urea as a source of energy^46^. This archaeon is known to have an obligate salt requirement, with optimal growth at 25 to 37‰ salinity and pronounced light sensitivity. Thus, it is expected to be found mostly in deeper layers of the sea; however, we recorded it at all stations and depths^47^. *Candidatus Nitrosopelagicus*, found in all samples in our survey but with the highest average relative abundances below 50 m, is also a chemolithoautotrophic ammonia-oxidizing archaeon that was only recently described as ubiquitously present in the open ocean with reported genomic and proteomic characterization^48^.

Thermoplasmatota, another highly abundant and crucial archaeal phylum in the marine nitrogen cycle, found in all of our samples, was mostly represented with subdivision Marine Group II recently placed in the order-level lineage of Poseidoniales and to a lesser extent with Marine Group III^42,43,49^. Members of Marine Group II, with still no precise ecological role defined, are considered to be the most abundant archaeal heterotrophs on ocean surfaces and in the deep chlorophyll maximum (DCM) worldwide, with pronounced lower abundance in deeper layers and noticeable spatio-temporal variations^50^. On the other hand, their likely close relatives of Marine Group III, considered to be present in low-abundance at mesopelagic and bathypelagic zones, are capable of proteorhodopsin-based photoheterotrophy^49^. With only a few studies reporting Marine Group III in the photic zone, we found members of this archaeal group at all depths up to 100 m with pronounced higher abundances in deeper layers and in winter^49^. In agreement with the previous study conducted in the coastal Mediterranean Sea, we recorded the highest archaeal relative abundances in December and January at all stations with very low occurrence during summer, except at depths of 75 and 100 m^51^. Moreover, Archaea, Acidobacteriota, Chloroflexi, Gemmatimonadota, Marinimicrobia, Nitrospinota and Planctomycetota show the highest incidence in deeper and extremely saline environments enriched with nitrates and nitrates.

The Verrucomicrobiota pattern was the opposite of that of Archaea, with higher relative abundance in a warmer and more productive environment. Their cell abundance was coupled with the onset of the algae bloom and accounted for up to 8% of the bacterioplankton^52^.

Bacteroidota apparently prefer the most productive environment, as previously shown^19,53^. They are thought to play an important role in the decomposition of organic matter in the study areas^12,54^.

### Picoplankton diversity pattern

In plankton ecology, the fundamental question is how large numbers of competing species can coexist in marine ecosystems with an apparently limited variety of resources. However, in most aquatic systems they coexist throughout the year and even in summer when nutrients are depleted^55,56^. In general, the results obtained here confirm the “plankton paradox”. Namely, this study shows that diversity for both Archaea and Bacteria is lowest in the environment with the highest abundance of picoplankton members, bacterial production, and chlorophyll concentration as in our previous study^12^. In addition, this study revealed that more species of bacteria exist in saline environments that are richer in nitrates, nitrites, and SRP, while a greater diversity of Archaea with the highest Shannon index is recorded in the coldest environment with increased concentrations of ammonium and total nitrogen. In this generally oligotrophic environment, the greatest diversity of bacteria was found in the deepest layer, confirming the results of previous studies^11,12,57,58^.

## Conclusions

Summarizing our results, we can conclude that, at CFBMU level, equal proportions of bacterial groups can be described as an initial ‘stand-by’ state awaiting stimulation by different ecological conditions. Specifically, we conclude that SAR11 is eurivalent and that, generally speaking, it has a leadership position under different conditions. However, other Alphaproteobacteria show a positive response to increased salinity, while Gammaproteobacteria dominate when temperature increases. *Bacteroidetes* and *Roseobacter* increase their populations under warmer and nutrient-rich conditions.

According to the PHBMU results, the phyla Proteobacteria, Cyanobacteria, Bacteroidota, Actinobacteriota, Crenarchaeota, Thermoplasmatota, Verrucomicrobiota, Marinimicrobiota, and Planctomycetota are also eurivalent organisms adapted to changes in seawater properties.

The responses of other observed phyla may be associated with different thermohaline properties and nutrient availability. Two archaeal phyla, namely Thermoplasmatota and Crenarchaeota, were found in significant numbers cross the different environments, but they showed elevated values in a saltier environment enriched with total nitrates. On the other hand, Verrucomicrobiota, display the opposite pattern with higher abundances in a less saline and more productive environment. The most productive environment appeared to favour Bacteroidota. In addition to Archaea, the phyla Nitrospinota, SAR324, Chloroflexi, and Acidobacteriota had the highest incidence in the environment with exceptionally high salinity, enriched with nitrates and nitrites. Dadabacteria, Desulfobacterota and Firmicutes showed the highest incidence in the coldest and most ammonium-rich environment.

We found a general pattern of increasing diversity under harsh environmental conditions, particularly under the influence of increasing salinity at the expense of microbial community abundance. The results of the diversity patterns of picoplankton suggest different strategies adopted by microorganisms to cope with changing environmental conditions.

In the face of ongoing climate change, this study helps to understand a possible structural change in the microbial community of the Adriatic Sea that could affect higher trophic levels of the marine food web.

## Material and methods

### Sampling sites and environmental conditions

**Figure 4:**
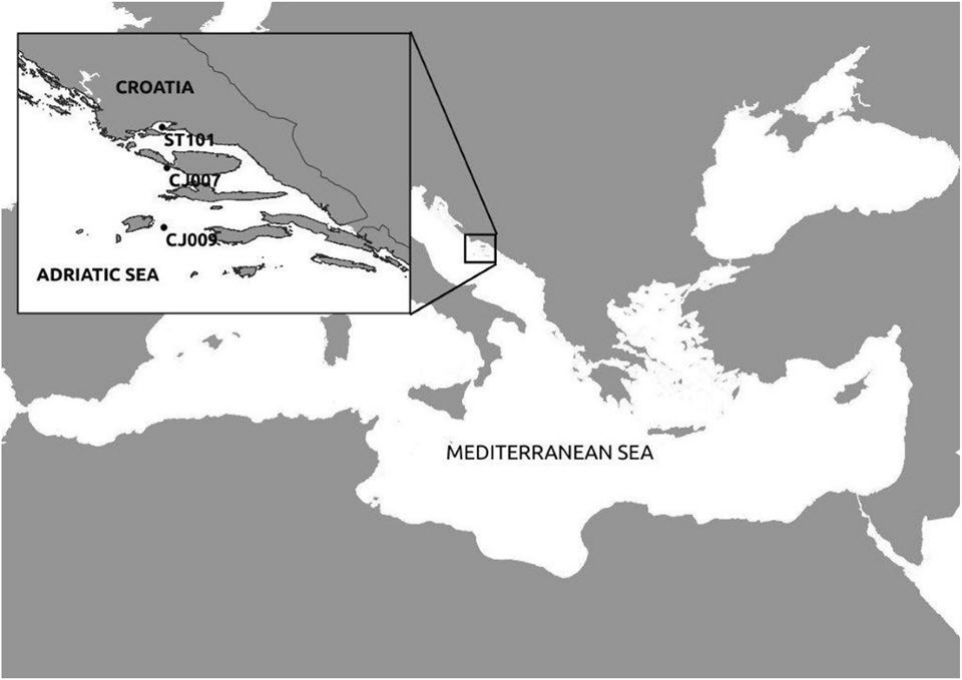
Map of the Mediterranean Sea with an enlarged view of the study area, and the sampling stations.

A total of 90 samples were collected monthly in February 2021/January 2022 on vertical profiles at three stations in the central Adriatic, the open sea area, CJ009 (0 m, 30 m, 50 m, 75 m, 100 m and deep chlorophyll maximum), and coastal waters, CJ007 (0 m, 30 m and 50 m), ST101 (0 m and 35 m) (Fig. 4).

Temperature, salinity, nutrient concentration and chlorophyll *a* (Chl *a*) were measured. Please see Supplemental Information (SI Table 1.).

### Picoplankton analysis

All members of the picoplankton community (*Synechococcus*, *Prochlorococcus*, picoeukaryotes, heterotrophic bacteria, high (HNA) and low nucleic acid content (LNA) bacteria that together represent the overall bacterial community) including heterotrophic nanoflagellates (HNF) and Aerobic Anoxygenic Phototrophs (AAPs) were determined using flow cytometry^59^ and infra-red microscopy^60^. Bacterial production was estimated using the 3H-thymidine method^61^. For details, please see Supplemental Information.

#### Fluorescence in situ hybridization followed by catalysed reporter deposition—CARD-FISH

CARD-FISH and epifluorescence microscopy were used for the enumeration of general bacteria (EUB338 I-III)^62^ and five bacterial groups, SAR11 clade (SAR11_441R)^18^, Alphaproteobacteria (ALF968), Gammaproteobacteria (GAM42a)^63^, members of the Cytophaga–Flavobacteria lineage of *Bacteroidetes* (CF319a)^64^ and *Roseobacter* clade (ROS537)^65^. The standard protocol^66^ was followed with slight modifications. For details, please see Supplemental information.

#### Sampling, DNA extraction and Illumina sequencing

Seawater was prefiltered through a 20 µm plankton net and 1–2 L were immediately vacuum-filtered through 0.22 µm polyethersulfone membrane filters (PES, 47 mm in diameter, FiltraTECH, France, Ref. MF047PE022). After filtration, the filters were promptly frozen in liquid nitrogen and stored at −80 °C until analysis. For DNA extraction, a modified DNeasy PowerWater Kit (QIAGEN) was used with the altered first step of enhanced bead beating. PES filters were cut in half with a sterilized scalpel and placed in 1.5 mL tubes filled with ceramic beads (MagNA Lyser Green Beads, Roche) followed by rigorous homogenization with MagNALyser Instrument, twice for 20 s at 6000 rpm. After homogenization, the tubes were centrifuged for 1 min at 3800 rpm. The supernatant was transferred to a clean 2 mL collection tube and the protocol was performed according to the manufacturer’s instructions (from Step 8 in Quick-Start Protocol). Total extracted DNA was quantified and qualities (A260/A280 and A260/A230 absorbance ratios) were measured using a DS-11 Spectrophotometer (Denovix).

For the amplification of the V4-V5 hypervariable regions of the 16S rRNA gene, the 515F-Y (5′-GTGYCAGCMGCCGCGGTAA-3′) and 926R (5′-CCGYCAATTYMTTTRAGTTT-3′) primer pair was used, since it can capture the diversity of both bacterial and archaeal communities^67^. Phusion® High-Fidelity PCR Master Mix (New England Biolabs) was used for PCR amplification with one-step PCR, library preparation, and 250 bp pair-end sequencing on Illumina NovaSeq 6000 performed at Novogene Europe (Cambridge, UK). The number of raw reads per sample ranged from 58.138 to 291.926 (mean ± st. dev, 134.328 ± 38.181).

#### Bioinformatics

Paired-end reads were assigned to each sample according to their unique barcodes. After barcode and primer sequences removal, FLASH (v.1.2.7) was used for merging pair-end reads based on reads overlap^68^. QIIME (v.1.7.0) quality-controlled process under specific filtering conditions was applied to filter merged reads and obtain high-quality clean tags^69,70^. After quality control, clean tags were compared against the SILVA 138 database using UCHIME algorithm (v.7.0.1001) to detect and discard chimera sequences^71,72^. Non-chimeras were assigned as effective tags used in subsequent analysis and clustered with Uparse software (v.7.0.1001) into OTUs based on the 97% similarity threshold^73^. The initial, as well as the final number of reads per sample after quality control and filtering, are given in the Supplementary Material (SI Table 2). Representative sequence assigned to each OTU was screened for further taxonomic annotation with the SSU rRNA database of SILVA138 with QIIME (v.1.7.0) in Mothur method based on the naive Bayes classifier^74,75^. The OTU matrix and assigned taxonomy table were imported in R statistical software (https:// cran.r- proje ct. org/, v.4.0.2) to create a phyloseq object with the phyloseq package (v.1.32.0)^76^. Non-bacterial OTUs classified as “chloroplast” or “mitochondria”, as well as rare OTUs seen less than ten times in at least three samples, were discarded from further analyses^76^. The OTU abundance matrix was finally rarefied to 39 680 counts per sample (according to the sample with the least sequences obtained) using function rarefy_even_depth from phyloseq package (rngseed = 150517, random number seed for reproducibility). The final number of OTUs obtained was 814 while the total number of reads was reduced to 3 531 520. For graphical displays of bacterial community composition at a specific taxonomic rank, the R package ggplot2 v3.3.5 was used^77^.

#### Calculation of diversity and evenness

To calculate archaeal and bacterial, Shannon’s (H’) and Pielou’s (J’) diversity indices based on the rarefied but untransformed OTU table, the vegan package (v2.5.7) in R was used^78^.

### Neural gas

To extract characteristic distribution (different bacterial groups /phylum), an artificial neural network algorithm called neural gas (NG) was used as NG trains an arbitrary number of winning neurons (called BMUs) by unsupervised learning^14,79–82^. NG requires no prior knowledge of the topological structure of the data manifold with weak connectivity between neural units allowing them to move freely through the data space, making NG a suitable tool for modelling anomalies and the mean distribution of the analysed microbiological parameters^12,83^.

In the study we created two separate NG models. The first model (called CARD FISH Best Mach Units, CFBMU) was constructed for extracting nine characteristic distributions of the picoplankton community, and the second one (called PHYLA Best Match Units, PHBMU) for extracting four characteristic phyla patterns. The NG was run with a reduced sample size using only the picoplankton community for quantifying data space to create nine winning neurons. For both NG models, the ecological properties are shown as an average over particular BMU space. Both NG models were initialized by setting the number of training epochs to 1000, the initial step size to 0.5, and the initial decay constant to 4.5. For NG calculation, we used the SOM Toolbox version 2.0 for MATLAB (E. Alhoniemi, J. Himberg, J. Parhankangas, and J. Vesanto, Helsinki University of Technology, Finland: http://www.cis.hut.fi/projects/somtoolbox).

The data matrix for both the CFBMU and PHBMU NG models was structured as follows: The row represents an oceanographic sample with 24 or 46 elements. The elements of the sample are: Bacterial groups (ROS537, SAR11_441R, GAM42A, CF319A, ALF968) or phyla, biological parameters (heterotrophic bacteria-UHB, High nucleic acid bacteria-HIGH, *Synechococcus-*SYN, *Prochlorococcus-*PROCHL, picoeukaryotes-PE, bacterial production-BP, hetrotrophic nanoflagellates-HNF, aerobic anoxygenic phototrophs-AAP) and environmental factors (temperature-Temp, salinity-Sal, nitrates-NO_3_^-^, nitrites-NO_2_^-^, ammonium ion-NH_4_^+^, dissolved inorganic nitrogen-DIN, total nitrogen-NTOT, soluble reactive phosphorus-SRP, total phosphorus-PTOT, silicate-SiO_4_^2-^, Chlorophyll *a*-Chl *a*).

## Supplementary Information

Supplementary Information.

## Data Availability

The raw sequence data are deposited in the NCBI database under Biosamples with accession numbers SAMN32245823-SAMN32245911 as part of BioProject PRJNA912619.
