# Supplementary material for: Picoplankton diversity in an oligotrophic and high salinity environment in the central Adriatic Sea
Source: Sci Rep. 2023 May 10;13:7617. doi: 10.1038/s41598-023-34704-9 (PMC10172355; doi:10.1038/s41598-023-34704-9)
Supplement: Supplementary file 1 — Supplementary Information. [file 41598_2023_34704_MOESM1_ESM.docx]

**Picoplankton diversity in an oligotrophic and high salinity environment in the central Adriatic Sea**

**Supplement information**

Danijela Šantić^1^, Iva Stojan^1,5^*, Frano Matić^2^, Željka Trumbić^2^, Ana Vrdoljak Tomaš^1^, Željana Fredotović^3^, Kasia Piwosz^4^, Ivana Lepen Pleić^1^, Stefanija Šestanović^1^, Mladen Šolić^1^

^1^ Institute of Oceanography and Fisheries, Šetalište Ivana Meštrovića 63, Split, Croatia

^2^ University Department of Marine Studies, University of Split, Ruđera Boškovića 37, Split, Croatia

^3^ Department of Biology, Faculty of Science, University of Split, Ruđera Boškovića 33, Split, Croatia

^4^ National Marine Fisheries Research Institute, Kołłątaja 1, Gdynia, Poland

^5^ Doctoral Study of Biophysics, Faculty of Science, University of Split, Ruđera Boškovića 37, Split, Croatia

*Iva Stojan, Institute of Oceanography and Fisheries, Šetalište Ivana Meštrovića 63, Split, Croatia, e-mail: [stojan@izor.hr](mailto:stojan@izor.hr)

## Material and methods

### 2.1. Environmental parameters

Temperature and salinity were measured with a SeaBird 25 CTD profiler with an accuracy of > ± 0.01°C and ± 0.02, respectively. Concentrations of inorganic nutrients were determined using the modified autoanalyzer method (1). The detection limits for the method were 0.001 µmolL^-1^ for nitrites (NO_2_^-^), 0.01 µmolL^-1^ for nitrates (NO_3_^-^), 0.0002 mgL^-1^ for ammonium ion (NH_4_^+^), and 0.02 µmolL^-1^ for soluble reactive phosphorus (SRP), respectively. The criteria for stoichiometric nutrient limitation indicate that ratios of dissolved N/P, 10 and Si/N, 1 indicate stoichiometric N limitation, Si/N, 1 and Si/P, 10 indicate Si limitation, and N/P, 22 and Si/P, 22 indicate P limitation (2). Chlorophyll *a* (Chl *a*) was determined from 500-mL subsamples filtered through Whatman GF/F glass fiber filters and stored at -20°C. The filters were homogenized, extracted in 90% acetone, and then analyzed fluorometrically using a Turner TD -700 laboratory fluorometer calibrated with pure Chl *a* (Sigma) (3).

### 2.2. Flow cytometry

For the flow cytometry count of autotrophic cells, 2 mL of preserved samples in 0.5% glutaraldehyde were frozen at -80 °C and stored until analysis on Cytoflex cytometer. Autotrophic cells were divided into groups: *Synechococcus*, *Prochlorococcus* and picoeukaryotes, distinguished according to light scattering, red emission of cellular chlorophyll content and orange emission of phycoerythrin-rich cells. Abundances of Sybr Green-I-stained bacteria, high nucleic acid content (HNA) bacteria, low nucelic acid bacteria (LNA) and heterotrophic nanoflagellates (HNF) were also determined using flow cytometry (4) and the samples were preserved in 2% formaldehyde and stored at 4 °C until analysis.

### 2.3. Aerobic Anoxygenic Phototrophic abundance - AAP

AAP cells were collected on 0.2 µm polycarbonate filters by filtration and stained using DAPI (fc.1 µgmL^-1^) using a 3:1 mixture of Citifluor™ AF1 and Vectashield® after drying (5). AAP bacteria were counted using an Olympus BX51 microscope equipped with an Olympus UPlanSApo 100×/1.40 OIL, IR objective and software for the picture analysis (CellSens). The microscope was equipped with a Hg Lamp U-LH100H6 for excitation. Fluorescent images were taken using an XM10-IR camera. Three epifluorescent filter sets were used: DAPI, IR and chlorophyll. The Chl *a* signal was subtracted from the IR image to obtain a net count of AAP cells.

### 2.4. Bacterial production

Bacterial cell production was estimated by measuring the incorporation of 3H-thymidine into bacterial DNA (6), which was added to 10 mL samples at a final concentration of 10 nmol (specific activity: 86 Ci mmol^–1^). Samples and a formaldehyde-killed absorption control (f.c. 0.5%) were incubated for one hour. The incubations were stopped with formaldehyde (f.c. 0.5%). The thymidine samples were extracted with ice-cold trichloroacetic acid (TCA). The TCA-insoluble fraction was collected on to 0.2 μm pore size polycarbonate filters. Samples were treated with Cocktail Filter Count (Perkin Elmer) before analysis on a liquid scintillation analyzer TriCarb 4910 TR (Perkin Elmer).

### 2.5. Fluorescence *in situ* hybridization followed by catalyzed reporter deposition

Fluorescence *in situ* hybridization followed by catalyzed reporter deposition (CARD-FISH) was performed according to the standard protocol (7) with slight modifications.

After fixation of samples with formaldehyde (f.c. 2%), samples were filtered on white 0.2 µm polycarbonate filters (47 mm diameter, Whatman® Nuclepore™ Track-Etched, Merck) that were immediately stored after filtration at −20°C until further analyses. After digestion with lysozyme (10 mg ml-1, 1 h at 37°C) and hybridization with the horseradish peroxidase labeled oligonucleotide probes (2 h at 35°C, in the dark), fluorescence signals were amplified with fluorescein tyramide (30 min at 37°C, PerkinElmer). To estimate the presence of nonspecific binding of probes and nonspecific substrate reaction with endogenous enzymes or pseudoperoxidase activities, NON338 probe was used. Samples were counterstained with 4’,6-diamidino-2-phenylindole (DAPI, 1 µgmL^-1^) using a 3:1 mixture of Citifluor™ AF1 and Vectashield® after drying. Bacteria were counted on Olympus BX51 epifluorescent microscope and at least 600 DAPI-stained cells per sample were counted. The abundance of each bacterial group was calculated as the ratio of hybridized cells to DAPI-stained ones.

## Results

### 3.1. Environmental conditions

SI Table 1. Summary of physicochemical parameters collected in the central Adriatic Sea (range and med‒median, T‒temperature, S‒salinity, NO_3_^-^‒nitrates, NO_2_^-^‒nitrites, NH_4_^+^‒ammonium ions, NTOT‒total nitrogen, SRP‒soluble reactive phosphorus, PTOT‒total phosphorus, SiO_4_^-^‒silicate, Chl *a*‒chlorophyll *a*).

|  | T  °C | S | NO_3_^-^  µmol L^-1^ | NO_2_^-^  µmol L^-1^ | NH_4_^+^  µmol L^-1^ | NTOT  µmol L^-1^ | SRP  µmol L^-1^ | PTOT  µmol L^-1^ | SiO_4_^-^  µmol L^-1^ | Chl a  mg m^-3^ |
| --- | --- | --- | --- | --- | --- | --- | --- | --- | --- | --- |
| Min | 10.801 | 32.434 | 0.136 | 0.004 | 0.000 | 5.503 | 0.000 | 0.069 | 0.111 | 0.040 |
| Max | 27.010 | 39.024 | 7.817 | 0.841 | 0.690 | 32.658 | 0.137 | 1.019 | 7.433 | 1.630 |
| Med | 15.012 | 38.661 | 0.976 | 0.138 | 0.198 | 1.363 | 11.892 | 0.031 | 0.140 | 1.695 |

### 3.2. Autotrophic component

Autotrophic picoplankton were most abundant in winter 2021-spring period, reaching highest abundances in the coastal area (*Synechococcus* to a maximum abundance of 1.15 x 10 ^5^ cells mL^-1^, *Prochlorococcus* to a maximum abundance of 2.09 x 10^6^ cells mL^-1^, picoeukaryotes up to 7.79 x 10^4^ cells mL^-1^) and decreasing towards the open sea. In summer and fall, *Prochlorococcus* and picoeukaryotes showed an opposite trend with higher values in the open sea (SI Fig. 1).


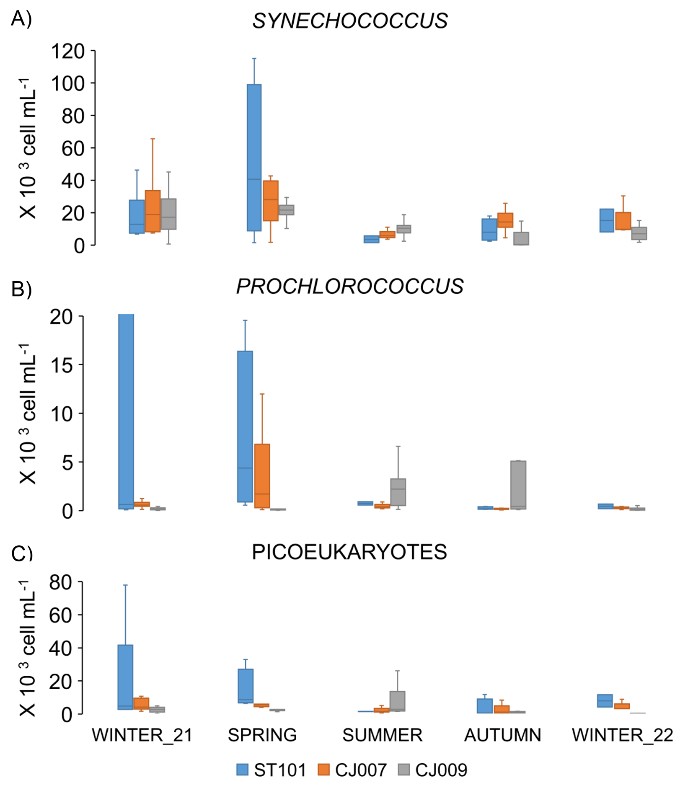


SI Figure 1. Seasonal and spatial distribution of autotrophic component: A) *Synechococcus* B) *Prochlorococcus* C) picoeukaryotes (the middle line of the box represents the median. The bottom line of the box represents the median of 1st quartile. The top line of the box represents the 3rd quartile. The whiskers extend from the ends of the box to the minimum and maximum value).

### 3.3. Heterotrophic component

Bacterial abundance ranged from 0.19 x 10^6^ on the open sea to 0.83 x 10^6^ cell mL^-1^ in the coastal region, with no seasonal variation, but a visible gradient of higher values from the coast to the open sea (SI Fig. 3). Bacterial production ranged from the minimum at CJ009 (0.04 x 10^4^ cell h^-1^ mL^-1^) to the maximum at ST101 (0.57 x 10^4^ cell h^-1^ mL^-1^). Seasonal changes show increased values in the fall and winter of 2022. Looking at the nucleic acid content of the bacteria, LNA% bacteria dominate in the observed area. However, the HNA content also shows spatial-seasonal changes in the coastal area. The highest percentage of HNA was mostly in winter, the lowest in summer (SI Fig. 2).

*
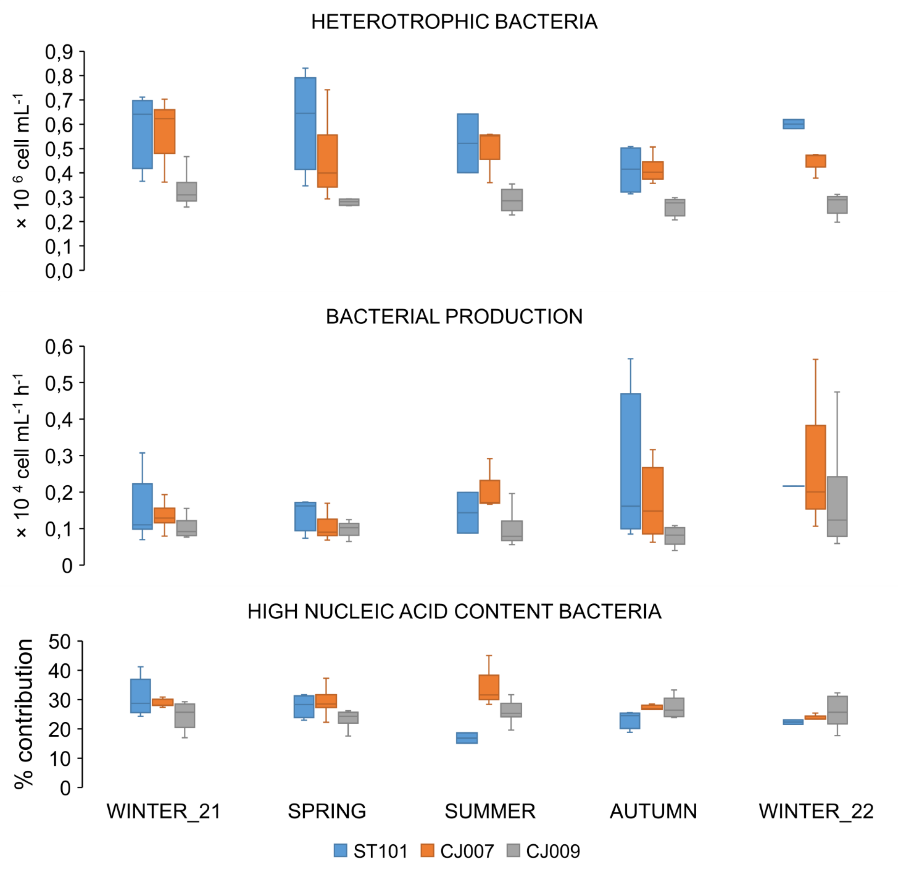
*

SI Figure 2. Seasonal and spatial distribution of heterotrophic component: A) heterotrophic bacteria B) bacterial production C) HNA bacteria (the middle line of the box represents the median. The bottom line of the box represents the median of 1st quartile. The top line of the box represents the 3rd quartile. The whiskers extend from the ends of the box to the minimum and maximum value).

### 3.4. Aerobic anoxygenic photoheterotrophs

Abundance of AAPs (SI Fig. 3) was apparently highest on the coastal area in spring and autumn (up to 3.40 x 10^4^ cell mL^-1^). On the open sea, however, abundances were increased in summer (up to 2.98 x 10^4^ cell mL^-1^).


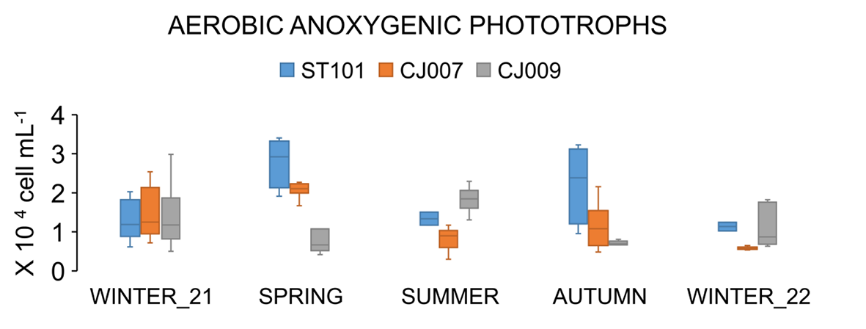


SI Figure 3. Seasonal and spatial distribution of aerobic anoxygenic phototrophs (the middle line of the box represents the median. The bottom line of the box represents the median of 1st quartile. The top line of the box represents the 3rd quartile. The whiskers extend from the ends of the box to the minimum and maximum value).

### 3.5. CARD FISH

The analyzed prokaryotic community was dominated by Eubacteria. The percentage of DAPI counts detected by the mix EUB338I-III probes ranged from 24.09 % to 89.16% (data not-shown). The general pattern describing the proportions of bacterial groups detected by CARD FISH showed the dominance of SAR11, followed by general Alphaproteobacteria, Gammaproteobacteria, *Bacteroidetes*, and finally *Roseobacter*. However, seasonal changes were observed in some groups. In winter, Alphaproteobacteria predominated in a range from 2.24% to 52.23%, while in summer, Gammaproteobacteria predominated with a minimum of 4.52% to a maximum of 57.60%. SAR 11 had the highest counts in spring and fall, ranging from of 5.29% to 53.9%.

Bacteroides dominated in spring at the open sea station, also with higher DAPI counts at the other two stations ranging from 2.32% to 49.92%. We also observed dominance of this group in the deep chlorophyll layer regardless of season (3.97%-30.76%). Roseobacter showed a range from 5.28 % to 31.36 % during the highest values. On the open sea, the highest values occurred in summer, while in coastal sites they were recorded in spring.

### 3.6. 16S sequencing results

SI Table 2. 16S sequencing results from NovaSeq 6000 Illumina, performed at Novogene Europe; Raw PE-original PE reads after sequencing; Raw Tags-tags merged from PE reads; Clean Tags-tags after filtering; Effective Tags-tags after filtering chimera and can be finally used for subsequent analysis; Base(nt)-the number of bases of the Effective Tags; AvgLen -average length of Effective Tags; Q20 and Q30-percentages of bases whose quality value in Effective Tags is greater than 20 (sequencing error rate is less than 1%) and 30 (sequencing error rate is less than 0.1%) respectively; GC (%) - GC content in Effective Tags; Effective (%) - percentage of Effective Tags in Raw PE

| Sample Name | Raw PE | Raw Tags | Clean Tags | Effective Tags | Base(nt) | AvgLen(nt) | Q20 | Q30 | GC% | Effective% |
| --- | --- | --- | --- | --- | --- | --- | --- | --- | --- | --- |
| A.10 | 127,806 | 123,379 | 122,876 | 97,593 | 36,362,167 | 373 | 98.83 | 95.85 | 49.01 | 76.36 |
| A.11 | 120,588 | 109,191 | 108,56 | 80,436 | 29,942,468 | 372 | 98.64 | 95.52 | 51.68 | 66.70 |
| A.12 | 126,637 | 115,292 | 114,728 | 88,624 | 32,976,450 | 372 | 98.67 | 95.51 | 48.62 | 69.98 |
| A.13 | 271,071 | 255,111 | 253,734 | 180,698 | 67,180,145 | 372 | 98.56 | 95.13 | 50.01 | 66.66 |
| A.14 | 138,141 | 133,326 | 132,816 | 102,753 | 38,213,000 | 372 | 98.94 | 96.30 | 50.05 | 74.38 |
| A.15 | 128,109 | 123,484 | 122,961 | 94,61 | 35,199,387 | 372 | 98.90 | 96.18 | 49.83 | 73.85 |
| A.16 | 288,192 | 277,734 | 276,289 | 214,613 | 79,829,336 | 372 | 98.81 | 95.82 | 49.05 | 74.47 |
| A.17 | 130,85 | 126,294 | 125,723 | 93,872 | 34,946,961 | 372 | 98.88 | 96.13 | 49.94 | 71.74 |
| A.18 | 109,975 | 106,99 | 106,512 | 80,142 | 29,868,300 | 373 | 99.00 | 96.45 | 50.43 | 72.87 |
| A.19 | 130,355 | 126,132 | 125,585 | 98,598 | 36,756,702 | 373 | 98.92 | 96.19 | 49.30 | 75.64 |
| A.1 | 281,666 | 269,226 | 267,846 | 215,743 | 80,416,733 | 373 | 98.64 | 95.32 | 48.55 | 76.60 |
| A.20 | 139,714 | 134,19 | 133,523 | 105,447 | 39,367,396 | 373 | 98.79 | 95.84 | 49.78 | 75.47 |
| A.21 | 121,26 | 116,76 | 116,184 | 84,467 | 31,448,583 | 372 | 98.83 | 95.98 | 50.14 | 69.66 |
| A.22 | 137,489 | 132,893 | 132,26 | 99,523 | 37,080,532 | 373 | 98.88 | 96.09 | 49.52 | 72.39 |
| A.23 | 126,797 | 116,358 | 115,728 | 100,969 | 37,614,714 | 373 | 98.69 | 95.64 | 49.91 | 79.63 |
| A.24 | 138,35 | 127,161 | 126,443 | 104,328 | 38,850,241 | 372 | 98.70 | 95.67 | 49.52 | 75.41 |
| A.25 | 277,242 | 259,657 | 257,762 | 225,721 | 84,002,446 | 372 | 98.46 | 94.93 | 49.65 | 81.42 |
| A.26 | 132,031 | 128,014 | 127,526 | 103,852 | 38,609,514 | 372 | 99.02 | 96.48 | 48.97 | 78.66 |
| A.27 | 131,816 | 126,274 | 125,708 | 102,139 | 37,967,139 | 372 | 98.90 | 96.15 | 48.33 | 77.49 |
| A.28 | 72,298 | 70,231 | 69,941 | 59,459 | 22,109,335 | 372 | 99.02 | 96.51 | 48.86 | 82.24 |
| A.29 | 122,351 | 118,088 | 117,544 | 99,018 | 36,821,537 | 372 | 98.95 | 96.27 | 48.53 | 80.93 |
| A.2 | 129,963 | 125,726 | 125,21 | 107,137 | 39,969,814 | 373 | 98.92 | 96.18 | 49.32 | 82.44 |
| A.30 | 126,983 | 123,197 | 122,652 | 102,147 | 38,122,237 | 373 | 98.97 | 96.35 | 49.80 | 80.44 |
| A.31 | 124,564 | 118,778 | 118,266 | 91,139 | 33,935,173 | 372 | 98.83 | 95.97 | 49.81 | 73.17 |
| A.32 | 134,927 | 130,394 | 129,836 | 101,919 | 37,953,722 | 372 | 98.93 | 96.22 | 49.17 | 75.54 |
| A.33 | 123,045 | 116,798 | 116,313 | 90,733 | 33,748,201 | 372 | 98.80 | 95.92 | 48.91 | 73.74 |
| A.34 | 139,52 | 133,932 | 133,398 | 105,969 | 39,492,289 | 373 | 98.87 | 96.07 | 51.02 | 75.95 |
| A.35 | 123,554 | 110,043 | 109,503 | 88,533 | 32,973,681 | 372 | 98.64 | 95.55 | 48.19 | 71.66 |
| A.36 | 121,579 | 107,968 | 107,489 | 87,625 | 32,583,182 | 372 | 98.71 | 95.74 | 48.02 | 72.07 |
| A.37 | 139,758 | 135,449 | 134,744 | 108,595 | 40,524,675 | 373 | 98.83 | 96.03 | 53.47 | 77.70 |
| A.38 | 120,88 | 117,17 | 116,672 | 91,893 | 34,207,647 | 372 | 98.98 | 96.40 | 50.24 | 76.02 |
| A.39 | 128,005 | 123,26 | 122,678 | 92,763 | 34,585,941 | 373 | 98.86 | 96.04 | 49.45 | 72.47 |
| A.3 | 133,759 | 128,521 | 128,018 | 107,943 | 40,247,674 | 373 | 98.84 | 95.95 | 48.68 | 80.70 |
| A.40 | 58,138 | 56,449 | 56,207 | 42,128 | 15,734,994 | 374 | 98.99 | 96.45 | 50.13 | 72.46 |
| A.41 | 129,647 | 125,246 | 124,72 | 94,998 | 35,313,948 | 372 | 98.91 | 96.22 | 50.26 | 73.27 |
| A.42 | 134,802 | 131,008 | 130,421 | 102,562 | 38,189,371 | 372 | 98.98 | 96.42 | 51.32 | 76.08 |
| A.43 | 131,702 | 126,175 | 125,613 | 98,567 | 36,586,974 | 371 | 98.89 | 96.15 | 48.98 | 74.84 |
| A.44 | 126,154 | 121,811 | 121,227 | 100,743 | 37,419,739 | 371 | 98.97 | 96.33 | 49.23 | 79.86 |
| A.46 | 136,416 | 131,474 | 130,917 | 96,71 | 35,938,715 | 372 | 98.95 | 96.30 | 49.81 | 70.89 |
| A.47 | 121,768 | 115,648 | 115,136 | 87,275 | 32,428,272 | 372 | 98.81 | 95.93 | 49.72 | 71.67 |
| A.48 | 130,699 | 117,497 | 116,927 | 93,858 | 34,870,135 | 372 | 98.72 | 95.72 | 50.29 | 71.81 |
| A.49 | 126,164 | 115,744 | 115,009 | 89,312 | 33,330,066 | 373 | 98.71 | 95.71 | 49.64 | 70.79 |
| A.4 | 291,926 | 276,022 | 274,421 | 225,911 | 84,156,026 | 373 | 98.61 | 95.29 | 48.19 | 77.39 |
| A.50 | 137,05 | 124,646 | 123,941 | 89,919 | 33,602,649 | 374 | 98.74 | 95.79 | 49.71 | 65.61 |
| A.51 | 124,287 | 114,576 | 113,981 | 82,427 | 30,702,766 | 372 | 98.81 | 95.95 | 50.17 | 66.32 |
| A.52 | 74,261 | 69,849 | 69,434 | 48,668 | 18,156,495 | 373 | 98.89 | 96.20 | 49.43 | 65.54 |
| A.53 | 133,306 | 122,142 | 121,457 | 93,886 | 35,004,435 | 373 | 98.74 | 95.79 | 49.27 | 70.43 |
| A.54 | 135,849 | 125,341 | 124,595 | 94,793 | 35,348,342 | 373 | 98.75 | 95.82 | 49.33 | 69.78 |
| A.55 | 122,722 | 103,454 | 102,873 | 83 | 30,950,886 | 373 | 98.38 | 94.85 | 49.59 | 67.63 |
| A.56 | 121,243 | 110,986 | 110,338 | 74,839 | 27,873,993 | 372 | 98.76 | 95.85 | 50.01 | 61.73 |
| A.57 | 121,429 | 105,768 | 105,155 | 75,532 | 28,101,444 | 372 | 98.55 | 95.30 | 48.64 | 62.20 |
| A.58 | 138,62 | 123,244 | 122,579 | 88,565 | 32,949,742 | 372 | 98.69 | 95.65 | 48.61 | 63.89 |
| A.59 | 138,506 | 133,818 | 133,221 | 104,335 | 38,857,183 | 372 | 98.87 | 96.10 | 49.72 | 75.33 |
| A.5 | 120,045 | 115,456 | 114,959 | 89,934 | 33,519,068 | 373 | 98.84 | 95.95 | 48.77 | 74.92 |
| A.60 | 130,69 | 126,721 | 126,105 | 100,515 | 37,553,902 | 374 | 98.93 | 96.24 | 50.40 | 76.91 |
| A.61 | 128,692 | 120,13 | 119,5 | 92,238 | 34,324,041 | 372 | 98.84 | 96.06 | 51.06 | 71.67 |
| A.62 | 137,155 | 127,961 | 127,276 | 98,27 | 36,602,905 | 372 | 98.87 | 96.14 | 49.90 | 71.65 |
| A.63 | 135,923 | 125,588 | 124,992 | 94,372 | 35,095,954 | 372 | 98.83 | 96.04 | 49.76 | 69.43 |
| A.64 | 71,832 | 67,85 | 67,477 | 52,927 | 19,720,593 | 373 | 98.87 | 96.17 | 51.43 | 73.68 |
| A.65 | 138,481 | 127,726 | 126,899 | 100,236 | 37,389,597 | 373 | 98.70 | 95.69 | 49.80 | 72.38 |
| A.66 | 127,991 | 118,518 | 117,941 | 92,205 | 34,245,377 | 371 | 98.83 | 96.04 | 50.25 | 72.04 |
| A.67 | 133,756 | 119,815 | 119,212 | 90,748 | 33,736,646 | 372 | 98.71 | 95.74 | 50.06 | 67.85 |
| A.68 | 129,594 | 118,683 | 117,973 | 90,884 | 33,861,765 | 373 | 98.73 | 95.81 | 51.60 | 70.13 |
| A.69 | 126,002 | 109,317 | 108,605 | 88,548 | 33,121,862 | 374 | 98.44 | 95.03 | 51.09 | 70.28 |
| A.6 | 102,51 | 99,491 | 99,092 | 80,617 | 30,016,951 | 372 | 98.95 | 96.28 | 49.05 | 78.64 |
| A.70 | 135,916 | 120,323 | 119,686 | 89,276 | 33,435,505 | 375 | 98.64 | 95.52 | 51.18 | 65.68 |
| A.71 | 128,961 | 124,18 | 123,634 | 96,411 | 35,882,129 | 372 | 98.85 | 96.06 | 51.31 | 74.76 |
| A.72 | 135,716 | 132,14 | 131,507 | 101,8 | 38,048,434 | 374 | 98.93 | 96.23 | 51.36 | 75.01 |
| A.73 | 136,222 | 123,655 | 123,045 | 96,776 | 35,954,438 | 372 | 98.78 | 95.91 | 48.61 | 71.04 |
| A.74 | 136,751 | 122,289 | 121,652 | 98,126 | 36,510,620 | 372 | 98.69 | 95.67 | 48.97 | 71.76 |
| A.75 | 120,365 | 106,069 | 105,551 | 84,771 | 31,571,614 | 372 | 98.60 | 95.39 | 48.25 | 70.43 |
| A.76 | 103,338 | 97,212 | 96,711 | 77,466 | 28,756,781 | 371 | 98.93 | 96.34 | 47.56 | 74.96 |
| A.77 | 126,785 | 110,801 | 110,262 | 89,202 | 33,127,707 | 371 | 98.61 | 95.47 | 47.36 | 70.36 |
| A.78 | 134,292 | 124,374 | 123,693 | 101,657 | 37,797,047 | 372 | 98.81 | 95.99 | 48.05 | 75.70 |
| A.79 | 122,271 | 100,794 | 100,254 | 81,19 | 30,206,040 | 372 | 98.30 | 94.64 | 47.99 | 66.40 |
| A.7 | 134,995 | 130,106 | 129,573 | 108,334 | 40,356,284 | 373 | 98.91 | 96.15 | 48.72 | 80.25 |
| A.80 | 122,853 | 108,176 | 107,45 | 84,002 | 31,308,718 | 373 | 98.52 | 95.20 | 48.83 | 68.38 |
| A.81 | 122,007 | 100,792 | 100,215 | 79,06 | 29,414,678 | 372 | 98.29 | 94.64 | 48.08 | 64.80 |
| A.82 | 127,297 | 106,916 | 106,304 | 82,454 | 30,679,641 | 372 | 98.40 | 94.89 | 48.20 | 64.77 |
| A.83 | 128,997 | 124,15 | 123,548 | 104,173 | 38,833,220 | 373 | 98.83 | 95.96 | 49.07 | 80.76 |
| A.84 | 136,553 | 131,001 | 130,351 | 109,456 | 40,815,144 | 373 | 98.83 | 95.93 | 49.08 | 80.16 |
| A.85 | 120,887 | 110,352 | 109,718 | 90,912 | 33,891,674 | 373 | 98.75 | 95.84 | 49.40 | 75.20 |
| A.86 | 123,43 | 108,618 | 108,017 | 89,42 | 33,328,979 | 373 | 98.58 | 95.43 | 49.92 | 72.45 |
| A.87 | 134,921 | 119,764 | 119,042 | 104,132 | 38,821,524 | 373 | 98.60 | 95.44 | 49.83 | 77.18 |
| A.88 | 114,837 | 108,064 | 107,43 | 87,587 | 32,656,672 | 373 | 98.85 | 96.15 | 50.24 | 76.27 |
| A.89 | 137,281 | 121,736 | 121,062 | 101,095 | 37,678,256 | 373 | 98.58 | 95.41 | 49.91 | 73.64 |
| A.8 | 124,498 | 119,869 | 119,356 | 99,182 | 36,957,340 | 373 | 98.86 | 96.01 | 49.11 | 79.67 |
| A.90 | 139,011 | 128,942 | 128,181 | 111,424 | 41,518,874 | 373 | 98.80 | 95.98 | 49.67 | 80.15 |
| A.9 | 125,086 | 120,699 | 120,173 | 95,871 | 35,687,906 | 372 | 98.89 | 96.08 | 48.60 | 76.64 |

Concerning the number of reads per sample and qualitative description of the microbial community, by far the most predominant bacterial phylum in terms of relative abundances across all samples was Proteobacteria (with main classes Alpha- and Gammaproteobacteria), followed by Cyanobacteria and Bacteroidota, whilst the most abundant archaeal phyla were Thermoplasmatota and Crenarchaeota. The slight qualitative changes in community composition were observed even at the phyla level on a spatiotemporal scale. Bacteroidota relative abundances generally increased in spring and summer and decreased with depth regardless of the area. Quite the opposite, archaeal phyla Crenarchaeota and Thermoplasmatota showed the highest relative abundances in deeper layers and toward the open sea, especially during colder months with maximum relative abundances in December and January. Marinimicrobia (SAR406) were generally present at all stations in deeper layers all year round. Class Actinobacteria was present in higher relative abundances in June and August in the shallow layer up to 50m whilst Kiritimatiellae (phylum Verrucomicrobiota) appeared in higher abundances in May <50m. Differences among community composition were more prominent on genus level shown for the most predominant phyla. Proteobacteria were the most diverse phylum with 35 orders and 171 genera detected. On a finer taxonomic scale, the most relatively abundant proteobacterial classes, Alpha- and Gammaproteobacteria, showed inversed relative abundances in terms of seasonality with Gammaproteobacteria exhibiting the highest values during summer while Alphaproteobacteria dominated in winter months.

Gammaproteobacteria were the most diverse proteobacterial class, represented by the highest number of 49 unique genera and dominated by SAR86 and OM60(NOR5) lineages regardless of the area or season. These two lineages exhibited higher relative abundances in upper layers up to 50m. *Glaciecola* genus appeared in winter months on ST101 and CJ007 stations. *Litoricola*, on the other hand, appeared in higher abundance in spring and summer predominately in surface layers. *Vibrio* was observed in higher relative abundances at all stations in December and January regardless of depth. *Pseudohongiella* appeared in November and December in higher relative abundances regardless of the area. Numerous other genera with individual contributions <3% represented a significant proportion of gammaproteobacterial reads.

The members of Alphaproteobacteria class were represented with 35 unique genera across all samples with the major contributions of omnipresent SAR11 clade Ia and AEGEAN-169 (order Rhodospirillales), followed by SAR11 clade II and to a lesser extent, genus *Ascidiaceihabitans* (family Rhodobacteraceae). SAR11 clade III appeared in the summer in higher abundance at all stations in surface layer. SAR116 clade showed higher relative abundances in May, June and August at all stations. The highest relative abundances of Alphaproteobacteria were generally observed in the euphotic zone (~ 0m). Up to ~25% of alphaproteobacterial reads in all the samples were contributed to genera with a relative abundance of <3%.

Phylum Cyanobacteria was dominated by *Synechococcus* (strain CC9902) at all stations, seasons, and depths. On the other hand, *Prochlorococcus* (strain MIT9313) appeared in higher relative abundances at depths >50m and especially at CJ009 station in January and April. Cyanobium (strain PCC-6307) was omnipresent in all samples while in June an unclassified genus (phylum Cyanobacteria, other taxonomic ranks unknown) was recorded in higher abundances at all stations.

Phylum Bacteroidota consisted of 26 unique genera with major contributors at all stations and depths being uncultured NS4 and NS5 lineages, followed by NS2 and *Fluviicola*. NS9 lineage showed the highest relative abundances at the open sea in December, Januray and February in depths below 50m. Genus *Balneola* appeared in layer above 50m and exhibited the lowest relative abundances in December, January and February at all stations and the highest ones in May, June and August.


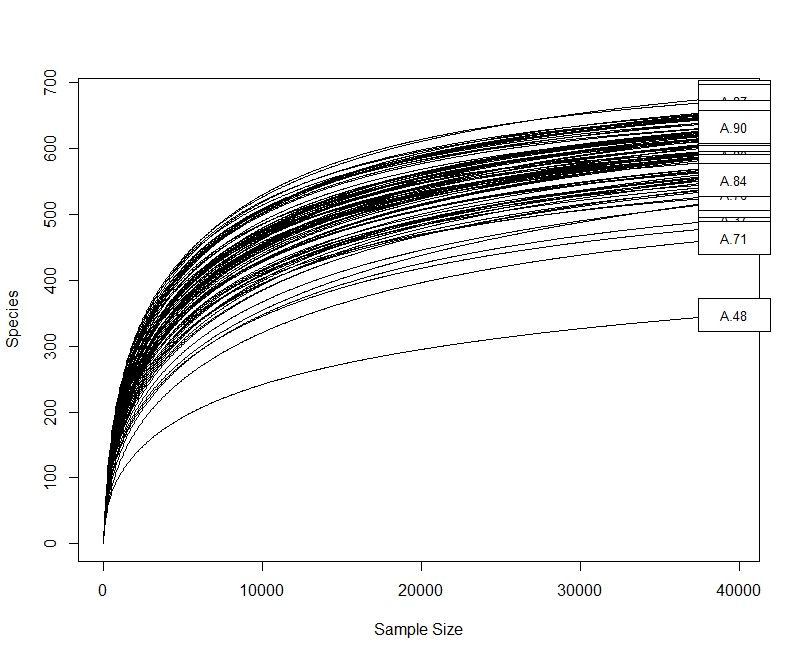


SI Figure 4. Rarefaction curve exhibiting sufficient sequencing depth (Sample Size) for microbial diversity estimates.

SI Table 3. Taxonomic table with taxonomic ranks shown for each OTU based on the annotation with the SSU rRNA database of SILVA138 (Kingdom, Phylum, Class, Order, Family, Genus)

| OTU_1 | Bacteria | Proteobacteria | Alphaproteobacteria | SAR11_clade | Clade_I | Clade_Ia |
| --- | --- | --- | --- | --- | --- | --- |
| OTU_2 | Bacteria | Cyanobacteria | Cyanobacteriia | Synechococcales | Cyanobiaceae | Synechococcus_CC9902 |
| OTU_3 | Archaea | Crenarchaeota | Nitrososphaeria | Nitrosopumilales | Nitrosopumilaceae | Candidatus_Nitrosopumilus |
| OTU_4 | Bacteria | Proteobacteria | Alphaproteobacteria | Rhodobacterales | Rhodobacteraceae | Ascidiaceihabitans |
| OTU_5 | Bacteria | Proteobacteria | Alphaproteobacteria | SAR11_clade | Clade_II | Clade_II |
| OTU_6 | Archaea | Crenarchaeota | Nitrososphaeria | Nitrosopumilales | Nitrosopumilaceae | Candidatus_Nitrosopelagicus |
| OTU_7 | Bacteria | Proteobacteria | Alphaproteobacteria | Sphingomonadales | Sphingomonadaceae | Erythrobacter |
| OTU_8 | Bacteria | Proteobacteria | Alphaproteobacteria | Rhodospirillales | AEGEAN-169_marine_group | AEGEAN-169_marine_group |
| OTU_10 | Bacteria | Bacteroidota | Bacteroidia | Flavobacteriales | Flavobacteriaceae | NS4_marine_group |
| OTU_11 | Archaea | Thermoplasmatota | Thermoplasmata | Marine_Group_II | Marine_Group_II | Marine_Group_II |
| OTU_12 | Archaea | Thermoplasmatota | Thermoplasmata | Marine_Group_II | Marine_Group_II | Marine_Group_II |
| OTU_13 | Bacteria | Bacteroidota | Bacteroidia | Flavobacteriales | Flavobacteriaceae | NS2b_marine_group |
| OTU_14 | Bacteria | Actinobacteriota | Acidimicrobiia | Actinomarinales | Actinomarinaceae | Candidatus_Actinomarina |
| OTU_15 | Archaea | Thermoplasmatota | Thermoplasmata | Thermoplasmata | Thermoplasmata | Marine_Group_III |
| OTU_16 | Bacteria | Proteobacteria | Gammaproteobacteria | SAR86_clade | SAR86_clade | SAR86_clade |
| OTU_17 | Bacteria | Proteobacteria | Alphaproteobacteria | Rhodospirillales | AEGEAN-169_marine_group | AEGEAN-169_marine_group |
| OTU_18 | Bacteria | Proteobacteria | Gammaproteobacteria | Thiomicrospirales | Thioglobaceae | SUP05_cluster |
| OTU_19 | Bacteria | Bacteroidota | Rhodothermia | Balneolales | Balneolaceae | Balneola |
| OTU_20 | Bacteria | Chloroflexi | Dehalococcoidia | SAR202_clade | SAR202_clade | SAR202_clade |
| OTU_21 | Bacteria | Proteobacteria | Gammaproteobacteria | Oceanospirillales | Litoricolaceae | Litoricola |
| OTU_22 | Bacteria | Proteobacteria | Gammaproteobacteria | Vibrionales | Vibrionaceae | Vibrio |
| OTU_23 | Bacteria | Proteobacteria | Gammaproteobacteria | Cellvibrionales | Halieaceae | OM60(NOR5)_clade |
| OTU_24 | Bacteria | Proteobacteria | Alphaproteobacteria | Puniceispirillales | SAR116_clade | SAR116_clade |
| OTU_25 | Bacteria | Proteobacteria | Gammaproteobacteria | SAR86_clade | SAR86_clade | SAR86_clade |
| OTU_26 | Bacteria | Proteobacteria | Gammaproteobacteria | Cellvibrionales | Halieaceae | OM60(NOR5)_clade |
| OTU_27 | Bacteria | Actinobacteriota | Actinobacteria | Micrococcales | Microbacteriaceae | DS001 |
| OTU_28 | Bacteria | Proteobacteria | Alphaproteobacteria | Thalassobaculales | Nisaeaceae | OM75_clade |
| OTU_29 | Bacteria | Bacteroidota | Bacteroidia | Flavobacteriales | Crocinitomicaceae | Fluviicola |
| OTU_30 | Bacteria | Dadabacteria | Dadabacteriia | Dadabacteriales | Dadabacteriales | Dadabacteriales |
| OTU_31 | Bacteria | Proteobacteria | Alphaproteobacteria | SAR11_clade | Clade_IV | Clade_IV |
| OTU_32 | Bacteria | Proteobacteria | Gammaproteobacteria | Oceanospirillales | Pseudohongiellaceae | Pseudohongiella |
| OTU_33 | Bacteria | Bacteroidota | Bacteroidia | Flavobacteriales | Flavobacteriaceae | NS5_marine_group |
| OTU_34 | Bacteria | Verrucomicrobiota | Verrucomicrobiae | Opitutales | Puniceicoccaceae | Lentimonas |
| OTU_35 | Bacteria | Proteobacteria | Gammaproteobacteria | SAR86_clade | SAR86_clade | SAR86_clade |
| OTU_36 | Bacteria | Bacteroidota | Bacteroidia | Flavobacteriales | NS9_marine_group | NS9_marine_group |
| OTU_37 | Bacteria | Cyanobacteria | Cyanobacteriia | Synechococcales | Cyanobiaceae | Cyanobium_PCC-6307 |
| OTU_38 | Bacteria | Marinimicrobia_(SAR406_clade) | Marinimicrobia_(SAR406_clade) | Marinimicrobia_(SAR406_clade) | Marinimicrobia_(SAR406_clade) | Marinimicrobia_(SAR406_clade) |
| OTU_39 | Bacteria | Verrucomicrobiota | Verrucomicrobiae | Opitutales | Puniceicoccaceae | Coraliomargarita |
| OTU_40 | Bacteria | Bacteroidota | Bacteroidia | Flavobacteriales | Flavobacteriaceae | NS4_marine_group |
| OTU_41 | Bacteria | Proteobacteria | Alphaproteobacteria | Rhizobiales | Rhizobiaceae | Cohaesibacter |
| OTU_42 | Bacteria | Proteobacteria | Alphaproteobacteria | Puniceispirillales | SAR116_clade | Unclassified |
| OTU_43 | Bacteria | Proteobacteria | Alphaproteobacteria | Puniceispirillales | SAR116_clade | SAR116_clade |
| OTU_44 | Bacteria | Verrucomicrobiota | Kiritimatiellae | Kiritimatiellales | Kiritimatiellaceae | R76-B128 |
| OTU_45 | Bacteria | Proteobacteria | Gammaproteobacteria | SAR86_clade | SAR86_clade | SAR86_clade |
| OTU_46 | Bacteria | Proteobacteria | Gammaproteobacteria | HOC36 | HOC36 | HOC36 |
| OTU_48 | Bacteria | Proteobacteria | Alphaproteobacteria | Puniceispirillales | SAR116_clade | SAR116_clade |
| OTU_49 | Archaea | Thermoplasmatota | Thermoplasmata | Marine_Group_II | Marine_Group_II | Marine_Group_II |
| OTU_50 | Bacteria | Proteobacteria | Alphaproteobacteria | Rhodobacterales | Rhodobacteraceae | Ascidiaceihabitans |
| OTU_51 | Bacteria | Marinimicrobia_(SAR406_clade) | Marinimicrobia_(SAR406_clade) | Marinimicrobia_(SAR406_clade) | Marinimicrobia_(SAR406_clade) | Marinimicrobia_(SAR406_clade) |
| OTU_52 | Bacteria | Proteobacteria | Alphaproteobacteria | Parvibaculales | OCS116_clade | OCS116_clade |
| OTU_53 | Archaea | Thermoplasmatota | Thermoplasmata | Marine_Group_II | Marine_Group_II | Marine_Group_II |
| OTU_54 | Bacteria | Proteobacteria | Gammaproteobacteria | SAR86_clade | SAR86_clade | SAR86_clade |
| OTU_55 | Bacteria | Verrucomicrobiota | Verrucomicrobiae | Verrucomicrobiales | DEV007 | DEV007 |
| OTU_56 | Bacteria | Verrucomicrobiota | Verrucomicrobiae | Pedosphaerales | Pedosphaeraceae | SCGC_AAA164-E04 |
| OTU_58 | Bacteria | Bacteroidota | Bacteroidia | Flavobacteriales | Flavobacteriaceae | NS5_marine_group |
| OTU_59 | Bacteria | Proteobacteria | Gammaproteobacteria | KI89A_clade | KI89A_clade | KI89A_clade |
| OTU_60 | Bacteria | Proteobacteria | Alphaproteobacteria | SAR11_clade | Clade_III | Clade_III |
| OTU_61 | Archaea | Thermoplasmatota | Thermoplasmata | Marine_Group_II | Marine_Group_II | Marine_Group_II |
| OTU_62 | Bacteria | Proteobacteria | Gammaproteobacteria | Alteromonadales | Pseudoalteromonadaceae | Pseudoalteromonas |
| OTU_63 | Archaea | Thermoplasmatota | Thermoplasmata | Marine_Group_II | Marine_Group_II | Marine_Group_II |
| OTU_64 | Bacteria | Chloroflexi | Dehalococcoidia | SAR202_clade | SAR202_clade | SAR202_clade |
| OTU_65 | Bacteria | Dadabacteria | Dadabacteriia | Dadabacteriales | Dadabacteriales | Dadabacteriales |
| OTU_66 | Bacteria | Verrucomicrobiota | Verrucomicrobiae | Verrucomicrobiales | Rubritaleaceae | Roseibacillus |
| OTU_67 | Bacteria | Proteobacteria | Alphaproteobacteria | Defluviicoccales | Unclassified | Unclassified |
| OTU_68 | Bacteria | Proteobacteria | Gammaproteobacteria | Thiotrichales | Thiotrichaceae | Unclassified |
| OTU_69 | Bacteria | Proteobacteria | Gammaproteobacteria | Oceanospirillales | Alcanivoracaceae1 | Alcanivorax |
| OTU_70 | Bacteria | Proteobacteria | Gammaproteobacteria | Ectothiorhodospirales | Ectothiorhodospiraceae | Unclassified |
| OTU_71 | Bacteria | Bacteroidota | Bacteroidia | Sphingobacteriales | NS11-12_marine_group | NS11-12_marine_group |
| OTU_72 | Bacteria | Proteobacteria | Alphaproteobacteria | Rhodobacterales | Rhodobacteraceae | Unclassified |
| OTU_73 | Bacteria | Bacteroidota | Bacteroidia | Flavobacteriales | Flavobacteriaceae | NS5_marine_group |
| OTU_74 | Bacteria | Bacteroidota | Bacteroidia | Flavobacteriales | Cryomorphaceae | Unclassified |
| OTU_76 | Bacteria | Proteobacteria | Alphaproteobacteria | Parvibaculales | PS1_clade | PS1_clade |
| OTU_77 | Bacteria | Proteobacteria | Alphaproteobacteria | Puniceispirillales | SAR116_clade | SAR116_clade |
| OTU_78 | Bacteria | Proteobacteria | Alphaproteobacteria | Rhodobacterales | Rhodobacteraceae | Unclassified |
| OTU_79 | Bacteria | Proteobacteria | Alphaproteobacteria | Rhodobacterales | Rhodobacteraceae | Unclassified |
| OTU_80 | Bacteria | SAR324_clade(Marine_group_B) | SAR324_clade(Marine_group_B) | SAR324_clade(Marine_group_B) | SAR324_clade(Marine_group_B) | SAR324_clade(Marine_group_B) |
| OTU_81 | Bacteria | Proteobacteria | Gammaproteobacteria | UBA10353_marine_group | UBA10353_marine_group | UBA10353_marine_group |
| OTU_82 | Bacteria | SAR324_clade(Marine_group_B) | SAR324_clade(Marine_group_B) | SAR324_clade(Marine_group_B) | SAR324_clade(Marine_group_B) | SAR324_clade(Marine_group_B) |
| OTU_84 | Bacteria | Proteobacteria | Alphaproteobacteria | Rhodobacterales | Rhodobacteraceae | Unclassified |
| OTU_85 | Bacteria | Proteobacteria | Gammaproteobacteria | Ga0077536 | Ga0077536 | Ga0077536 |
| OTU_86 | Bacteria | Bacteroidota | Bacteroidia | Flavobacteriales | Cryomorphaceae | Unclassified |
| OTU_87 | Bacteria | Marinimicrobia_(SAR406_clade) | Marinimicrobia_(SAR406_clade) | Marinimicrobia_(SAR406_clade) | Marinimicrobia_(SAR406_clade) | Marinimicrobia_(SAR406_clade) |
| OTU_88 | Bacteria | Bacteroidota | Bacteroidia | Flavobacteriales | Flavobacteriaceae | NS4_marine_group |
| OTU_89 | Bacteria | Proteobacteria | Gammaproteobacteria | Alteromonadales | Alteromonadaceae | Glaciecola |
| OTU_90 | Bacteria | Proteobacteria | Alphaproteobacteria | Sphingomonadales | Sphingomonadaceae | Altererythrobacter |
| OTU_91 | Bacteria | Bacteroidota | Bacteroidia | Flavobacteriales | Flavobacteriaceae | NS5_marine_group |
| OTU_92 | Bacteria | Planctomycetota | Planctomycetes | Pirellulales | Pirellulaceae | Rhodopirellula |
| OTU_93 | Bacteria | Planctomycetota | OM190 | OM190 | OM190 | OM190 |
| OTU_94 | Bacteria | Actinobacteriota | Acidimicrobiia | Actinomarinales | Actinomarinaceae | Candidatus_Actinomarina |
| OTU_95 | Bacteria | Marinimicrobia_(SAR406_clade) | Marinimicrobia_(SAR406_clade) | Marinimicrobia_(SAR406_clade) | Marinimicrobia_(SAR406_clade) | Marinimicrobia_(SAR406_clade) |
| OTU_96 | Bacteria | Bacteroidota | Bacteroidia | Flavobacteriales | Flavobacteriaceae | NS5_marine_group |
| OTU_97 | Bacteria | Actinobacteriota | Acidimicrobiia | Microtrichales | Microtrichaceae | Sva0996_marine_group |
| OTU_98 | Bacteria | Proteobacteria | Gammaproteobacteria | Alteromonadales | Marinobacteraceae | Marinobacter |
| OTU_99 | Bacteria | Planctomycetota | Planctomycetes | Pirellulales | Pirellulaceae | Unclassified |
| OTU_100 | Bacteria | Bacteroidota | Bacteroidia | Flavobacteriales | Cryomorphaceae | Unclassified |
| OTU_101 | Bacteria | Proteobacteria | Alphaproteobacteria | SAR11_clade | Clade_IV | Clade_IV |
| OTU_102 | Bacteria | Proteobacteria | Alphaproteobacteria | Rhodobacterales | Rhodobacteraceae | Unclassified |
| OTU_103 | Bacteria | Bacteroidota | Bacteroidia | Cytophagales | Cyclobacteriaceae | Marinoscillum |
| OTU_104 | Bacteria | Proteobacteria | Alphaproteobacteria | Parvibaculales | OCS116_clade | OCS116_clade |
| OTU_105 | Bacteria | Proteobacteria | Gammaproteobacteria | Steroidobacterales | Woeseiaceae | Woeseia |
| OTU_106 | Bacteria | Proteobacteria | Alphaproteobacteria | Unclassified | Unclassified | Unclassified |
| OTU_107 | Bacteria | SAR324_clade(Marine_group_B) | SAR324_clade(Marine_group_B) | SAR324_clade(Marine_group_B) | SAR324_clade(Marine_group_B) | SAR324_clade(Marine_group_B) |
| OTU_108 | Archaea | Thermoplasmatota | Thermoplasmata | Marine_Group_II | Marine_Group_II | Marine_Group_II |
| OTU_109 | Bacteria | Planctomycetota | Pla3_lineage | Pla3_lineage | Pla3_lineage | Pla3_lineage |
| OTU_110 | Bacteria | Proteobacteria | Alphaproteobacteria | Rickettsiales | S25-593 | S25-593 |
| OTU_111 | Bacteria | Actinobacteriota | Acidimicrobiia | Actinomarinales | Actinomarinaceae | Candidatus_Actinomarina |
| OTU_112 | Bacteria | Proteobacteria | Alphaproteobacteria | Parvibaculales | Parvibaculaceae | Unclassified |
| OTU_113 | Bacteria | Bacteroidota | Bacteroidia | Flavobacteriales | Flavobacteriaceae | Formosa |
| OTU_115 | Bacteria | Marinimicrobia_(SAR406_clade) | Marinimicrobia_(SAR406_clade) | Marinimicrobia_(SAR406_clade) | Marinimicrobia_(SAR406_clade) | Marinimicrobia_(SAR406_clade) |
| OTU_116 | Bacteria | Proteobacteria | Gammaproteobacteria | Burkholderiales | Burkholderiaceae | Ralstonia |
| OTU_117 | Bacteria | Actinobacteriota | Actinobacteria | Micrococcales | Microbacteriaceae | Microbacterium |
| OTU_118 | Bacteria | Bacteroidota | Bacteroidia | Flavobacteriales | NS7_marine_group | NS7_marine_group |
| OTU_119 | Bacteria | Proteobacteria | Alphaproteobacteria | Rhizobiales | Stappiaceae | Stappiaceae |
| OTU_120 | Bacteria | Proteobacteria | Gammaproteobacteria | Pseudomonadales | Moraxellaceae | Acinetobacter |
| OTU_121 | Bacteria | Proteobacteria | Alphaproteobacteria | Caulobacterales | Hyphomonadaceae | Oceanicaulis |
| OTU_122 | Archaea | Thermoplasmatota | Thermoplasmata | Marine_Group_II | Marine_Group_II | Marine_Group_II |
| OTU_123 | Bacteria | Verrucomicrobiota | Verrucomicrobiae | Opitutales | Puniceicoccaceae | Coraliomargarita |
| OTU_124 | Bacteria | Verrucomicrobiota | Verrucomicrobiae | Verrucomicrobiales | Rubritaleaceae | Roseibacillus |
| OTU_125 | Bacteria | Planctomycetota | Phycisphaerae | Phycisphaerales | Phycisphaeraceae | SM1A02 |
| OTU_126 | Bacteria | Planctomycetota | OM190 | OM190 | OM190 | OM190 |
| OTU_127 | Bacteria | Actinobacteriota | Acidimicrobiia | Microtrichales | Microtrichaceae | Sva0996_marine_group |
| OTU_128 | Bacteria | Proteobacteria | Gammaproteobacteria | SAR86_clade | SAR86_clade | SAR86_clade |
| OTU_129 | Bacteria | Proteobacteria | Gammaproteobacteria | SAR86_clade | SAR86_clade | SAR86_clade |
| OTU_130 | Bacteria | Proteobacteria | Alphaproteobacteria | Rhizobiales | Beijerinckiaceae | Methylobacterium-Methylorubrum |
| OTU_131 | Bacteria | Proteobacteria | Gammaproteobacteria | Burkholderiales | Methylophilaceae | OM43_clade |
| OTU_132 | Bacteria | Proteobacteria | Gammaproteobacteria | OM182_clade | OM182_clade | OM182_clade |
| OTU_133 | Bacteria | Gemmatimonadota | BD2-11_terrestrial_group | BD2-11_terrestrial_group | BD2-11_terrestrial_group | BD2-11_terrestrial_group |
| OTU_134 | Bacteria | Proteobacteria | Gammaproteobacteria | OM182_clade | OM182_clade | OM182_clade |
| OTU_135 | Bacteria | Verrucomicrobiota | Verrucomicrobiae | Opitutales | Puniceicoccaceae | Pelagicoccus |
| OTU_136 | Bacteria | Proteobacteria | Alphaproteobacteria | Parvibaculales | PS1_clade | PS1_clade |
| OTU_138 | Bacteria | Proteobacteria | Gammaproteobacteria | SAR86_clade | SAR86_clade | SAR86_clade |
| OTU_139 | Bacteria | Bacteroidota | Bacteroidia | Flavobacteriales | NS9_marine_group | NS9_marine_group |
| OTU_140 | Bacteria | Proteobacteria | Gammaproteobacteria | Burkholderiales | Methylophilaceae | OM43_clade |
| OTU_141 | Bacteria | Proteobacteria | Alphaproteobacteria | Caulobacterales | Hyphomonadaceae | Unclassified |
| OTU_142 | Bacteria | Proteobacteria | Gammaproteobacteria | Cellvibrionales | Porticoccaceae | SAR92_clade |
| OTU_143 | Bacteria | Proteobacteria | Alphaproteobacteria | Rhodospirillales | Magnetospiraceae | Unclassified |
| OTU_144 | Bacteria | Bacteroidota | Bacteroidia | Flavobacteriales | Flavobacteriaceae | Unclassified |
| OTU_145 | Bacteria | Verrucomicrobiota | Verrucomicrobiae | Opitutales | Puniceicoccaceae | MB11C04_marine_group |
| OTU_146 | Bacteria | Actinobacteriota | Actinobacteria | Propionibacteriales | Propionibacteriaceae | Cutibacterium |
| OTU_147 | Bacteria | Proteobacteria | Gammaproteobacteria | Cellvibrionales | Halieaceae | Luminiphilus |
| OTU_148 | Bacteria | Proteobacteria | Gammaproteobacteria | Tenderiales | Tenderiaceae | Candidatus_Tenderia |
| OTU_149 | Bacteria | Verrucomicrobiota | Verrucomicrobiae | Verrucomicrobiales | Rubritaleaceae | Persicirhabdus |
| OTU_150 | Bacteria | Bacteroidota | Bacteroidia | Flavobacteriales | Flavobacteriaceae | Unclassified |
| OTU_151 | Bacteria | Chloroflexi | Dehalococcoidia | SAR202_clade | SAR202_clade | SAR202_clade |
| OTU_152 | Bacteria | Bacteroidota | Bacteroidia | Flavobacteriales | Flavobacteriaceae | NS5_marine_group |
| OTU_153 | Bacteria | Proteobacteria | Gammaproteobacteria | Burkholderiales | Nitrosomonadaceae | IS-44 |
| OTU_154 | Archaea | Crenarchaeota | Nitrososphaeria | Nitrosopumilales | Nitrosopumilaceae | Candidatus_Nitrosopelagicus |
| OTU_155 | Bacteria | Chloroflexi | Dehalococcoidia | SAR202_clade | SAR202_clade | SAR202_clade |
| OTU_157 | Bacteria | Bacteroidota | Bacteroidia | Flavobacteriales | Flavobacteriaceae | NS5_marine_group |
| OTU_158 | Bacteria | Actinobacteriota | Acidimicrobiia | Microtrichales | Microtrichaceae | Sva0996_marine_group |
| OTU_159 | Bacteria | Chloroflexi | Dehalococcoidia | SAR202_clade | SAR202_clade | SAR202_clade |
| OTU_160 | Bacteria | Proteobacteria | Alphaproteobacteria | SAR11_clade | Clade_I | Clade_Ib |
| OTU_161 | Bacteria | Verrucomicrobiota | Verrucomicrobiae | Arctic97B-4_marine_group | Arctic97B-4_marine_group | Arctic97B-4_marine_group |
| OTU_162 | Bacteria | Chloroflexi | JG30-KF-CM66 | JG30-KF-CM66 | JG30-KF-CM66 | JG30-KF-CM66 |
| OTU_164 | Bacteria | Proteobacteria | Alphaproteobacteria | Puniceispirillales | SAR116_clade | SAR116_clade |
| OTU_165 | Bacteria | Proteobacteria | Alphaproteobacteria | Unclassified | Unclassified | Unclassified |
| OTU_167 | Bacteria | Bacteroidota | Bacteroidia | Flavobacteriales | NS9_marine_group | NS9_marine_group |
| OTU_168 | Bacteria | Actinobacteriota | Actinobacteria | Bifidobacteriales | Bifidobacteriaceae | Gardnerella |
| OTU_169 | Bacteria | Verrucomicrobiota | Verrucomicrobiae | Arctic97B-4_marine_group | Arctic97B-4_marine_group | Arctic97B-4_marine_group |
| OTU_170 | Bacteria | Verrucomicrobiota | Verrucomicrobiae | Opitutales | Puniceicoccaceae | MB11C04_marine_group |
| OTU_174 | Bacteria | Proteobacteria | Alphaproteobacteria | Rhodospirillales | Magnetospiraceae | Unclassified |
| OTU_175 | Bacteria | Bacteroidota | Rhodothermia | Balneolales | Balneolaceae | Unclassified |
| OTU_176 | Bacteria | Proteobacteria | Alphaproteobacteria | Rhodospirillales | Magnetospiraceae | Unclassified |
| OTU_177 | Bacteria | Planctomycetota | Planctomycetes | Planctomycetales | Gimesiaceae | Unclassified |
| OTU_178 | Bacteria | Planctomycetota | Planctomycetes | Pirellulales | Pirellulaceae | Blastopirellula |
| OTU_179 | Bacteria | Actinobacteriota | Acidimicrobiia | Microtrichales | Microtrichaceae | Sva0996_marine_group |
| OTU_180 | Bacteria | Nitrospinota | Nitrospinia | Nitrospinales | Nitrospinaceae | LS-NOB |
| OTU_181 | Bacteria | Proteobacteria | Gammaproteobacteria | Oceanospirillales | Halomonadaceae | Halomonas |
| OTU_182 | Bacteria | Proteobacteria | Alphaproteobacteria | Defluviicoccales | Unclassified | Unclassified |
| OTU_183 | Bacteria | Bacteroidota | Bacteroidia | Flavobacteriales | Flavobacteriaceae | NS4_marine_group |
| OTU_184 | Bacteria | Proteobacteria | Alphaproteobacteria | Puniceispirillales | SAR116_clade | SAR116_clade |
| OTU_185 | Bacteria | Bacteroidota | Bacteroidia | Flavobacteriales | NS9_marine_group | NS9_marine_group |
| OTU_186 | Archaea | Thermoplasmatota | Thermoplasmata | Marine_Group_II | Marine_Group_II | Marine_Group_II |
| OTU_187 | Bacteria | Proteobacteria | Gammaproteobacteria | Burkholderiales | MWH-UniP1_aquatic_group | MWH-UniP1_aquatic_group |
| OTU_188 | Bacteria | Proteobacteria | Alphaproteobacteria | Caulobacterales | Hyphomonadaceae | Hyphomonas |
| OTU_189 | Bacteria | Proteobacteria | Alphaproteobacteria | SAR11_clade | Clade_II | Clade_II |
| OTU_190 | Bacteria | Bacteroidota | Bacteroidia | Flavobacteriales | NS7_marine_group | NS7_marine_group |
| OTU_191 | Bacteria | Proteobacteria | Gammaproteobacteria | Cellvibrionales | Halieaceae | OM60(NOR5)_clade |
| OTU_192 | Bacteria | Bacteroidota | Bacteroidia | Flavobacteriales | Cryomorphaceae | Unclassified |
| OTU_193 | Bacteria | Marinimicrobia_(SAR406_clade) | Marinimicrobia_(SAR406_clade) | Marinimicrobia_(SAR406_clade) | Marinimicrobia_(SAR406_clade) | Marinimicrobia_(SAR406_clade) |
| OTU_194 | Bacteria | Proteobacteria | Alphaproteobacteria | Rhodobacterales | Rhodobacteraceae | Unclassified |
| OTU_195 | Bacteria | SAR324_clade(Marine_group_B) | SAR324_clade(Marine_group_B) | SAR324_clade(Marine_group_B) | SAR324_clade(Marine_group_B) | SAR324_clade(Marine_group_B) |
| OTU_196 | Bacteria | Proteobacteria | Alphaproteobacteria | Thalassobaculales | Unclassified | Unclassified |
| OTU_197 | Bacteria | Verrucomicrobiota | Verrucomicrobiae | Arctic97B-4_marine_group | Arctic97B-4_marine_group | Arctic97B-4_marine_group |
| OTU_198 | Bacteria | Planctomycetota | Planctomycetes | Pirellulales | Pirellulaceae | Rhodopirellula |
| OTU_199 | Bacteria | Proteobacteria | Gammaproteobacteria | SAR86_clade | SAR86_clade | SAR86_clade |
| OTU_200 | Bacteria | Myxococcota | Polyangia | Nannocystales | Nannocystaceae | Unclassified |
| OTU_201 | Bacteria | Proteobacteria | Alphaproteobacteria | Rhizobiales | Rhizobiaceae | Roseitalea |
| OTU_202 | Bacteria | Proteobacteria | Gammaproteobacteria | KI89A_clade | KI89A_clade | KI89A_clade |
| OTU_203 | Bacteria | Firmicutes | Bacilli | Lactobacillales | Lactobacillaceae | Lactobacillus |
| OTU_204 | Bacteria | Desulfobacterota | Unclassified | Unclassified | Unclassified | Unclassified |
| OTU_205 | Bacteria | Proteobacteria | Alphaproteobacteria | Puniceispirillales | SAR116_clade | SAR116_clade |
| OTU_206 | Bacteria | Planctomycetota | OM190 | OM190 | OM190 | OM190 |
| OTU_208 | Bacteria | Verrucomicrobiota | Verrucomicrobiae | Opitutales | Puniceicoccaceae | Cerasicoccus |
| OTU_209 | Bacteria | Proteobacteria | Alphaproteobacteria | Defluviicoccales | Unclassified | Unclassified |
| OTU_210 | Bacteria | Chloroflexi | Dehalococcoidia | SAR202_clade | SAR202_clade | SAR202_clade |
| OTU_211 | Bacteria | Bacteroidota | Bacteroidia | Flavobacteriales | Flavobacteriaceae | NS5_marine_group |
| OTU_212 | Archaea | Thermoplasmatota | Thermoplasmata | Marine_Group_II | Marine_Group_II | Marine_Group_II |
| OTU_213 | Bacteria | Proteobacteria | Gammaproteobacteria | KI89A_clade | KI89A_clade | KI89A_clade |
| OTU_214 | Bacteria | Bacteroidota | Bacteroidia | Flavobacteriales | Cryomorphaceae | NS10_marine_group |
| OTU_215 | Archaea | Thermoplasmatota | Thermoplasmata | Thermoplasmata | Thermoplasmata | Marine_Group_III |
| OTU_216 | Bacteria | Proteobacteria | Gammaproteobacteria | Oceanospirillales | Saccharospirillaceae | Oceaniserpentilla |
| OTU_217 | Bacteria | Bacteroidota | Bacteroidia | Flavobacteriales | Flavobacteriaceae | NS3a_marine_group |
| OTU_218 | Bacteria | Bacteroidota | Bacteroidia | Chitinophagales | Saprospiraceae | Unclassified |
| OTU_219 | Bacteria | Firmicutes | Bacilli | Lactobacillales | Lactobacillaceae | Lactobacillus |
| OTU_220 | Bacteria | Planctomycetota | Planctomycetes | Pirellulales | Pirellulaceae | Rubripirellula |
| OTU_222 | Bacteria | Proteobacteria | Gammaproteobacteria | KI89A_clade | KI89A_clade | KI89A_clade |
| OTU_223 | Bacteria | Verrucomicrobiota | Verrucomicrobiae | Arctic97B-4_marine_group | Arctic97B-4_marine_group | Arctic97B-4_marine_group |
| OTU_224 | Bacteria | Proteobacteria | Gammaproteobacteria | EPR3968-O8a-Bc78 | EPR3968-O8a-Bc78 | EPR3968-O8a-Bc78 |
| OTU_225 | Bacteria | Verrucomicrobiota | Verrucomicrobiae | Verrucomicrobiales | DEV007 | DEV007 |
| OTU_226 | Bacteria | Proteobacteria | Gammaproteobacteria | Alteromonadales | Alteromonadaceae | Alteromonas |
| OTU_227 | Bacteria | Firmicutes | Clostridia | Clostridia_UCG-014 | Clostridia_UCG-014 | Clostridia_UCG-014 |
| OTU_228 | Bacteria | Bacteroidota | Bacteroidia | Flavobacteriales | NS9_marine_group | NS9_marine_group |
| OTU_229 | Bacteria | Planctomycetota | Planctomycetes | Pirellulales | Pirellulaceae | Blastopirellula |
| OTU_230 | Bacteria | Proteobacteria | Alphaproteobacteria | Kiloniellales | Kiloniellaceae | Unclassified |
| OTU_231 | Bacteria | Bacteroidota | Bacteroidia | Flavobacteriales | NS9_marine_group | NS9_marine_group |
| OTU_232 | Bacteria | Proteobacteria | Alphaproteobacteria | Puniceispirillales | Unclassified | Unclassified |
| OTU_233 | Bacteria | Proteobacteria | Gammaproteobacteria | Alteromonadales | Idiomarinaceae | Idiomarina |
| OTU_234 | Bacteria | Proteobacteria | Gammaproteobacteria | SAR86_clade | SAR86_clade | SAR86_clade |
| OTU_235 | Bacteria | Proteobacteria | Gammaproteobacteria | UBA10353_marine_group | UBA10353_marine_group | UBA10353_marine_group |
| OTU_236 | Bacteria | Proteobacteria | Alphaproteobacteria | SAR11_clade | Unclassified | Unclassified |
| OTU_237 | Bacteria | Bacteroidota | Bacteroidia | Flavobacteriales | Flavobacteriaceae | Unclassified |
| OTU_238 | Bacteria | NB1-j | NB1-j | NB1-j | NB1-j | NB1-j |
| OTU_239 | Bacteria | Proteobacteria | Alphaproteobacteria | Rickettsiales | S25-593 | S25-593 |
| OTU_240 | Archaea | Thermoplasmatota | Thermoplasmata | Thermoplasmata | Thermoplasmata | Marine_Group_III |
| OTU_241 | Bacteria | Bdellovibrionota | Bdellovibrionia | Bdellovibrionales | Bdellovibrionaceae | OM27_clade |
| OTU_242 | Archaea | Thermoplasmatota | Thermoplasmata | Marine_Group_II | Marine_Group_II | Marine_Group_II |
| OTU_243 | Archaea | Crenarchaeota | Nitrososphaeria | Nitrosopumilales | Nitrosopumilaceae | Candidatus_Nitrosopelagicus |
| OTU_244 | Bacteria | Proteobacteria | Unclassified | Unclassified | Unclassified | Unclassified |
| OTU_245 | Bacteria | Actinobacteriota | Acidimicrobiia | Microtrichales | Microtrichaceae | Sva0996_marine_group |
| OTU_246 | Bacteria | Chloroflexi | Dehalococcoidia | SAR202_clade | SAR202_clade | SAR202_clade |
| OTU_247 | Bacteria | Proteobacteria | Gammaproteobacteria | Alteromonadales | Marinobacteraceae | Marinobacter |
| OTU_248 | Bacteria | NB1-j | NB1-j | NB1-j | NB1-j | NB1-j |
| OTU_249 | Bacteria | Proteobacteria | Gammaproteobacteria | Cellvibrionales | Porticoccaceae | SAR92_clade |
| OTU_250 | Bacteria | Proteobacteria | Gammaproteobacteria | HOC36 | HOC36 | HOC36 |
| OTU_251 | Bacteria | Proteobacteria | Alphaproteobacteria | SAR11_clade | Unclassified | Unclassified |
| OTU_252 | Bacteria | Marinimicrobia_(SAR406_clade) | Marinimicrobia_(SAR406_clade) | Marinimicrobia_(SAR406_clade) | Marinimicrobia_(SAR406_clade) | Marinimicrobia_(SAR406_clade) |
| OTU_254 | Bacteria | Bacteroidota | Bacteroidia | Flavobacteriales | Flavobacteriaceae | Unclassified |
| OTU_255 | Bacteria | Proteobacteria | Alphaproteobacteria | SAR11_clade | Clade_III | Clade_III |
| OTU_256 | Bacteria | Verrucomicrobiota | Verrucomicrobiae | Pedosphaerales | Pedosphaeraceae | SCGC_AAA164-E04 |
| OTU_257 | Bacteria | Proteobacteria | Alphaproteobacteria | Defluviicoccales | Unclassified | Unclassified |
| OTU_258 | Bacteria | Proteobacteria | Alphaproteobacteria | Rhodospirillales | Magnetospiraceae | Unclassified |
| OTU_259 | Bacteria | Actinobacteriota | Actinobacteria | PeM15 | PeM15 | PeM15 |
| OTU_260 | Bacteria | Marinimicrobia_(SAR406_clade) | Marinimicrobia_(SAR406_clade) | Marinimicrobia_(SAR406_clade) | Marinimicrobia_(SAR406_clade) | Marinimicrobia_(SAR406_clade) |
| OTU_261 | Bacteria | Bacteroidota | Bacteroidia | Sphingobacteriales | NS11-12_marine_group | NS11-12_marine_group |
| OTU_262 | Bacteria | Planctomycetota | Phycisphaerae | Phycisphaerales | Phycisphaeraceae | CL500-3 |
| OTU_263 | Bacteria | Bacteroidota | Bacteroidia | Flavobacteriales | Cryomorphaceae | Unclassified |
| OTU_264 | Bacteria | Proteobacteria | Alphaproteobacteria | Rickettsiales | S25-593 | S25-593 |
| OTU_265 | Bacteria | Bacteroidota | Bacteroidia | Flavobacteriales | Flavobacteriaceae | Ulvibacter |
| OTU_266 | Bacteria | Marinimicrobia_(SAR406_clade) | Marinimicrobia_(SAR406_clade) | Marinimicrobia_(SAR406_clade) | Marinimicrobia_(SAR406_clade) | Marinimicrobia_(SAR406_clade) |
| OTU_267 | Bacteria | Proteobacteria | Alphaproteobacteria | Puniceispirillales | Unclassified | Unclassified |
| OTU_268 | Bacteria | Proteobacteria | Gammaproteobacteria | Oceanospirillales | Nitrincolaceae | Marinobacterium |
| OTU_269 | Bacteria | Proteobacteria | Gammaproteobacteria | Burkholderiales | MWH-UniP1_aquatic_group | MWH-UniP1_aquatic_group |
| OTU_271 | Bacteria | Marinimicrobia_(SAR406_clade) | Marinimicrobia_(SAR406_clade) | Marinimicrobia_(SAR406_clade) | Marinimicrobia_(SAR406_clade) | Marinimicrobia_(SAR406_clade) |
| OTU_272 | Bacteria | Proteobacteria | Gammaproteobacteria | Nitrosococcales | Nitrosococcaceae | Cm1-21 |
| OTU_273 | Bacteria | Proteobacteria | Gammaproteobacteria | EPR3968-O8a-Bc78 | EPR3968-O8a-Bc78 | EPR3968-O8a-Bc78 |
| OTU_274 | Bacteria | Actinobacteriota | Acidimicrobiia | Microtrichales | Microtrichaceae | Sva0996_marine_group |
| OTU_275 | Bacteria | Bacteroidota | Bacteroidia | Flavobacteriales | NS9_marine_group | NS9_marine_group |
| OTU_276 | Bacteria | Marinimicrobia_(SAR406_clade) | Marinimicrobia_(SAR406_clade) | Marinimicrobia_(SAR406_clade) | Marinimicrobia_(SAR406_clade) | Marinimicrobia_(SAR406_clade) |
| OTU_277 | Bacteria | Planctomycetota | Phycisphaerae | Phycisphaerales | Phycisphaeraceae | CL500-3 |
| OTU_278 | Bacteria | Firmicutes | Clostridia | Lachnospirales | Lachnospiraceae | Lachnospiraceae_UCG-006 |
| OTU_279 | Bacteria | Bacteroidota | Bacteroidia | Bacteroidales | Marinifilaceae | Odoribacter |
| OTU_280 | Bacteria | Chloroflexi | Dehalococcoidia | SAR202_clade | SAR202_clade | SAR202_clade |
| OTU_281 | Bacteria | Firmicutes | Clostridia | Lachnospirales | Lachnospiraceae | Lachnospiraceae_NK4A136_group |
| OTU_282 | Bacteria | Chloroflexi | JG30-KF-CM66 | JG30-KF-CM66 | JG30-KF-CM66 | JG30-KF-CM66 |
| OTU_283 | Bacteria | Bacteroidota | Bacteroidia | Flavobacteriales | Blattabacteriaceae | Candidatus_Sulcia |
| OTU_284 | Bacteria | Bacteroidota | Bacteroidia | Flavobacteriales | NS9_marine_group | NS9_marine_group |
| OTU_285 | Bacteria | Verrucomicrobiota | Kiritimatiellae | Kiritimatiellales | Kiritimatiellaceae | R76-B128 |
| OTU_286 | Bacteria | Planctomycetota | Planctomycetes | Pirellulales | Pirellulaceae | Unclassified |
| OTU_287 | Bacteria | Bacteroidota | Bacteroidia | Cytophagales | Cyclobacteriaceae | Marinoscillum |
| OTU_288 | Bacteria | Proteobacteria | Alphaproteobacteria | SAR11_clade | Unclassified | Unclassified |
| OTU_289 | Bacteria | Bacteroidota | Bacteroidia | Flavobacteriales | Crocinitomicaceae | Fluviicola |
| OTU_291 | Bacteria | Proteobacteria | Gammaproteobacteria | KI89A_clade | KI89A_clade | KI89A_clade |
| OTU_292 | Bacteria | Planctomycetota | OM190 | OM190 | OM190 | OM190 |
| OTU_293 | Bacteria | Verrucomicrobiota | Verrucomicrobiae | Pedosphaerales | Pedosphaeraceae | SCGC_AAA164-E04 |
| OTU_294 | Bacteria | Bacteroidota | Bacteroidia | Flavobacteriales | Crocinitomicaceae | Salinirepens |
| OTU_295 | Bacteria | Proteobacteria | Alphaproteobacteria | Rhizobiales | Stappiaceae | Labrenzia |
| OTU_296 | Bacteria | PAUC34f | PAUC34f | PAUC34f | PAUC34f | PAUC34f |
| OTU_297 | Bacteria | Proteobacteria | Gammaproteobacteria | Ectothiorhodospirales | Ectothiorhodospiraceae | Unclassified |
| OTU_298 | Bacteria | Verrucomicrobiota | Verrucomicrobiae | Opitutales | Puniceicoccaceae | Pelagicoccus |
| OTU_299 | Bacteria | Proteobacteria | Alphaproteobacteria | Rhodospirillales | Magnetospiraceae | Unclassified |
| OTU_300 | Bacteria | Firmicutes | Bacilli | Staphylococcales | Staphylococcaceae | Staphylococcus |
| OTU_301 | Bacteria | Proteobacteria | Alphaproteobacteria | Rhodobacterales | Rhodobacteraceae | Pseudophaeobacter |
| OTU_303 | Bacteria | Planctomycetota | Phycisphaerae | Phycisphaerales | Phycisphaeraceae | Unclassified |
| OTU_306 | Bacteria | Bacteroidota | Bacteroidia | Flavobacteriales | NS9_marine_group | NS9_marine_group |
| OTU_307 | Bacteria | Proteobacteria | Alphaproteobacteria | Rhodospirillales | Magnetospiraceae | Magnetospira |
| OTU_308 | Bacteria | Desulfobacterota | Desulfuromonadia | Bradymonadales | Bradymonadales | Bradymonadales |
| OTU_309 | Bacteria | Proteobacteria | Gammaproteobacteria | SS1-B-07-19 | SS1-B-07-19 | SS1-B-07-19 |
| OTU_310 | Bacteria | Bacteroidota | Bacteroidia | Flavobacteriales | Flavobacteriaceae | Flavobacterium |
| OTU_312 | Bacteria | Bacteroidota | Bacteroidia | Flavobacteriales | NS9_marine_group | NS9_marine_group |
| OTU_313 | Bacteria | Firmicutes | Clostridia | Oscillospirales | Ruminococcaceae | Eubacterium_siraeum_group |
| OTU_314 | Archaea | Thermoplasmatota | Thermoplasmata | Marine_Group_II | Marine_Group_II | Marine_Group_II |
| OTU_316 | Bacteria | Unclassified | Unclassified | Unclassified | Unclassified | Unclassified |
| OTU_317 | Bacteria | Marinimicrobia_(SAR406_clade) | Marinimicrobia_(SAR406_clade) | Marinimicrobia_(SAR406_clade) | Marinimicrobia_(SAR406_clade) | Marinimicrobia_(SAR406_clade) |
| OTU_318 | Bacteria | Planctomycetota | OM190 | OM190 | OM190 | OM190 |
| OTU_319 | Bacteria | Firmicutes | Bacilli | Bacillales | Bacillaceae | Bacillus |
| OTU_321 | Bacteria | Proteobacteria | Alphaproteobacteria | Caulobacterales | Hyphomonadaceae | Maricaulis |
| OTU_322 | Bacteria | Planctomycetota | Phycisphaerae | Phycisphaerales | Phycisphaeraceae | CL500-3 |
| OTU_324 | Archaea | Crenarchaeota | Nitrososphaeria | Nitrosopumilales | Nitrosopumilaceae | Nitrosopumilaceae |
| OTU_325 | Bacteria | Planctomycetota | Planctomycetes | Pirellulales | Pirellulaceae | Unclassified |
| OTU_326 | Bacteria | Bacteroidota | Bacteroidia | Flavobacteriales | Flavobacteriaceae | Muricauda |
| OTU_327 | Bacteria | PAUC34f | PAUC34f | PAUC34f | PAUC34f | PAUC34f |
| OTU_328 | Bacteria | Proteobacteria | Alphaproteobacteria | Rickettsiales | S25-593 | S25-593 |
| OTU_329 | Bacteria | Verrucomicrobiota | Verrucomicrobiae | Opitutales | Puniceicoccaceae | MB11C04_marine_group |
| OTU_330 | Bacteria | Marinimicrobia_(SAR406_clade) | Marinimicrobia_(SAR406_clade) | Marinimicrobia_(SAR406_clade) | Marinimicrobia_(SAR406_clade) | Marinimicrobia_(SAR406_clade) |
| OTU_331 | Bacteria | Cyanobacteria | Cyanobacteriia | Unclassified | Unclassified | Unclassified |
| OTU_332 | Bacteria | Proteobacteria | Gammaproteobacteria | Oceanospirillales | Oleiphilaceae | Oleiphilus |
| OTU_333 | Bacteria | Bacteroidota | Bacteroidia | Flavobacteriales | NS9_marine_group | NS9_marine_group |
| OTU_334 | Bacteria | Bacteroidota | Bacteroidia | Flavobacteriales | Flavobacteriaceae | Unclassified |
| OTU_335 | Bacteria | Myxococcota | Myxococcia | Myxococcales | Myxococcaceae | P3OB-42 |
| OTU_336 | Bacteria | Proteobacteria | Gammaproteobacteria | Enterobacterales | Yersiniaceae | Serratia |
| OTU_337 | Bacteria | Verrucomicrobiota | Verrucomicrobiae | Opitutales | Puniceicoccaceae | Unclassified |
| OTU_338 | Bacteria | Proteobacteria | Alphaproteobacteria | Defluviicoccales | Unclassified | Unclassified |
| OTU_339 | Bacteria | Bacteroidota | Bacteroidia | Flavobacteriales | Flavobacteriaceae | Flavobacteriaceae |
| OTU_340 | Bacteria | Bacteroidota | Bacteroidia | Flavobacteriales | Cryomorphaceae | Unclassified |
| OTU_341 | Bacteria | Proteobacteria | Gammaproteobacteria | OM182_clade | OM182_clade | OM182_clade |
| OTU_343 | Bacteria | Proteobacteria | Gammaproteobacteria | Unclassified | Unclassified | Unclassified |
| OTU_345 | Bacteria | Proteobacteria | Alphaproteobacteria | Unclassified | Unclassified | Unclassified |
| OTU_346 | Bacteria | Planctomycetota | Planctomycetes | Planctomycetales | Rubinisphaeraceae | Unclassified |
| OTU_348 | Bacteria | Actinobacteriota | Acidimicrobiia | Microtrichales | Microtrichaceae | Sva0996_marine_group |
| OTU_349 | Bacteria | Bacteroidota | Bacteroidia | Flavobacteriales | Flavobacteriaceae | NS5_marine_group |
| OTU_350 | Bacteria | Marinimicrobia_(SAR406_clade) | Marinimicrobia_(SAR406_clade) | Marinimicrobia_(SAR406_clade) | Marinimicrobia_(SAR406_clade) | Marinimicrobia_(SAR406_clade) |
| OTU_352 | Bacteria | Proteobacteria | Gammaproteobacteria | Oceanospirillales | Nitrincolaceae | Unclassified |
| OTU_353 | Bacteria | Unclassified | Unclassified | Unclassified | Unclassified | Unclassified |
| OTU_354 | Bacteria | Proteobacteria | Gammaproteobacteria | SAR86_clade | SAR86_clade | SAR86_clade |
| OTU_355 | Bacteria | Chloroflexi | Dehalococcoidia | SAR202_clade | SAR202_clade | SAR202_clade |
| OTU_356 | Bacteria | Proteobacteria | Gammaproteobacteria | Ga0077536 | Ga0077536 | Ga0077536 |
| OTU_357 | Bacteria | Chloroflexi | Dehalococcoidia | SAR202_clade | SAR202_clade | SAR202_clade |
| OTU_358 | Bacteria | Actinobacteriota | Acidimicrobiia | Microtrichales | Ilumatobacteraceae | Unclassified |
| OTU_359 | Bacteria | Proteobacteria | Alphaproteobacteria | Unclassified | Unclassified | Unclassified |
| OTU_360 | Bacteria | Proteobacteria | Alphaproteobacteria | Rhodobacterales | Rhodobacteraceae | Unclassified |
| OTU_361 | Bacteria | Proteobacteria | Alphaproteobacteria | Rickettsiales | S25-593 | S25-593 |
| OTU_362 | Bacteria | Nitrospinota | Nitrospinia | Nitrospinales | Nitrospinaceae | LS-NOB |
| OTU_363 | Bacteria | Desulfobacterota | Desulfuromonadia | PB19 | PB19 | PB19 |
| OTU_364 | Bacteria | Planctomycetota | Planctomycetes | Pirellulales | Pirellulaceae | Unclassified |
| OTU_365 | Bacteria | NB1-j | NB1-j | NB1-j | NB1-j | NB1-j |
| OTU_366 | Bacteria | Bacteroidota | Bacteroidia | Flavobacteriales | Flavobacteriaceae | NS4_marine_group |
| OTU_367 | Bacteria | Proteobacteria | Gammaproteobacteria | Oceanospirillales | Pseudohongiellaceae | Pseudohongiella |
| OTU_368 | Bacteria | Proteobacteria | Gammaproteobacteria | SAR86_clade | SAR86_clade | SAR86_clade |
| OTU_370 | Bacteria | Planctomycetota | Planctomycetes | Planctomycetales | Unclassified | Unclassified |
| OTU_371 | Bacteria | Proteobacteria | Gammaproteobacteria | EPR3968-O8a-Bc78 | EPR3968-O8a-Bc78 | EPR3968-O8a-Bc78 |
| OTU_372 | Bacteria | Cyanobacteria | Cyanobacteriia | Synechococcales | Cyanobiaceae | Prochlorococcus_MIT9313 |
| OTU_373 | Bacteria | Proteobacteria | Gammaproteobacteria | Oceanospirillales | Alcanivoracaceae1 | Alcanivorax |
| OTU_374 | Bacteria | Proteobacteria | Alphaproteobacteria | SAR11_clade | Clade_II | Clade_II |
| OTU_375 | Bacteria | Bdellovibrionota | Bdellovibrionia | Bdellovibrionales | Bdellovibrionaceae | OM27_clade |
| OTU_376 | Archaea | Thermoplasmatota | Thermoplasmata | Marine_Group_II | Marine_Group_II | Marine_Group_II |
| OTU_377 | Bacteria | Proteobacteria | Alphaproteobacteria | Unclassified | Unclassified | Unclassified |
| OTU_378 | Archaea | Crenarchaeota | Nitrososphaeria | Nitrosopumilales | Nitrosopumilaceae | Candidatus_Nitrosopumilus |
| OTU_380 | Bacteria | Verrucomicrobiota | Verrucomicrobiae | Opitutales | Puniceicoccaceae | A714019 |
| OTU_382 | Bacteria | Desulfobacterota | Desulfuromonadia | PB19 | PB19 | PB19 |
| OTU_383 | Bacteria | Unclassified | Unclassified | Unclassified | Unclassified | Unclassified |
| OTU_385 | Bacteria | Firmicutes | Clostridia | Oscillospirales | Ruminococcaceae | Faecalibacterium |
| OTU_386 | Bacteria | Myxococcota | Polyangia | Polyangiales | Sandaracinaceae | Unclassified |
| OTU_387 | Bacteria | Bacteroidota | Bacteroidia | Flavobacteriales | Flavobacteriaceae | Unclassified |
| OTU_388 | Bacteria | Unclassified | Unclassified | Unclassified | Unclassified | Unclassified |
| OTU_389 | Bacteria | Planctomycetota | Planctomycetes | Planctomycetales | Rubinisphaeraceae | Unclassified |
| OTU_390 | Bacteria | Proteobacteria | Gammaproteobacteria | SAR86_clade | SAR86_clade | SAR86_clade |
| OTU_391 | Bacteria | Proteobacteria | Alphaproteobacteria | Rhodobacterales | Rhodobacteraceae | Thalassobius |
| OTU_392 | Bacteria | Chloroflexi | Dehalococcoidia | SAR202_clade | SAR202_clade | SAR202_clade |
| OTU_394 | Bacteria | Marinimicrobia_(SAR406_clade) | Marinimicrobia_(SAR406_clade) | Marinimicrobia_(SAR406_clade) | Marinimicrobia_(SAR406_clade) | Marinimicrobia_(SAR406_clade) |
| OTU_395 | Bacteria | Proteobacteria | Gammaproteobacteria | HgCo23 | HgCo23 | HgCo23 |
| OTU_396 | Bacteria | Desulfobacterota | Desulfuromonadia | PB19 | PB19 | PB19 |
| OTU_399 | Bacteria | Proteobacteria | Alphaproteobacteria | Puniceispirillales | SAR116_clade | Candidatus_Puniceispirillum |
| OTU_400 | Bacteria | Proteobacteria | Gammaproteobacteria | Burkholderiales | Comamonadaceae | RS62_marine_group |
| OTU_403 | Bacteria | Proteobacteria | Alphaproteobacteria | Micavibrionales | Micavibrionaceae | Unclassified |
| OTU_404 | Bacteria | Proteobacteria | Gammaproteobacteria | Cellvibrionales | Spongiibacteraceae | BD1-7_clade |
| OTU_405 | Bacteria | Planctomycetota | Planctomycetes | Planctomycetales | Gimesiaceae | Unclassified |
| OTU_406 | Bacteria | Proteobacteria | Gammaproteobacteria | Ga0077536 | Ga0077536 | Ga0077536 |
| OTU_408 | Bacteria | Marinimicrobia_(SAR406_clade) | Marinimicrobia_(SAR406_clade) | Marinimicrobia_(SAR406_clade) | Marinimicrobia_(SAR406_clade) | Marinimicrobia_(SAR406_clade) |
| OTU_411 | Bacteria | Proteobacteria | Alphaproteobacteria | Unclassified | Unclassified | Unclassified |
| OTU_412 | Archaea | Thermoplasmatota | Thermoplasmata | Marine_Group_II | Marine_Group_II | Marine_Group_II |
| OTU_413 | Bacteria | Bacteroidota | Bacteroidia | Flavobacteriales | Flavobacteriaceae | NS5_marine_group |
| OTU_414 | Bacteria | Gemmatimonadota | BD2-11_terrestrial_group | BD2-11_terrestrial_group | BD2-11_terrestrial_group | BD2-11_terrestrial_group |
| OTU_415 | Bacteria | Myxococcota | Polyangia | Blfdi19 | Blfdi19 | Blfdi19 |
| OTU_417 | Archaea | Thermoplasmatota | Thermoplasmata | Marine_Group_II | Marine_Group_II | Marine_Group_II |
| OTU_419 | Bacteria | Chloroflexi | JG30-KF-CM66 | JG30-KF-CM66 | JG30-KF-CM66 | JG30-KF-CM66 |
| OTU_420 | Bacteria | Bacteroidota | Bacteroidia | Flavobacteriales | NS7_marine_group | NS7_marine_group |
| OTU_422 | Bacteria | Proteobacteria | Gammaproteobacteria | UBA10353_marine_group | UBA10353_marine_group | UBA10353_marine_group |
| OTU_423 | Bacteria | Planctomycetota | Phycisphaerae | Phycisphaerales | Phycisphaeraceae | JL-ETNP-F27 |
| OTU_424 | Bacteria | Bdellovibrionota | Bdellovibrionia | Bdellovibrionales | Bdellovibrionaceae | OM27_clade |
| OTU_425 | Bacteria | Proteobacteria | Gammaproteobacteria | Cellvibrionales | Porticoccaceae | Porticoccus |
| OTU_426 | Bacteria | Bacteroidota | Bacteroidia | Flavobacteriales | Cryomorphaceae | Vicingus |
| OTU_429 | Bacteria | Proteobacteria | Gammaproteobacteria | Steroidobacterales | Woeseiaceae | Woeseia |
| OTU_433 | Bacteria | Proteobacteria | Alphaproteobacteria | Thalassobaculales | Thalassobaculaceae | Thalassobaculum |
| OTU_434 | Bacteria | Proteobacteria | Alphaproteobacteria | Unclassified | Unclassified | Unclassified |
| OTU_435 | Bacteria | Hydrogenedentes | Hydrogenedentia | Hydrogenedentiales | Hydrogenedensaceae | Hydrogenedensaceae |
| OTU_436 | Bacteria | Acidobacteriota | Vicinamibacteria | Vicinamibacterales | Unclassified | Unclassified |
| OTU_438 | Bacteria | Actinobacteriota | Actinobacteria | Frankiales | Sporichthyaceae | hgcI_clade |
| OTU_440 | Bacteria | Bdellovibrionota | Bdellovibrionia | Bdellovibrionales | Bdellovibrionaceae | OM27_clade |
| OTU_441 | Bacteria | Proteobacteria | Alphaproteobacteria | Rickettsiales | S25-593 | S25-593 |
| OTU_443 | Bacteria | Bdellovibrionota | Bdellovibrionia | Bdellovibrionales | Bdellovibrionaceae | OM27_clade |
| OTU_444 | Bacteria | Bdellovibrionota | Bdellovibrionia | Bdellovibrionales | Bdellovibrionaceae | OM27_clade |
| OTU_445 | Bacteria | Proteobacteria | Alphaproteobacteria | Rhodospirillales | AEGEAN-169_marine_group | AEGEAN-169_marine_group |
| OTU_447 | Bacteria | Proteobacteria | Alphaproteobacteria | Puniceispirillales | SAR116_clade | SAR116_clade |
| OTU_448 | Bacteria | Proteobacteria | Alphaproteobacteria | Caulobacterales | Hyphomonadaceae | Henriciella |
| OTU_449 | Bacteria | Bacteroidota | Bacteroidia | Bacteroidales | Bacteroidaceae | Bacteroides |
| OTU_450 | Bacteria | Proteobacteria | Gammaproteobacteria | Burkholderiales | Comamonadaceae | Unclassified |
| OTU_452 | Bacteria | NB1-j | NB1-j | NB1-j | NB1-j | NB1-j |
| OTU_453 | Bacteria | Bacteroidota | Bacteroidia | Flavobacteriales | Unclassified | Unclassified |
| OTU_455 | Bacteria | Bacteroidota | Bacteroidia | Cytophagales | Cyclobacteriaceae | Unclassified |
| OTU_458 | Bacteria | Planctomycetota | OM190 | OM190 | OM190 | OM190 |
| OTU_460 | Bacteria | Proteobacteria | Gammaproteobacteria | Nitrosococcales | Nitrosococcaceae | Cm1-21 |
| OTU_461 | Bacteria | Proteobacteria | Gammaproteobacteria | Burkholderiales | Unclassified | Unclassified |
| OTU_463 | Bacteria | Proteobacteria | Gammaproteobacteria | Coxiellales | Coxiellaceae | Coxiella |
| OTU_467 | Bacteria | Planctomycetota | OM190 | OM190 | OM190 | OM190 |
| OTU_469 | Bacteria | Proteobacteria | Alphaproteobacteria | Caulobacterales | Hyphomonadaceae | Oceanicaulis |
| OTU_476 | Bacteria | Bdellovibrionota | Bdellovibrionia | Bacteriovoracales | Bacteriovoracaceae | Unclassified |
| OTU_482 | Bacteria | Proteobacteria | Alphaproteobacteria | Unclassified | Unclassified | Unclassified |
| OTU_484 | Bacteria | Bacteroidota | Bacteroidia | Chitinophagales | Saprospiraceae | Aureispira |
| OTU_488 | Bacteria | Marinimicrobia_(SAR406_clade) | Marinimicrobia_(SAR406_clade) | Marinimicrobia_(SAR406_clade) | Marinimicrobia_(SAR406_clade) | Marinimicrobia_(SAR406_clade) |
| OTU_489 | Bacteria | Chloroflexi | Dehalococcoidia | SAR202_clade | SAR202_clade | SAR202_clade |
| OTU_490 | Bacteria | Proteobacteria | Gammaproteobacteria | Oceanospirillales | Litoricolaceae | Litoricola |
| OTU_491 | Bacteria | Proteobacteria | Gammaproteobacteria | Coxiellales | Coxiellaceae | Coxiella |
| OTU_493 | Bacteria | Chloroflexi | Dehalococcoidia | SAR202_clade | SAR202_clade | SAR202_clade |
| OTU_496 | Bacteria | Proteobacteria | Gammaproteobacteria | Unclassified | Unclassified | Unclassified |
| OTU_498 | Bacteria | Proteobacteria | Gammaproteobacteria | KI89A_clade | KI89A_clade | KI89A_clade |
| OTU_500 | Bacteria | Proteobacteria | Alphaproteobacteria | Rhizobiales | Hyphomicrobiaceae | Filomicrobium |
| OTU_502 | Bacteria | Bacteroidota | Bacteroidia | Flavobacteriales | Flavobacteriaceae | Polaribacter |
| OTU_504 | Bacteria | Proteobacteria | Alphaproteobacteria | Caulobacterales | Parvularculaceae | Unclassified |
| OTU_506 | Bacteria | Planctomycetota | Phycisphaerae | Phycisphaerales | Phycisphaeraceae | FS140-16B-02_marine_group |
| OTU_507 | Bacteria | Desulfobacterota | Desulfuromonadia | PB19 | PB19 | PB19 |
| OTU_510 | Bacteria | Planctomycetota | OM190 | OM190 | OM190 | OM190 |
| OTU_515 | Bacteria | Unclassified | Unclassified | Unclassified | Unclassified | Unclassified |
| OTU_516 | Bacteria | Bdellovibrionota | Oligoflexia | Oligoflexales | Unclassified | Unclassified |
| OTU_517 | Bacteria | Marinimicrobia_(SAR406_clade) | Marinimicrobia_(SAR406_clade) | Marinimicrobia_(SAR406_clade) | Marinimicrobia_(SAR406_clade) | Marinimicrobia_(SAR406_clade) |
| OTU_520 | Bacteria | Proteobacteria | Alphaproteobacteria | AT-s3-44 | AT-s3-44 | AT-s3-44 |
| OTU_521 | Bacteria | Bdellovibrionota | Oligoflexia | Oligoflexales | Unclassified | Unclassified |
| OTU_523 | Archaea | Thermoplasmatota | Thermoplasmata | Marine_Group_II | Marine_Group_II | Marine_Group_II |
| OTU_524 | Bacteria | Proteobacteria | Gammaproteobacteria | Cellvibrionales | Halieaceae | Unclassified |
| OTU_527 | Bacteria | Proteobacteria | Gammaproteobacteria | Pseudomonadales | Moraxellaceae | Psychrobacter |
| OTU_528 | Bacteria | Marinimicrobia_(SAR406_clade) | Marinimicrobia_(SAR406_clade) | Marinimicrobia_(SAR406_clade) | Marinimicrobia_(SAR406_clade) | Marinimicrobia_(SAR406_clade) |
| OTU_529 | Bacteria | Bdellovibrionota | Bdellovibrionia | Bdellovibrionales | Bdellovibrionaceae | OM27_clade |
| OTU_531 | Bacteria | Desulfobacterota | Desulfuromonadia | Bradymonadales | Bradymonadales | Bradymonadales |
| OTU_534 | Bacteria | Cyanobacteria | Cyanobacteriia | Unclassified | Unclassified | Unclassified |
| OTU_535 | Bacteria | Proteobacteria | Unclassified | Unclassified | Unclassified | Unclassified |
| OTU_536 | Bacteria | Proteobacteria | Alphaproteobacteria | SAR11_clade | Clade_III | Clade_III |
| OTU_537 | Bacteria | Unclassified | Unclassified | Unclassified | Unclassified | Unclassified |
| OTU_539 | Bacteria | Proteobacteria | Alphaproteobacteria | Sphingomonadales | Sphingomonadaceae | Sphingobium |
| OTU_541 | Bacteria | Proteobacteria | Gammaproteobacteria | Oceanospirillales | Saccharospirillaceae | Oleibacter |
| OTU_549 | Bacteria | Proteobacteria | Gammaproteobacteria | Piscirickettsiales | Piscirickettsiaceae | Candidatus_Endoecteinascidia |
| OTU_557 | Bacteria | Proteobacteria | Alphaproteobacteria | SAR11_clade | Clade_I | Clade_Ia |
| OTU_571 | Archaea | Thermoplasmatota | Thermoplasmata | Marine_Group_II | Marine_Group_II | Marine_Group_II |
| OTU_580 | Bacteria | Verrucomicrobiota | Verrucomicrobiae | Arctic97B-4_marine_group | Arctic97B-4_marine_group | Arctic97B-4_marine_group |
| OTU_581 | Bacteria | Bacteroidota | Bacteroidia | Flavobacteriales | Flavobacteriaceae | Unclassified |
| OTU_584 | Bacteria | Planctomycetota | Planctomycetes | Planctomycetales | Rubinisphaeraceae | Unclassified |
| OTU_586 | Bacteria | Proteobacteria | Alphaproteobacteria | Rhodobacterales | Rhodobacteraceae | Unclassified |
| OTU_587 | Bacteria | Proteobacteria | Gammaproteobacteria | Salinisphaerales | Algiphilaceae | Algiphilus |
| OTU_589 | Bacteria | Actinobacteriota | Acidimicrobiia | Actinomarinales | Actinomarinaceae | Candidatus_Actinomarina |
| OTU_590 | Bacteria | Marinimicrobia_(SAR406_clade) | Marinimicrobia_(SAR406_clade) | Marinimicrobia_(SAR406_clade) | Marinimicrobia_(SAR406_clade) | Marinimicrobia_(SAR406_clade) |
| OTU_593 | Bacteria | Actinobacteriota | Actinobacteria | Micrococcales | Microbacteriaceae | Candidatus_Aquiluna |
| OTU_594 | Bacteria | Cyanobacteria | Cyanobacteriia | Synechococcales | Cyanobiaceae | Synechococcus_CC9902 |
| OTU_598 | Bacteria | Bdellovibrionota | Oligoflexia | Oligoflexales | Unclassified | Unclassified |
| OTU_604 | Bacteria | Proteobacteria | Gammaproteobacteria | Cellvibrionales | Porticoccaceae | C1-B045 |
| OTU_609 | Bacteria | Proteobacteria | Alphaproteobacteria | Caulobacterales | Hyphomonadaceae | Maricaulis |
| OTU_613 | Bacteria | Proteobacteria | Gammaproteobacteria | Pseudomonadales | Pseudomonadaceae | Pseudomonas |
| OTU_640 | Archaea | Crenarchaeota | Nitrososphaeria | Nitrosopumilales | Nitrosopumilaceae | Nitrosopumilaceae |
| OTU_642 | Bacteria | Proteobacteria | Alphaproteobacteria | SAR11_clade | Clade_IV | Clade_IV |
| OTU_643 | Archaea | Thermoplasmatota | Thermoplasmata | Marine_Group_II | Marine_Group_II | Marine_Group_II |
| OTU_648 | Bacteria | Planctomycetota | Planctomycetes | Pirellulales | Pirellulaceae | Unclassified |
| OTU_657 | Bacteria | Unclassified | Unclassified | Unclassified | Unclassified | Unclassified |
| OTU_663 | Bacteria | Bacteroidota | Rhodothermia | Balneolales | Balneolaceae | Balneola |
| OTU_667 | Bacteria | Proteobacteria | Gammaproteobacteria | Unclassified | Unclassified | Unclassified |
| OTU_674 | Bacteria | Proteobacteria | Gammaproteobacteria | Cellvibrionales | Spongiibacteraceae | Spongiibacter |
| OTU_676 | Bacteria | Cyanobacteria | Cyanobacteriia | Unclassified | Unclassified | Unclassified |
| OTU_681 | Bacteria | Proteobacteria | Gammaproteobacteria | OM182_clade | OM182_clade | OM182_clade |
| OTU_687 | Bacteria | Proteobacteria | Alphaproteobacteria | Unclassified | Unclassified | Unclassified |
| OTU_689 | Archaea | Crenarchaeota | Nitrososphaeria | Nitrosopumilales | Nitrosopumilaceae | Candidatus_Nitrosopumilus |
| OTU_695 | Bacteria | Bacteroidota | Bacteroidia | Flavobacteriales | Flavobacteriaceae | NS5_marine_group |
| OTU_696 | Bacteria | Bacteroidota | Bacteroidia | Flavobacteriales | Flavobacteriaceae | NS5_marine_group |
| OTU_698 | Bacteria | Proteobacteria | Alphaproteobacteria | SAR11_clade | Unclassified | Unclassified |
| OTU_709 | Bacteria | Bacteroidota | Bacteroidia | Chitinophagales | Saprospiraceae | Lewinella |
| OTU_711 | Bacteria | Unclassified | Unclassified | Unclassified | Unclassified | Unclassified |
| OTU_714 | Bacteria | Proteobacteria | Alphaproteobacteria | Rhodobacterales | Rhodobacteraceae | Unclassified |
| OTU_718 | Bacteria | Proteobacteria | Alphaproteobacteria | Puniceispirillales | SAR116_clade | SAR116_clade |
| OTU_720 | Bacteria | Planctomycetota | OM190 | OM190 | OM190 | OM190 |
| OTU_721 | Bacteria | Gemmatimonadota | BD2-11_terrestrial_group | BD2-11_terrestrial_group | BD2-11_terrestrial_group | BD2-11_terrestrial_group |
| OTU_724 | Bacteria | Margulisbacteria | Margulisbacteria | Margulisbacteria | Margulisbacteria | Margulisbacteria |
| OTU_732 | Bacteria | Proteobacteria | Gammaproteobacteria | Coxiellales | Coxiellaceae | Coxiella |
| OTU_740 | Bacteria | Proteobacteria | Alphaproteobacteria | SAR11_clade | Unclassified | Unclassified |
| OTU_753 | Bacteria | Verrucomicrobiota | Verrucomicrobiae | Unclassified | Unclassified | Unclassified |
| OTU_758 | Bacteria | Bacteroidota | Bacteroidia | Chitinophagales | Saprospiraceae | Unclassified |
| OTU_771 | Bacteria | Proteobacteria | Gammaproteobacteria | SAR86_clade | SAR86_clade | SAR86_clade |
| OTU_772 | Archaea | Crenarchaeota | Nitrososphaeria | Nitrosopumilales | Nitrosopumilaceae | Candidatus_Nitrosopelagicus |
| OTU_777 | Bacteria | Proteobacteria | Gammaproteobacteria | Alteromonadales | Alteromonadaceae | Aestuariibacter |
| OTU_779 | Bacteria | Proteobacteria | Alphaproteobacteria | Defluviicoccales | Unclassified | Unclassified |
| OTU_785 | Bacteria | NB1-j | NB1-j | NB1-j | NB1-j | NB1-j |
| OTU_796 | Bacteria | Unclassified | Unclassified | Unclassified | Unclassified | Unclassified |
| OTU_825 | Bacteria | Bacteroidota | Bacteroidia | Flavobacteriales | Unclassified | Unclassified |
| OTU_850 | Bacteria | Proteobacteria | Alphaproteobacteria | SAR11_clade | Clade_I | Clade_Ia |
| OTU_904 | Archaea | Crenarchaeota | Nitrososphaeria | Nitrosopumilales | Nitrosopumilaceae | Nitrosopumilaceae |
| OTU_934 | Archaea | Thermoplasmatota | Thermoplasmata | Marine_Group_II | Marine_Group_II | Marine_Group_II |
| OTU_937 | Bacteria | Proteobacteria | Alphaproteobacteria | Rhodospirillales | AEGEAN-169_marine_group | AEGEAN-169_marine_group |
| OTU_945 | Bacteria | Chloroflexi | Dehalococcoidia | SAR202_clade | SAR202_clade | SAR202_clade |
| OTU_985 | Archaea | Thermoplasmatota | Thermoplasmata | Marine_Group_II | Marine_Group_II | Marine_Group_II |
| OTU_1006 | Bacteria | Marinimicrobia_(SAR406_clade) | Marinimicrobia_(SAR406_clade) | Marinimicrobia_(SAR406_clade) | Marinimicrobia_(SAR406_clade) | Marinimicrobia_(SAR406_clade) |
| OTU_1008 | Bacteria | Proteobacteria | Gammaproteobacteria | Oceanospirillales | Nitrincolaceae | Unclassified |
| OTU_1011 | Bacteria | Verrucomicrobiota | Verrucomicrobiae | Arctic97B-4_marine_group | Arctic97B-4_marine_group | Arctic97B-4_marine_group |
| OTU_1042 | Bacteria | Proteobacteria | Alphaproteobacteria | SAR11_clade | Clade_II | Clade_II |
| OTU_1047 | Bacteria | Verrucomicrobiota | Verrucomicrobiae | Opitutales | Puniceicoccaceae | MB11C04_marine_group |
| OTU_1051 | Bacteria | Proteobacteria | Alphaproteobacteria | Rhodobacterales | Rhodobacteraceae | Ascidiaceihabitans |
| OTU_1067 | Bacteria | Actinobacteriota | Acidimicrobiia | Actinomarinales | Actinomarinaceae | Candidatus_Actinomarina |
| OTU_1095 | Bacteria | Bacteroidota | Bacteroidia | Flavobacteriales | Flavobacteriaceae | NS5_marine_group |
| OTU_1100 | Bacteria | Bacteroidota | Bacteroidia | Flavobacteriales | Flavobacteriaceae | NS4_marine_group |
| OTU_1102 | Bacteria | Proteobacteria | Alphaproteobacteria | Rhodobacterales | Rhodobacteraceae | Unclassified |
| OTU_1116 | Bacteria | Proteobacteria | Gammaproteobacteria | SAR86_clade | SAR86_clade | SAR86_clade |
| OTU_1120 | Bacteria | Proteobacteria | Gammaproteobacteria | Cellvibrionales | Halieaceae | OM60(NOR5)_clade |
| OTU_1140 | Bacteria | Marinimicrobia_(SAR406_clade) | Marinimicrobia_(SAR406_clade) | Marinimicrobia_(SAR406_clade) | Marinimicrobia_(SAR406_clade) | Marinimicrobia_(SAR406_clade) |
| OTU_1142 | Bacteria | Firmicutes | Bacilli | Bacillales | Bacillaceae | Bacillus |
| OTU_1244 | Bacteria | Bacteroidota | Bacteroidia | Flavobacteriales | Flavobacteriaceae | NS5_marine_group |
| OTU_1265 | Bacteria | Proteobacteria | Alphaproteobacteria | Rhodospirillales | AEGEAN-169_marine_group | AEGEAN-169_marine_group |
| OTU_1287 | Bacteria | Proteobacteria | Alphaproteobacteria | SAR11_clade | Unclassified | Unclassified |
| OTU_1309 | Bacteria | Proteobacteria | Gammaproteobacteria | Cellvibrionales | Halieaceae | Parahaliea |
| OTU_1320 | Bacteria | Proteobacteria | Alphaproteobacteria | SAR11_clade | Clade_I | Unclassified |
| OTU_1334 | Bacteria | Bacteroidota | Bacteroidia | Cytophagales | Cyclobacteriaceae | Marinoscillum |
| OTU_1359 | Bacteria | Proteobacteria | Alphaproteobacteria | SAR11_clade | Unclassified | Unclassified |
| OTU_1371 | Bacteria | Proteobacteria | Alphaproteobacteria | Rhodobacterales | Rhodobacteraceae | Unclassified |
| OTU_1377 | Archaea | Thermoplasmatota | Thermoplasmata | Marine_Group_II | Marine_Group_II | Marine_Group_II |
| OTU_1386 | Archaea | Thermoplasmatota | Thermoplasmata | Thermoplasmata | Thermoplasmata | Marine_Group_III |
| OTU_1390 | Archaea | Thermoplasmatota | Thermoplasmata | Marine_Group_II | Marine_Group_II | Marine_Group_II |
| OTU_1398 | Bacteria | Proteobacteria | Alphaproteobacteria | Rhodobacterales | Rhodobacteraceae | Unclassified |
| OTU_1410 | Bacteria | Proteobacteria | Alphaproteobacteria | Rhodospirillales | AEGEAN-169_marine_group | AEGEAN-169_marine_group |
| OTU_1415 | Bacteria | Proteobacteria | Alphaproteobacteria | Rhodobacterales | Rhodobacteraceae | Unclassified |
| OTU_1445 | Bacteria | Bacteroidota | Bacteroidia | Flavobacteriales | Flavobacteriaceae | NS4_marine_group |
| OTU_1462 | Bacteria | Proteobacteria | Alphaproteobacteria | Rhodospirillales | Magnetospiraceae | Unclassified |
| OTU_1465 | Bacteria | Bacteroidota | Bacteroidia | Flavobacteriales | NS7_marine_group | NS7_marine_group |
| OTU_1482 | Archaea | Crenarchaeota | Nitrososphaeria | Nitrosopumilales | Nitrosopumilaceae | Nitrosopumilaceae |
| OTU_1502 | Bacteria | Marinimicrobia_(SAR406_clade) | Marinimicrobia_(SAR406_clade) | Marinimicrobia_(SAR406_clade) | Marinimicrobia_(SAR406_clade) | Marinimicrobia_(SAR406_clade) |
| OTU_1514 | Bacteria | Gemmatimonadota | BD2-11_terrestrial_group | BD2-11_terrestrial_group | BD2-11_terrestrial_group | BD2-11_terrestrial_group |
| OTU_1539 | Bacteria | Proteobacteria | Alphaproteobacteria | Rhodospirillales | AEGEAN-169_marine_group | AEGEAN-169_marine_group |
| OTU_1560 | Bacteria | Proteobacteria | Gammaproteobacteria | SAR86_clade | SAR86_clade | SAR86_clade |
| OTU_1573 | Bacteria | Proteobacteria | Alphaproteobacteria | SAR11_clade | Unclassified | Unclassified |
| OTU_1581 | Bacteria | Bacteroidota | Rhodothermia | Balneolales | Balneolaceae | Balneola |
| OTU_1606 | Bacteria | Bacteroidota | Bacteroidia | Flavobacteriales | Flavobacteriaceae | Unclassified |
| OTU_1619 | Bacteria | Bacteroidota | Bacteroidia | Flavobacteriales | Flavobacteriaceae | NS5_marine_group |
| OTU_1643 | Bacteria | Proteobacteria | Alphaproteobacteria | Caulobacterales | Hyphomonadaceae | Unclassified |
| OTU_1646 | Bacteria | Cyanobacteria | Cyanobacteriia | Synechococcales | Cyanobiaceae | Synechococcus_CC9902 |
| OTU_1651 | Archaea | Crenarchaeota | Nitrososphaeria | Nitrosopumilales | Nitrosopumilaceae | Candidatus_Nitrosopelagicus |
| OTU_1671 | Bacteria | Bacteroidota | Bacteroidia | Flavobacteriales | Flavobacteriaceae | NS4_marine_group |
| OTU_1673 | Bacteria | Proteobacteria | Gammaproteobacteria | Cellvibrionales | Halieaceae | OM60(NOR5)_clade |
| OTU_1674 | Bacteria | Verrucomicrobiota | Verrucomicrobiae | Opitutales | Puniceicoccaceae | MB11C04_marine_group |
| OTU_1678 | Bacteria | Bacteroidota | Rhodothermia | Balneolales | Balneolaceae | Balneola |
| OTU_1709 | Bacteria | Bacteroidota | Bacteroidia | Flavobacteriales | Flavobacteriaceae | NS2b_marine_group |
| OTU_1710 | Bacteria | Proteobacteria | Alphaproteobacteria | Rhodobacterales | Rhodobacteraceae | Unclassified |
| OTU_1715 | Bacteria | Proteobacteria | Alphaproteobacteria | Puniceispirillales | SAR116_clade | SAR116_clade |
| OTU_1723 | Bacteria | Proteobacteria | Gammaproteobacteria | Alteromonadales | Pseudoalteromonadaceae | Pseudoalteromonas |
| OTU_1761 | Bacteria | Bacteroidota | Bacteroidia | Flavobacteriales | Flavobacteriaceae | NS5_marine_group |
| OTU_1789 | Bacteria | Actinobacteriota | Acidimicrobiia | Actinomarinales | Actinomarinaceae | Candidatus_Actinomarina |
| OTU_1791 | Bacteria | Cyanobacteria | Cyanobacteriia | Synechococcales | Cyanobiaceae | Synechococcus_CC9902 |
| OTU_1831 | Bacteria | Proteobacteria | Gammaproteobacteria | SAR86_clade | SAR86_clade | SAR86_clade |
| OTU_1837 | Bacteria | Actinobacteriota | Acidimicrobiia | Microtrichales | Microtrichaceae | Sva0996_marine_group |
| OTU_1840 | Bacteria | Proteobacteria | Alphaproteobacteria | Rhodobacterales | Rhodobacteraceae | Unclassified |
| OTU_1847 | Bacteria | Verrucomicrobiota | Verrucomicrobiae | Opitutales | Puniceicoccaceae | MB11C04_marine_group |
| OTU_1849 | Bacteria | Marinimicrobia_(SAR406_clade) | Marinimicrobia_(SAR406_clade) | Marinimicrobia_(SAR406_clade) | Marinimicrobia_(SAR406_clade) | Marinimicrobia_(SAR406_clade) |
| OTU_1861 | Bacteria | Proteobacteria | Alphaproteobacteria | Rhodospirillales | Magnetospiraceae | Unclassified |
| OTU_1882 | Bacteria | Proteobacteria | Alphaproteobacteria | Rhodobacterales | Rhodobacteraceae | Unclassified |
| OTU_1905 | Bacteria | Proteobacteria | Alphaproteobacteria | Rhodospirillales | AEGEAN-169_marine_group | AEGEAN-169_marine_group |
| OTU_1919 | Bacteria | Proteobacteria | Alphaproteobacteria | Rhodobacterales | Rhodobacteraceae | Marivivens |
| OTU_1930 | Bacteria | Proteobacteria | Alphaproteobacteria | Defluviicoccales | Unclassified | Unclassified |
| OTU_1948 | Bacteria | Proteobacteria | Alphaproteobacteria | Rickettsiales | S25-593 | S25-593 |
| OTU_2022 | Bacteria | Proteobacteria | Alphaproteobacteria | SAR11_clade | Unclassified | Unclassified |
| OTU_2026 | Bacteria | Proteobacteria | Alphaproteobacteria | SAR11_clade | Clade_I | Clade_Ib |
| OTU_2037 | Bacteria | Proteobacteria | Gammaproteobacteria | OM182_clade | OM182_clade | OM182_clade |
| OTU_2046 | Bacteria | Proteobacteria | Gammaproteobacteria | Oceanospirillales | Litoricolaceae | Litoricola |
| OTU_2073 | Bacteria | Proteobacteria | Alphaproteobacteria | SAR11_clade | Unclassified | Unclassified |
| OTU_2087 | Bacteria | Proteobacteria | Alphaproteobacteria | SAR11_clade | Clade_II | Clade_II |
| OTU_2105 | Bacteria | Proteobacteria | Gammaproteobacteria | Cellvibrionales | Porticoccaceae | SAR92_clade |
| OTU_2119 | Archaea | Thermoplasmatota | Thermoplasmata | Marine_Group_II | Marine_Group_II | Marine_Group_II |
| OTU_2149 | Bacteria | Bacteroidota | Bacteroidia | Flavobacteriales | Cryomorphaceae | NS10_marine_group |
| OTU_2159 | Bacteria | Actinobacteriota | Acidimicrobiia | Actinomarinales | Actinomarinaceae | Candidatus_Actinomarina |
| OTU_2169 | Bacteria | Bacteroidota | Bacteroidia | Flavobacteriales | Flavobacteriaceae | NS4_marine_group |
| OTU_2182 | Bacteria | Proteobacteria | Alphaproteobacteria | Rhodobacterales | Rhodobacteraceae | Unclassified |
| OTU_2233 | Bacteria | Proteobacteria | Gammaproteobacteria | SAR86_clade | SAR86_clade | SAR86_clade |
| OTU_2235 | Bacteria | Verrucomicrobiota | Verrucomicrobiae | Opitutales | Puniceicoccaceae | Unclassified |
| OTU_2244 | Bacteria | Cyanobacteria | Cyanobacteriia | Synechococcales | Cyanobiaceae | Synechococcus_CC9902 |
| OTU_2260 | Bacteria | Proteobacteria | Alphaproteobacteria | Rhodospirillales | AEGEAN-169_marine_group | AEGEAN-169_marine_group |
| OTU_2263 | Bacteria | Proteobacteria | Gammaproteobacteria | SAR86_clade | SAR86_clade | SAR86_clade |
| OTU_2273 | Bacteria | Bacteroidota | Bacteroidia | Flavobacteriales | Flavobacteriaceae | NS5_marine_group |
| OTU_2275 | Bacteria | Proteobacteria | Alphaproteobacteria | SAR11_clade | Unclassified | Unclassified |
| OTU_2323 | Bacteria | Proteobacteria | Gammaproteobacteria | Oceanospirillales | Litoricolaceae | Litoricola |
| OTU_2348 | Bacteria | Chloroflexi | Dehalococcoidia | SAR202_clade | SAR202_clade | SAR202_clade |
| OTU_2355 | Archaea | Thermoplasmatota | Thermoplasmata | Marine_Group_II | Marine_Group_II | Marine_Group_II |
| OTU_2373 | Bacteria | Proteobacteria | Alphaproteobacteria | SAR11_clade | Clade_II | Clade_II |
| OTU_2384 | Bacteria | Proteobacteria | Alphaproteobacteria | SAR11_clade | Clade_II | Clade_II |
| OTU_2400 | Bacteria | Proteobacteria | Alphaproteobacteria | Rhizobiales | Stappiaceae | Stappiaceae |
| OTU_2408 | Bacteria | Proteobacteria | Alphaproteobacteria | SAR11_clade | Clade_I | Clade_Ib |
| OTU_2421 | Archaea | Thermoplasmatota | Thermoplasmata | Thermoplasmata | Thermoplasmata | Marine_Group_III |
| OTU_2426 | Bacteria | Proteobacteria | Alphaproteobacteria | Rickettsiales | S25-593 | S25-593 |
| OTU_2429 | Bacteria | SAR324_clade(Marine_group_B) | SAR324_clade(Marine_group_B) | SAR324_clade(Marine_group_B) | SAR324_clade(Marine_group_B) | SAR324_clade(Marine_group_B) |
| OTU_2442 | Bacteria | Proteobacteria | Gammaproteobacteria | SAR86_clade | SAR86_clade | SAR86_clade |
| OTU_2453 | Bacteria | Bdellovibrionota | Bdellovibrionia | Bdellovibrionales | Bdellovibrionaceae | OM27_clade |
| OTU_2464 | Bacteria | Proteobacteria | Alphaproteobacteria | Rhodospirillales | AEGEAN-169_marine_group | AEGEAN-169_marine_group |
| OTU_2471 | Bacteria | Bacteroidota | Bacteroidia | Flavobacteriales | Flavobacteriaceae | NS5_marine_group |
| OTU_2478 | Bacteria | Proteobacteria | Alphaproteobacteria | Rhodobacterales | Rhodobacteraceae | Planktomarina |
| OTU_2531 | Bacteria | Bacteroidota | Bacteroidia | Flavobacteriales | NS9_marine_group | NS9_marine_group |
| OTU_2536 | Bacteria | Proteobacteria | Alphaproteobacteria | SAR11_clade | Clade_I | Unclassified |
| OTU_2540 | Bacteria | Proteobacteria | Gammaproteobacteria | Oceanospirillales | Litoricolaceae | Litoricola |
| OTU_2582 | Bacteria | Verrucomicrobiota | Verrucomicrobiae | Opitutales | Puniceicoccaceae | MB11C04_marine_group |
| OTU_2599 | Bacteria | Proteobacteria | Alphaproteobacteria | SAR11_clade | Clade_I | Unclassified |
| OTU_2602 | Bacteria | Proteobacteria | Alphaproteobacteria | SAR11_clade | Clade_I | Unclassified |
| OTU_2603 | Bacteria | Proteobacteria | Alphaproteobacteria | Puniceispirillales | SAR116_clade | Unclassified |
| OTU_2611 | Bacteria | Proteobacteria | Alphaproteobacteria | Puniceispirillales | SAR116_clade | SAR116_clade |
| OTU_2623 | Bacteria | Verrucomicrobiota | Verrucomicrobiae | Verrucomicrobiales | DEV007 | DEV007 |
| OTU_2625 | Bacteria | Proteobacteria | Alphaproteobacteria | Rhodobacterales | Rhodobacteraceae | Unclassified |
| OTU_2635 | Bacteria | Proteobacteria | Gammaproteobacteria | Alteromonadales | Pseudoalteromonadaceae | Pseudoalteromonas |
| OTU_2667 | Bacteria | Proteobacteria | Gammaproteobacteria | Burkholderiales | MWH-UniP1_aquatic_group | MWH-UniP1_aquatic_group |
| OTU_2684 | Bacteria | Bacteroidota | Bacteroidia | Flavobacteriales | Flavobacteriaceae | NS4_marine_group |
| OTU_2686 | Bacteria | Cyanobacteria | Cyanobacteriia | Synechococcales | Cyanobiaceae | Unclassified |
| OTU_2731 | Bacteria | Planctomycetota | Planctomycetes | Pirellulales | Pirellulaceae | Unclassified |
| OTU_2742 | Bacteria | Actinobacteriota | Acidimicrobiia | Actinomarinales | Actinomarinaceae | Candidatus_Actinomarina |
| OTU_2752 | Bacteria | Planctomycetota | Planctomycetes | Pirellulales | Pirellulaceae | Rhodopirellula |
| OTU_2764 | Bacteria | Bacteroidota | Bacteroidia | Flavobacteriales | Flavobacteriaceae | NS2b_marine_group |
| OTU_2772 | Bacteria | Proteobacteria | Alphaproteobacteria | SAR11_clade | Clade_I | Unclassified |
| OTU_2805 | Bacteria | Cyanobacteria | Cyanobacteriia | Synechococcales | Cyanobiaceae | Prochlorococcus_MIT9313 |
| OTU_2820 | Bacteria | Bacteroidota | Bacteroidia | Flavobacteriales | Flavobacteriaceae | NS5_marine_group |
| OTU_2883 | Bacteria | Proteobacteria | Alphaproteobacteria | SAR11_clade | Clade_II | Clade_II |
| OTU_2893 | Bacteria | Proteobacteria | Alphaproteobacteria | SAR11_clade | Unclassified | Unclassified |
| OTU_2898 | Bacteria | Proteobacteria | Gammaproteobacteria | SAR86_clade | SAR86_clade | SAR86_clade |
| OTU_2948 | Bacteria | Proteobacteria | Alphaproteobacteria | Rickettsiales | S25-593 | S25-593 |
| OTU_2956 | Bacteria | Bacteroidota | Bacteroidia | Flavobacteriales | NS7_marine_group | NS7_marine_group |
| OTU_2961 | Bacteria | Cyanobacteria | Cyanobacteriia | Synechococcales | Cyanobiaceae | Prochlorococcus_MIT9313 |
| OTU_2966 | Bacteria | Bacteroidota | Bacteroidia | Flavobacteriales | Flavobacteriaceae | NS4_marine_group |
| OTU_2979 | Bacteria | Cyanobacteria | Cyanobacteriia | Synechococcales | Cyanobiaceae | Prochlorococcus_MIT9313 |
| OTU_2986 | Bacteria | Proteobacteria | Alphaproteobacteria | Puniceispirillales | SAR116_clade | SAR116_clade |
| OTU_3002 | Bacteria | Proteobacteria | Alphaproteobacteria | Rhodospirillales | AEGEAN-169_marine_group | AEGEAN-169_marine_group |
| OTU_3007 | Bacteria | Actinobacteriota | Acidimicrobiia | Microtrichales | Microtrichaceae | Sva0996_marine_group |
| OTU_3034 | Bacteria | Bacteroidota | Bacteroidia | Cytophagales | Cyclobacteriaceae | Marinoscillum |
| OTU_3047 | Bacteria | Firmicutes | Bacilli | Lactobacillales | Lactobacillaceae | Lactobacillus |
| OTU_3056 | Bacteria | Proteobacteria | Gammaproteobacteria | SAR86_clade | SAR86_clade | SAR86_clade |
| OTU_3061 | Bacteria | Planctomycetota | Planctomycetes | Pirellulales | Pirellulaceae | Rubripirellula |
| OTU_3077 | Bacteria | Proteobacteria | Alphaproteobacteria | Unclassified | Unclassified | Unclassified |
| OTU_3084 | Bacteria | Marinimicrobia_(SAR406_clade) | Marinimicrobia_(SAR406_clade) | Marinimicrobia_(SAR406_clade) | Marinimicrobia_(SAR406_clade) | Marinimicrobia_(SAR406_clade) |
| OTU_3101 | Archaea | Thermoplasmatota | Thermoplasmata | Marine_Group_II | Marine_Group_II | Marine_Group_II |
| OTU_3120 | Bacteria | Bacteroidota | Bacteroidia | Flavobacteriales | Flavobacteriaceae | NS4_marine_group |
| OTU_3129 | Bacteria | Proteobacteria | Alphaproteobacteria | SAR11_clade | Clade_II | Clade_II |
| OTU_3130 | Bacteria | Proteobacteria | Gammaproteobacteria | SAR86_clade | SAR86_clade | SAR86_clade |
| OTU_3133 | Bacteria | Proteobacteria | Alphaproteobacteria | Defluviicoccales | Unclassified | Unclassified |
| OTU_3146 | Bacteria | Bacteroidota | Bacteroidia | Flavobacteriales | Flavobacteriaceae | NS5_marine_group |
| OTU_3158 | Bacteria | Cyanobacteria | Cyanobacteriia | Synechococcales | Cyanobiaceae | Unclassified |
| OTU_3159 | Bacteria | Proteobacteria | Alphaproteobacteria | Rhodobacterales | Rhodobacteraceae | Unclassified |
| OTU_3163 | Bacteria | Proteobacteria | Alphaproteobacteria | Rhodobacterales | Rhodobacteraceae | Unclassified |
| OTU_3166 | Bacteria | Proteobacteria | Alphaproteobacteria | Rhodospirillales | AEGEAN-169_marine_group | AEGEAN-169_marine_group |
| OTU_3168 | Bacteria | Verrucomicrobiota | Kiritimatiellae | Kiritimatiellales | Kiritimatiellaceae | R76-B128 |
| OTU_3198 | Bacteria | Proteobacteria | Alphaproteobacteria | Rhodospirillales | AEGEAN-169_marine_group | AEGEAN-169_marine_group |
| OTU_3204 | Bacteria | Proteobacteria | Gammaproteobacteria | Thiotrichales | Thiotrichaceae | Unclassified |
| OTU_3233 | Bacteria | Proteobacteria | Alphaproteobacteria | SAR11_clade | Clade_I | Clade_Ia |
| OTU_3234 | Archaea | Crenarchaeota | Nitrososphaeria | Nitrosopumilales | Nitrosopumilaceae | Unclassified |
| OTU_3239 | Bacteria | Verrucomicrobiota | Verrucomicrobiae | Arctic97B-4_marine_group | Arctic97B-4_marine_group | Arctic97B-4_marine_group |
| OTU_3242 | Bacteria | Proteobacteria | Gammaproteobacteria | KI89A_clade | KI89A_clade | KI89A_clade |
| OTU_3247 | Bacteria | Bacteroidota | Bacteroidia | Flavobacteriales | Flavobacteriaceae | NS5_marine_group |
| OTU_3268 | Bacteria | Verrucomicrobiota | Verrucomicrobiae | Opitutales | Puniceicoccaceae | Pelagicoccus |
| OTU_3294 | Bacteria | Proteobacteria | Gammaproteobacteria | SAR86_clade | SAR86_clade | SAR86_clade |
| OTU_3311 | Bacteria | Proteobacteria | Alphaproteobacteria | SAR11_clade | Unclassified | Unclassified |
| OTU_3339 | Bacteria | Bacteroidota | Bacteroidia | Flavobacteriales | NS9_marine_group | NS9_marine_group |
| OTU_3354 | Bacteria | Cyanobacteria | Cyanobacteriia | Synechococcales | Cyanobiaceae | Synechococcus_CC9902 |
| OTU_3378 | Bacteria | Proteobacteria | Alphaproteobacteria | SAR11_clade | Clade_II | Clade_II |
| OTU_3396 | Bacteria | Marinimicrobia_(SAR406_clade) | Marinimicrobia_(SAR406_clade) | Marinimicrobia_(SAR406_clade) | Marinimicrobia_(SAR406_clade) | Marinimicrobia_(SAR406_clade) |
| OTU_3402 | Bacteria | Proteobacteria | Alphaproteobacteria | Thalassobaculales | Nisaeaceae | OM75_clade |
| OTU_3430 | Bacteria | Bacteroidota | Bacteroidia | Flavobacteriales | Flavobacteriaceae | NS4_marine_group |
| OTU_3450 | Bacteria | Proteobacteria | Alphaproteobacteria | Rhodospirillales | AEGEAN-169_marine_group | AEGEAN-169_marine_group |
| OTU_3514 | Bacteria | Proteobacteria | Gammaproteobacteria | SAR86_clade | SAR86_clade | SAR86_clade |
| OTU_3559 | Bacteria | Proteobacteria | Gammaproteobacteria | Cellvibrionales | Halieaceae | OM60(NOR5)_clade |
| OTU_3560 | Bacteria | Actinobacteriota | Acidimicrobiia | Microtrichales | Microtrichaceae | Sva0996_marine_group |
| OTU_3598 | Bacteria | Proteobacteria | Alphaproteobacteria | SAR11_clade | Unclassified | Unclassified |
| OTU_3625 | Bacteria | Proteobacteria | Gammaproteobacteria | OM182_clade | OM182_clade | OM182_clade |
| OTU_3644 | Bacteria | Proteobacteria | Alphaproteobacteria | SAR11_clade | Clade_I | Clade_Ia |
| OTU_3649 | Bacteria | Proteobacteria | Gammaproteobacteria | Cellvibrionales | Halieaceae | OM60(NOR5)_clade |
| OTU_3657 | Bacteria | Proteobacteria | Gammaproteobacteria | KI89A_clade | KI89A_clade | KI89A_clade |
| OTU_3662 | Bacteria | Bacteroidota | Bacteroidia | Flavobacteriales | NS9_marine_group | NS9_marine_group |
| OTU_3672 | Bacteria | Proteobacteria | Alphaproteobacteria | Puniceispirillales | SAR116_clade | Candidatus_Puniceispirillum |
| OTU_3688 | Bacteria | Bacteroidota | Bacteroidia | Flavobacteriales | Flavobacteriaceae | NS4_marine_group |
| OTU_3695 | Bacteria | Proteobacteria | Gammaproteobacteria | Oceanospirillales | Litoricolaceae | Litoricola |
| OTU_3733 | Bacteria | Proteobacteria | Gammaproteobacteria | Alteromonadales | Alteromonadaceae | Glaciecola |
| OTU_3756 | Bacteria | Proteobacteria | Alphaproteobacteria | SAR11_clade | Clade_I | Unclassified |
| OTU_3777 | Bacteria | Proteobacteria | Gammaproteobacteria | Cellvibrionales | Halieaceae | Unclassified |
| OTU_3807 | Bacteria | Planctomycetota | Phycisphaerae | Phycisphaerales | Phycisphaeraceae | CL500-3 |
| OTU_3827 | Bacteria | Actinobacteriota | Acidimicrobiia | Microtrichales | Microtrichaceae | Sva0996_marine_group |
| OTU_3836 | Bacteria | Bacteroidota | Bacteroidia | Flavobacteriales | Cryomorphaceae | Unclassified |
| OTU_3838 | Bacteria | Proteobacteria | Alphaproteobacteria | SAR11_clade | Clade_I | Unclassified |
| OTU_3858 | Bacteria | Chloroflexi | Dehalococcoidia | SAR202_clade | SAR202_clade | SAR202_clade |
| OTU_3868 | Bacteria | Proteobacteria | Alphaproteobacteria | SAR11_clade | Clade_I | Unclassified |
| OTU_3895 | Bacteria | Bacteroidota | Bacteroidia | Flavobacteriales | Flavobacteriaceae | NS4_marine_group |
| OTU_3898 | Bacteria | Bacteroidota | Bacteroidia | Flavobacteriales | Flavobacteriaceae | NS4_marine_group |
| OTU_3904 | Bacteria | SAR324_clade(Marine_group_B) | SAR324_clade(Marine_group_B) | SAR324_clade(Marine_group_B) | SAR324_clade(Marine_group_B) | SAR324_clade(Marine_group_B) |
| OTU_3909 | Bacteria | Bdellovibrionota | Bdellovibrionia | Bdellovibrionales | Bdellovibrionaceae | OM27_clade |
| OTU_3916 | Bacteria | Proteobacteria | Alphaproteobacteria | Puniceispirillales | SAR116_clade | Candidatus_Puniceispirillum |
| OTU_3924 | Bacteria | Bacteroidota | Bacteroidia | Flavobacteriales | Flavobacteriaceae | NS4_marine_group |
| OTU_3952 | Bacteria | Cyanobacteria | Cyanobacteriia | Synechococcales | Cyanobiaceae | Unclassified |
| OTU_3961 | Bacteria | Bacteroidota | Bacteroidia | Flavobacteriales | Cryomorphaceae | Unclassified |
| OTU_3967 | Bacteria | Nitrospinota | Nitrospinia | Nitrospinales | Nitrospinaceae | LS-NOB |
| OTU_3973 | Bacteria | Proteobacteria | Alphaproteobacteria | SAR11_clade | Clade_I | Unclassified |
| OTU_3984 | Bacteria | Proteobacteria | Gammaproteobacteria | SAR86_clade | SAR86_clade | SAR86_clade |
| OTU_3994 | Bacteria | Cyanobacteria | Cyanobacteriia | Synechococcales | Cyanobiaceae | Prochlorococcus_MIT9313 |
| OTU_4006 | Bacteria | Actinobacteriota | Acidimicrobiia | Actinomarinales | Actinomarinaceae | Candidatus_Actinomarina |
| OTU_4030 | Bacteria | Proteobacteria | Alphaproteobacteria | Rhodospirillales | AEGEAN-169_marine_group | AEGEAN-169_marine_group |
| OTU_4040 | Bacteria | Proteobacteria | Gammaproteobacteria | Cellvibrionales | Halieaceae | Unclassified |
| OTU_4047 | Bacteria | Verrucomicrobiota | Verrucomicrobiae | Arctic97B-4_marine_group | Arctic97B-4_marine_group | Arctic97B-4_marine_group |
| OTU_4056 | Bacteria | Proteobacteria | Alphaproteobacteria | SAR11_clade | Clade_II | Clade_II |
| OTU_4082 | Bacteria | Proteobacteria | Alphaproteobacteria | Puniceispirillales | SAR116_clade | SAR116_clade |
| OTU_4084 | Bacteria | Bacteroidota | Bacteroidia | Flavobacteriales | Flavobacteriaceae | Formosa |
| OTU_4100 | Bacteria | Proteobacteria | Alphaproteobacteria | SAR11_clade | Unclassified | Unclassified |
| OTU_4106 | Bacteria | Proteobacteria | Alphaproteobacteria | Sphingomonadales | Sphingomonadaceae | Erythrobacter |
| OTU_4114 | Archaea | Thermoplasmatota | Thermoplasmata | Thermoplasmata | Thermoplasmata | Marine_Group_III |
| OTU_4145 | Bacteria | Bacteroidota | Bacteroidia | Flavobacteriales | Flavobacteriaceae | NS5_marine_group |
| OTU_4148 | Archaea | Crenarchaeota | Nitrososphaeria | Nitrosopumilales | Nitrosopumilaceae | Candidatus_Nitrosopumilus |
| OTU_4177 | Bacteria | Proteobacteria | Alphaproteobacteria | SAR11_clade | Clade_I | Unclassified |
| OTU_4180 | Bacteria | Verrucomicrobiota | Verrucomicrobiae | Opitutales | Puniceicoccaceae | Coraliomargarita |
| OTU_4185 | Bacteria | Proteobacteria | Alphaproteobacteria | SAR11_clade | Clade_I | Clade_Ia |
| OTU_4188 | Bacteria | Proteobacteria | Alphaproteobacteria | SAR11_clade | Unclassified | Unclassified |
| OTU_4201 | Bacteria | Cyanobacteria | Cyanobacteriia | Synechococcales | Cyanobiaceae | Synechococcus_CC9902 |
| OTU_4224 | Archaea | Crenarchaeota | Nitrososphaeria | Nitrosopumilales | Nitrosopumilaceae | Candidatus_Nitrosopumilus |
| OTU_4228 | Bacteria | Planctomycetota | OM190 | OM190 | OM190 | OM190 |
| OTU_4254 | Bacteria | Proteobacteria | Alphaproteobacteria | Defluviicoccales | Unclassified | Unclassified |
| OTU_4281 | Bacteria | Bacteroidota | Bacteroidia | Flavobacteriales | NS7_marine_group | NS7_marine_group |
| OTU_4292 | Bacteria | Proteobacteria | Gammaproteobacteria | Alteromonadales | Alteromonadaceae | Alteromonas |
| OTU_4302 | Archaea | Thermoplasmatota | Thermoplasmata | Marine_Group_II | Marine_Group_II | Marine_Group_II |
| OTU_4374 | Bacteria | Bacteroidota | Bacteroidia | Flavobacteriales | Flavobacteriaceae | Tenacibaculum |
| OTU_4414 | Bacteria | Proteobacteria | Gammaproteobacteria | Vibrionales | Vibrionaceae | Unclassified |
| OTU_4423 | Bacteria | Proteobacteria | Gammaproteobacteria | UBA10353_marine_group | UBA10353_marine_group | UBA10353_marine_group |
| OTU_4430 | Bacteria | Proteobacteria | Gammaproteobacteria | Cellvibrionales | Porticoccaceae | SAR92_clade |
| OTU_4485 | Bacteria | Proteobacteria | Alphaproteobacteria | SAR11_clade | Clade_II | Clade_II |
| OTU_4499 | Bacteria | Proteobacteria | Alphaproteobacteria | Rhodospirillales | AEGEAN-169_marine_group | AEGEAN-169_marine_group |
| OTU_4519 | Archaea | Crenarchaeota | Nitrososphaeria | Nitrosopumilales | Nitrosopumilaceae | Unclassified |
| OTU_4553 | Bacteria | Bacteroidota | Bacteroidia | Flavobacteriales | Flavobacteriaceae | NS5_marine_group |
| OTU_4579 | Bacteria | Proteobacteria | Gammaproteobacteria | SAR86_clade | SAR86_clade | SAR86_clade |
| OTU_4608 | Bacteria | Proteobacteria | Alphaproteobacteria | SAR11_clade | Clade_I | Unclassified |
| OTU_4613 | Bacteria | Bacteroidota | Bacteroidia | Flavobacteriales | Flavobacteriaceae | NS4_marine_group |
| OTU_4619 | Bacteria | Bacteroidota | Bacteroidia | Flavobacteriales | NS7_marine_group | NS7_marine_group |
| OTU_4624 | Bacteria | Proteobacteria | Alphaproteobacteria | SAR11_clade | Clade_I | Unclassified |
| OTU_4630 | Bacteria | Proteobacteria | Alphaproteobacteria | SAR11_clade | Clade_I | Unclassified |
| OTU_4645 | Bacteria | Verrucomicrobiota | Verrucomicrobiae | Opitutales | Puniceicoccaceae | MB11C04_marine_group |
| OTU_4651 | Bacteria | Proteobacteria | Gammaproteobacteria | HOC36 | HOC36 | HOC36 |
| OTU_4656 | Bacteria | Proteobacteria | Alphaproteobacteria | Rhodospirillales | AEGEAN-169_marine_group | AEGEAN-169_marine_group |
| OTU_4676 | Bacteria | Proteobacteria | Alphaproteobacteria | SAR11_clade | Unclassified | Unclassified |
| OTU_4698 | Archaea | Thermoplasmatota | Thermoplasmata | Marine_Group_II | Marine_Group_II | Marine_Group_II |
| OTU_4700 | Bacteria | Verrucomicrobiota | Verrucomicrobiae | Arctic97B-4_marine_group | Arctic97B-4_marine_group | Arctic97B-4_marine_group |
| OTU_4701 | Bacteria | Proteobacteria | Alphaproteobacteria | SAR11_clade | Clade_I | Clade_I |
| OTU_4710 | Bacteria | Bacteroidota | Bacteroidia | Flavobacteriales | Flavobacteriaceae | NS4_marine_group |
| OTU_4732 | Bacteria | Marinimicrobia_(SAR406_clade) | Marinimicrobia_(SAR406_clade) | Marinimicrobia_(SAR406_clade) | Marinimicrobia_(SAR406_clade) | Marinimicrobia_(SAR406_clade) |
| OTU_4738 | Bacteria | Verrucomicrobiota | Verrucomicrobiae | Opitutales | Puniceicoccaceae | MB11C04_marine_group |
| OTU_4739 | Bacteria | Actinobacteriota | Acidimicrobiia | Actinomarinales | Actinomarinaceae | Candidatus_Actinomarina |
| OTU_4749 | Bacteria | Proteobacteria | Alphaproteobacteria | Puniceispirillales | SAR116_clade | SAR116_clade |
| OTU_4784 | Bacteria | Proteobacteria | Gammaproteobacteria | Oceanospirillales | Litoricolaceae | Litoricola |
| OTU_4802 | Bacteria | Proteobacteria | Alphaproteobacteria | Sphingomonadales | Sphingomonadaceae | Erythrobacter |
| OTU_4859 | Bacteria | Proteobacteria | Gammaproteobacteria | Vibrionales | Vibrionaceae | Vibrio |
| OTU_4871 | Bacteria | Proteobacteria | Alphaproteobacteria | SAR11_clade | Clade_I | Unclassified |
| OTU_4908 | Bacteria | Bacteroidota | Bacteroidia | Flavobacteriales | Flavobacteriaceae | NS4_marine_group |
| OTU_4947 | Bacteria | Proteobacteria | Alphaproteobacteria | SAR11_clade | Clade_I | Unclassified |
| OTU_4997 | Bacteria | Proteobacteria | Alphaproteobacteria | Rhodospirillales | AEGEAN-169_marine_group | AEGEAN-169_marine_group |
| OTU_5030 | Bacteria | Proteobacteria | Gammaproteobacteria | UBA10353_marine_group | UBA10353_marine_group | UBA10353_marine_group |
| OTU_5049 | Bacteria | Bacteroidota | Rhodothermia | Balneolales | Balneolaceae | Balneola |
| OTU_5053 | Bacteria | Proteobacteria | Alphaproteobacteria | Rhodobacterales | Rhodobacteraceae | Yoonia-Loktanella |
| OTU_5084 | Bacteria | Proteobacteria | Gammaproteobacteria | HgCo23 | HgCo23 | HgCo23 |
| OTU_5107 | Bacteria | Proteobacteria | Alphaproteobacteria | Rhodospirillales | AEGEAN-169_marine_group | AEGEAN-169_marine_group |
| OTU_5171 | Bacteria | Actinobacteriota | Acidimicrobiia | Actinomarinales | Actinomarinaceae | Candidatus_Actinomarina |
| OTU_5187 | Bacteria | Marinimicrobia_(SAR406_clade) | Marinimicrobia_(SAR406_clade) | Marinimicrobia_(SAR406_clade) | Marinimicrobia_(SAR406_clade) | Marinimicrobia_(SAR406_clade) |
| OTU_5189 | Bacteria | Cyanobacteria | Cyanobacteriia | Synechococcales | Cyanobiaceae | Synechococcus_CC9902 |
| OTU_5194 | Bacteria | Proteobacteria | Alphaproteobacteria | Puniceispirillales | SAR116_clade | SAR116_clade |
| OTU_5196 | Bacteria | Proteobacteria | Alphaproteobacteria | SAR11_clade | Unclassified | Unclassified |
| OTU_5205 | Bacteria | Proteobacteria | Alphaproteobacteria | SAR11_clade | Clade_I | Clade_Ia |
| OTU_5278 | Bacteria | Proteobacteria | Alphaproteobacteria | SAR11_clade | Clade_I | Clade_Ib |
| OTU_5279 | Bacteria | Bacteroidota | Bacteroidia | Flavobacteriales | Flavobacteriaceae | NS5_marine_group |
| OTU_5300 | Bacteria | Proteobacteria | Gammaproteobacteria | Burkholderiales | Methylophilaceae | OM43_clade |
| OTU_5328 | Bacteria | Actinobacteriota | Acidimicrobiia | Microtrichales | Microtrichaceae | Sva0996_marine_group |
| OTU_5353 | Bacteria | Proteobacteria | Gammaproteobacteria | SAR86_clade | SAR86_clade | SAR86_clade |
| OTU_5366 | Bacteria | PAUC34f | PAUC34f | PAUC34f | PAUC34f | PAUC34f |
| OTU_5403 | Bacteria | Actinobacteriota | Acidimicrobiia | Microtrichales | Microtrichaceae | Sva0996_marine_group |
| OTU_5441 | Bacteria | Marinimicrobia_(SAR406_clade) | Marinimicrobia_(SAR406_clade) | Marinimicrobia_(SAR406_clade) | Marinimicrobia_(SAR406_clade) | Marinimicrobia_(SAR406_clade) |
| OTU_5450 | Bacteria | Bacteroidota | Bacteroidia | Cytophagales | Cyclobacteriaceae | Marinoscillum |
| OTU_5465 | Bacteria | Bacteroidota | Bacteroidia | Flavobacteriales | NS9_marine_group | NS9_marine_group |
| OTU_5475 | Bacteria | Unclassified | Unclassified | Unclassified | Unclassified | Unclassified |
| OTU_5484 | Bacteria | Planctomycetota | OM190 | OM190 | OM190 | OM190 |
| OTU_5505 | Bacteria | Proteobacteria | Alphaproteobacteria | SAR11_clade | Clade_I | Unclassified |
| OTU_5537 | Bacteria | Bacteroidota | Bacteroidia | Flavobacteriales | Flavobacteriaceae | NS4_marine_group |
| OTU_5551 | Bacteria | Bacteroidota | Bacteroidia | Flavobacteriales | Flavobacteriaceae | NS5_marine_group |
| OTU_5563 | Bacteria | Proteobacteria | Gammaproteobacteria | KI89A_clade | KI89A_clade | KI89A_clade |
| OTU_5565 | Bacteria | Proteobacteria | Alphaproteobacteria | Rickettsiales | S25-593 | S25-593 |
| OTU_5587 | Bacteria | Proteobacteria | Alphaproteobacteria | SAR11_clade | Unclassified | Unclassified |
| OTU_5622 | Bacteria | Proteobacteria | Gammaproteobacteria | Burkholderiales | MWH-UniP1_aquatic_group | MWH-UniP1_aquatic_group |
| OTU_5626 | Bacteria | Proteobacteria | Alphaproteobacteria | SAR11_clade | Clade_I | Unclassified |
| OTU_5649 | Bacteria | Proteobacteria | Alphaproteobacteria | SAR11_clade | Clade_II | Clade_II |
| OTU_5655 | Archaea | Thermoplasmatota | Thermoplasmata | Marine_Group_II | Marine_Group_II | Marine_Group_II |
| OTU_5673 | Bacteria | Proteobacteria | Alphaproteobacteria | SAR11_clade | Unclassified | Unclassified |
| OTU_5695 | Archaea | Crenarchaeota | Nitrososphaeria | Nitrosopumilales | Nitrosopumilaceae | Candidatus_Nitrosopumilus |
| OTU_5700 | Bacteria | Proteobacteria | Alphaproteobacteria | Thalassobaculales | Nisaeaceae | OM75_clade |
| OTU_5709 | Bacteria | Marinimicrobia_(SAR406_clade) | Marinimicrobia_(SAR406_clade) | Marinimicrobia_(SAR406_clade) | Marinimicrobia_(SAR406_clade) | Marinimicrobia_(SAR406_clade) |
| OTU_5822 | Bacteria | Proteobacteria | Alphaproteobacteria | Puniceispirillales | SAR116_clade | Candidatus_Puniceispirillum |
| OTU_5827 | Bacteria | Proteobacteria | Alphaproteobacteria | SAR11_clade | Clade_I | Clade_I |
| OTU_5830 | Bacteria | Proteobacteria | Alphaproteobacteria | SAR11_clade | Clade_I | Unclassified |
| OTU_5869 | Bacteria | Proteobacteria | Alphaproteobacteria | Rhizobiales | Stappiaceae | Stappiaceae |
| OTU_5900 | Bacteria | Planctomycetota | Planctomycetes | Planctomycetales | Unclassified | Unclassified |
| OTU_5909 | Bacteria | Proteobacteria | Alphaproteobacteria | Rhodobacterales | Rhodobacteraceae | Unclassified |
| OTU_5919 | Bacteria | Proteobacteria | Gammaproteobacteria | Oceanospirillales | Pseudohongiellaceae | Pseudohongiella |
| OTU_5953 | Bacteria | Verrucomicrobiota | Verrucomicrobiae | Opitutales | Puniceicoccaceae | Coraliomargarita |
| OTU_5978 | Bacteria | Proteobacteria | Alphaproteobacteria | Rhodospirillales | Magnetospiraceae | Unclassified |
| OTU_6008 | Bacteria | Proteobacteria | Gammaproteobacteria | Cellvibrionales | Halieaceae | Unclassified |
| OTU_6020 | Archaea | Crenarchaeota | Nitrososphaeria | Nitrosopumilales | Nitrosopumilaceae | Unclassified |
| OTU_6030 | Bacteria | Cyanobacteria | Cyanobacteriia | Synechococcales | Cyanobiaceae | Prochlorococcus_MIT9313 |
| OTU_6053 | Bacteria | Proteobacteria | Alphaproteobacteria | Rhodospirillales | AEGEAN-169_marine_group | AEGEAN-169_marine_group |
| OTU_6196 | Bacteria | Cyanobacteria | Cyanobacteriia | Synechococcales | Cyanobiaceae | Unclassified |
| OTU_6236 | Bacteria | Proteobacteria | Alphaproteobacteria | Rhodobacterales | Rhodobacteraceae | Unclassified |
| OTU_6240 | Bacteria | Proteobacteria | Alphaproteobacteria | Rhodospirillales | AEGEAN-169_marine_group | AEGEAN-169_marine_group |
| OTU_6259 | Bacteria | Proteobacteria | Gammaproteobacteria | SAR86_clade | SAR86_clade | SAR86_clade |
| OTU_6320 | Bacteria | Proteobacteria | Alphaproteobacteria | SAR11_clade | Clade_II | Clade_II |
| OTU_6329 | Bacteria | Proteobacteria | Gammaproteobacteria | Cellvibrionales | Porticoccaceae | SAR92_clade |
| OTU_6362 | Bacteria | Proteobacteria | Gammaproteobacteria | Thiotrichales | Thiotrichaceae | Unclassified |
| OTU_6379 | Bacteria | Proteobacteria | Alphaproteobacteria | Rhodospirillales | AEGEAN-169_marine_group | AEGEAN-169_marine_group |
| OTU_6408 | Bacteria | Verrucomicrobiota | Verrucomicrobiae | Opitutales | Puniceicoccaceae | MB11C04_marine_group |
| OTU_6452 | Bacteria | Verrucomicrobiota | Verrucomicrobiae | Verrucomicrobiales | DEV007 | DEV007 |
| OTU_6461 | Bacteria | Verrucomicrobiota | Verrucomicrobiae | Opitutales | Puniceicoccaceae | Lentimonas |
| OTU_6483 | Bacteria | Proteobacteria | Alphaproteobacteria | SAR11_clade | Clade_I | Unclassified |
| OTU_6517 | Bacteria | Cyanobacteria | Cyanobacteriia | Synechococcales | Cyanobiaceae | Synechococcus_CC9902 |
| OTU_6531 | Bacteria | Actinobacteriota | Acidimicrobiia | Microtrichales | Microtrichaceae | Sva0996_marine_group |
| OTU_6539 | Bacteria | Bacteroidota | Bacteroidia | Flavobacteriales | Unclassified | Unclassified |
| OTU_6576 | Bacteria | Proteobacteria | Alphaproteobacteria | SAR11_clade | Clade_I | Unclassified |

### 3.7. Neural gas analysis


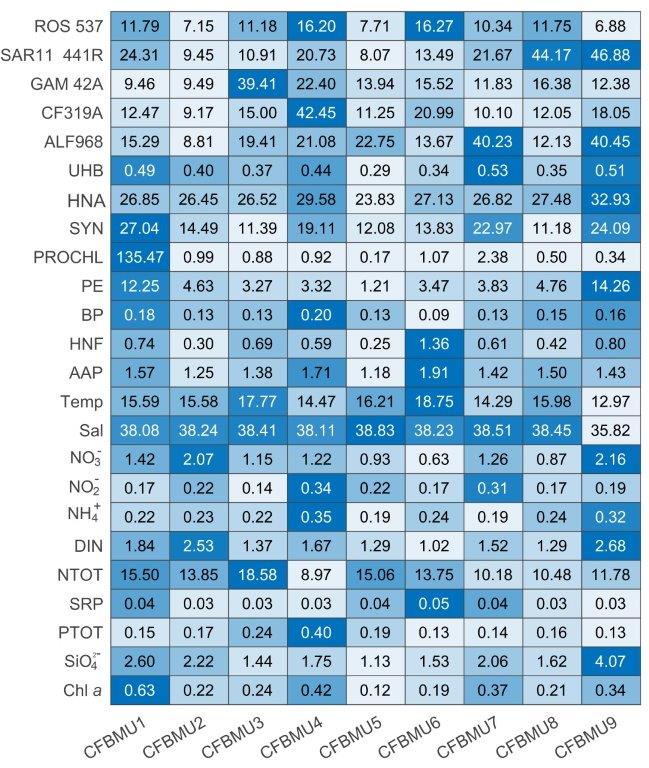


SI Figure 5. Bacterial groups (ROS537, SAR11_441R, GAM42A, CF319A, ALF968), biological parameters (UHB, HIGH, SYN, PROCHL, PE, BP, HNF, AAP) and environmental factors (Temp, Sal, NO_3_^-^, NO_2_^-^, NH_4_^+^, DIN, NTOT, SRP, PTOT, SiO_4_^2-^, Chl *a*) calculated for CF BMU neural gas model. Shades from white to dark blue indicate the range of the mean from the smallest (white) to the largest (dark blue) value for each parameter in each BMU.


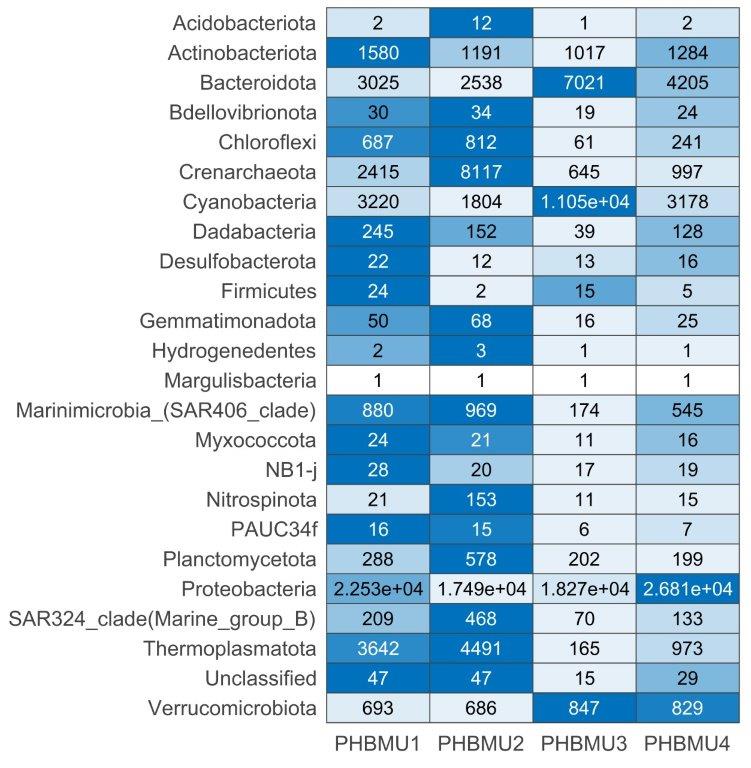


SI Figure 6. Phylum relative abundance classified within four clusters using neural gas. Shades from white to dark blue indicate the range of values from smallest (white) to largest (dark blue) for each parameter in each BMU.


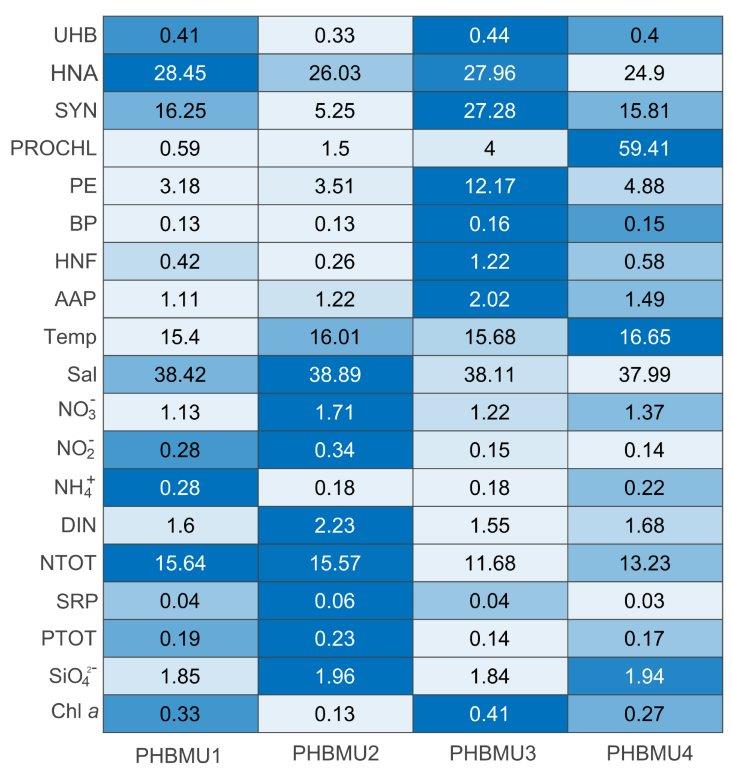


SI Figure 7. Biological parameters (UHB, HIGH, SYN, PROCHL, PE, BP, HNF, AAP) and environmental factors (Temp, Sal, NO_3_^-^, NO_2_^-^, NH_4_^+^, DIN, NTOT, SRP, PTOT, SiO_4_^2-^, Chl *a*) calculated for PHBMU neural gas model. Shades from white to dark blue indicate the range of the mean from the smallest (white) to the largest (dark blue) value for each parameter in each BMU.

## References

1. Koroleff, F. & Grasshoff, K. Methods of seawater analysis. in (ed. Grasshoff, K., Wiley, Verlag, 1976).

2. Justić, D., Rabalais, N. N., Turner, R. E. & Dortch, Q. Changes in nutrient structure of river-dominated coastal waters: stoichiometric nutrient balance and its consequences. *Estuar. Coast. Shelf Sci.* **40(3)**, 339-356 (1995).

3. Strickland, J. D. H. & Parsons, T. R. A practical handbook of seawater analysis.

(second ed. **167**, Fisheries Research Board of Canada, Ottawa, 1972).

4. Gasol, J. M. & Morán, X. A. G. Flow cytometric determination of microbial abundances and its use to obtain indices of community structure and relative activity. *In Hydrocarbon and lipid microbiology protocols.* (Springer, Berlin, Heidelberg, 2015) 159-187.

5. Mašín, M. et al. Seasonal changes and diversity of aerobic anoxygenic phototrophs in the Baltic Sea. *Aquat. Microb. Ecol*. **45**, 247–254. https://doi.org/10.3354/ame045247 (2006).

6. Fuhrman, J. A. & Azam, F. Thymidine incorporation as a measure of heterotrophic bacterioplankton production in marine surface waters: evaluation and field results. *Mar. Biol.* **66(2)**, 109-120 (1982).

7. Pernthaler, A., Pernthaler, J. & Amann, R. Fluorescence in situ hybridization and catalyzed reporter deposition for the identification of marine bacteria. *Appl. Environ. Microbiol*. **68(6)**, 3094–3101. https://doi.org/10.1128/AEM.68.6.3094-3101.2002 (2002).
